# Physicochemical Characterisation of Ceftobiprole and Investigation of the Biological Properties of Its Cyclodextrin-Based Delivery System

**DOI:** 10.3390/ijms262412108

**Published:** 2025-12-16

**Authors:** Dariusz Boczar, Wojciech Bocian, Krystian Małek, Małgorzata Milczarek, Agnieszka Ewa Laudy, Katarzyna Michalska

**Affiliations:** 1 Department of Synthetic Drugs, National Medicines Institute, Chełmska 30/34, 00-725 Warsaw, Poland; 2 Laboratory for Analysis of Bioactive Compounds, Institute of Organic Chemistry, Polish Academy of Sciences, Kasprzaka 44/52, 01-224 Warsaw, Poland; wo.bocian@gmail.com; 3 Department of Biomedical Research, National Medicines Institute, Chełmska 30/34, 00-725 Warsaw, Poland; k.malek@nil.gov.pl (K.M.); m.milczarek@nil.gov.pl (M.M.); 4 Department of Pharmaceutical Microbiology and Bioanalysis, Medical University of Warsaw, Banacha 1b, 02-097 Warsaw, Poland; alaudy@wp.pl

**Keywords:** ceftobiprole, SBE-β-CD, solubility, NMR, capillary electrophoresis, molecular dynamics, drug delivery system, antibacterial activity, cytotoxicity

## Abstract

Ceftobiprole is a novel and promising antibiotic; however, the direct pharmacological use of its native form is limited by its low water solubility. The first part of this study provides a deeper insight into the physicochemical properties of this drug. One- and two-dimensional nuclear magnetic resonance (NMR) spectra in D_2_O were recorded, and a complete assignment of ^1^H and ^13^C signals was achieved with the support of quantum mechanical calculations. The combined results from capillary electrophoresis and NMR confirmed the cationic nature of ceftobiprole at pH values well below 3 and the protonation of the secondary amino group, thus supporting the theoretically predicted dominant protonation states. Molecular dynamics simulations revealed that zwitterionic ceftobiprole molecules associate through hydrogen bonding, whereas in the cationic form, the attractive forces involve weaker π-π and stacking interactions. The use of ceftobiprole in its native form in pharmaceutical formulations was made possible through the development of a novel freeze-dried cyclodextrin-based delivery system. Consequently, the second part of this article focuses on evaluating the biological properties of this system (ceftobiprole/maleic acid/sulfobutylether-β-cyclodextrin in a molar ratio of 1:25:4), including its antibacterial activity against the most common pneumonia-causing pathogens and its cytotoxicity towards normal and cancer cells.

## 1. Introduction

**Scheme 1:**
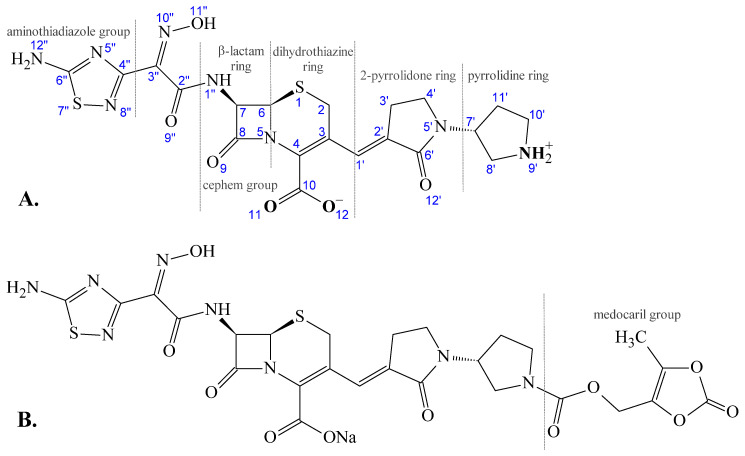
Molecular structure of (**A**) ceftobiprole (CBP) in its zwitterionic form, accompanied with atom labels used in the text, and (**B**) ceftobiprole medocaril sodium salt (CBP-M).

Ceftobiprole (CBP, C_20_H_22_N_8_O_6_S_2_, Scheme 1A) is a relatively new antibiotic, belonging to the fifth generation of cepahlosporins, active against methicillin-resistant *Staphylococcus aureus* (MRSA) and penicillin-resistant *Streptococcus pneumoniae* (PRSP) [1]. It was first introduced into clinical practice in Canada on 26 June 2008 under the trade name Zeftera as ceftobiprole medocaril sodium salt (CBP-M) [2]. Its regulatory approval process was complex, and the spectrum of approved indications has been revised in response to emerging clinical trial data.

As of 2024, CBP-M has distinct approvals in the United States and Europe. In the United States, the Food and Drug Administration (FDA) authorizes its use for: *S. aureus* bloodstream infections, including right-sided infective endocarditis caused by *S. aureus* strains both sensitive and resistant to methicillin, acute bacterial skin and skin structure infections (ABSSSI) in adults, and community-acquired pneumonia (CAP) in adults and pediatric patients aged 3 months to under 18 years [3]. These approvals are based on recent Phase 3 clinical trials confirming efficacy and safety [3]. In contrast, in Europe, CBP-M is primarily indicated for hospital-acquired pneumonia (HAP) in adults, excluding ventilator-associated pneumonia (VAP), and in certain countries, also for CAP in adults [4,5,6]. Approvals for bloodstream infections or ABSSSI have not been granted, as European regulators judged the submitted evidence insufficient [7,8].

Despite its structural features CBP exhibits unexpectedly poor solubility in water (≈0.05 mg/mL), which is not yet fully understood. This anomaly prompted us to investigate its physicochemical properties in detail to better understand both its solubility and its behaviour in drug formulations.

Commercially marketed drug product Zevtera contains a prodrug, CBP as a medocaril sodium salt (Scheme 1B), which undergoes in vivo biotransformation to the active form after its infusion to the circulatory system. However, the efficiency of this biotransformation may vary between individuals, depending on health status and age. To address low aqueous solubility of the active form of CBP, a drug delivery system was developed by freeze-drying CBP with sulfobutylether-β-cyclodextrin (SBE-β-CD) and one of the selected model acids, differing in strength and volatility, as described in detail in [9]. The most promising formulation was obtained using CBP, maleic acid (MA), and SBE-β-CD in a molar ratio of 1:25:4, which allowed for an approximately 300-fold increase in solubility up to 14 mg/mL. The prepared system was characterized by satisfactory chemical and physical stability after eight months of storage in a freezer. The previous paper [9] addressed only the physicochemical properties, highlighting the need for further research on the developed system, such as examining the biological activity.

The aim of this work is to gain deeper insight into the properties of CBP and to provide a more detailed mechanistic explanation of the observed phenomena. The first part of the article focuses on the structural, spectral, and physicochemical properties of CBP, including the interpretation of nuclear magnetic resonance (NMR) spectra supported by quantum-mechanical density functional theory (DFT) calculations. This work also presents the studies on the solubility of CBP in various organic solvents, its acid-base behaviour, and the prediction of intermolecular interactions by molecular dynamics (MD) simulations to better explain the behaviour of CBP molecules in aqueous solutions. The second part discusses the evaluation of a novel drug delivery system with improved solubility, assessed in terms of cytotoxicity and antibacterial activity.

## 2. Results and Discussion

### 2.1. DFT-Assisted NMR Analysis

In several patents [10,11], lists of the ^1^H NMR signals recorded for CBP dissolved in DMSO can be found; however, the assignment of multiplets to individual atoms or groups is missing. In patent [12], the summary of ^1^H NMR signals is accompanied by some atom numbers; however, this report does not include a scheme of the structural formula of the CBP molecule with the corresponding atom labels marked, which makes it difficult for the scientific community to interpret the spectra. To the best of our knowledge, the interpretation of NMR spectra in D_2_O has not been reported so far. On the other hand, the use of D_2_O as a solvent has now been made possible thanks to the significantly improved solubility of CBP (to 3.75 mM = 2 mg/mL), achieved by lyophilisation in acidic conditions (0.1 M HCOOH) [9], thus allowing satisfactory sensitivity of the spectra. Moreover, this work discusses not only ^1^H spectra but also ^13^C and correlation 2D ones (in the Supplementary Material).

**Figure 1:**
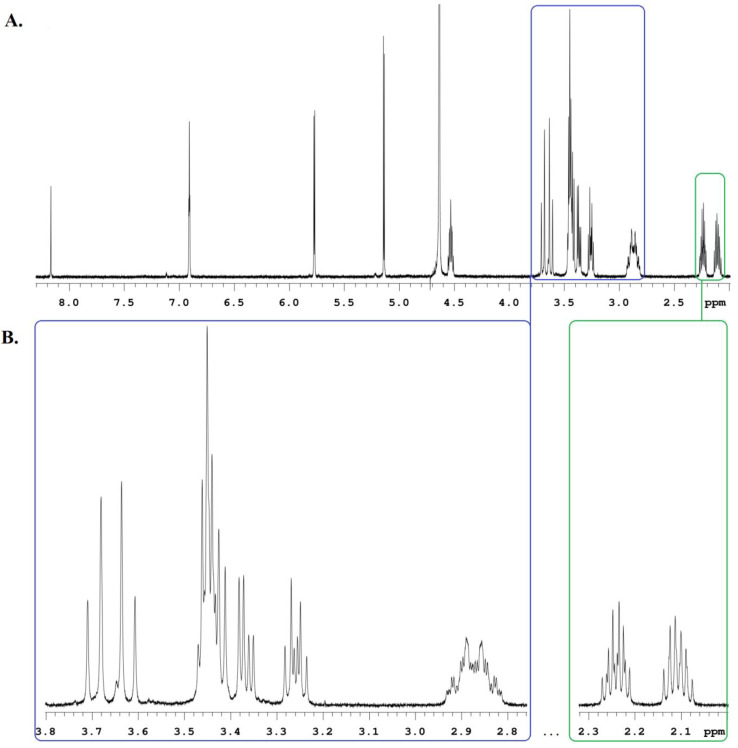
^1^H NMR spectrum of a 3.75 mM solution in D_2_O of CBP freeze-dried in 0.1 M HCOOH: (**A**) full spectrum in the range 2.0–8.3 ppm, and (**B**) magnified view of the aliphatic region for better visualization of multiplets.

It should be noted that 6 out of 22 hydrogen atoms in the molecule undergo a quick exchange with deuterium from the solvent (they are called labile), and thus are undetectable in NMR spectra in aqueous environment in our conditions. These include amine protons (two H12″, two H9′ and one H1″) as well as oxime proton H11″. The lack of protons in aromatic thiadiazole ring accompanied with the lability of H12″, H11″ and H1″ renders the entire substituent at C7 (attached to β-lactam ring) “invisible” in the ^1^H NMR spectrum recorded in D_2_O. For this reason, the assignment of almost all carbon atoms in this substituent could not be established from the correlation ^1^H–^13^C spectra and thus had to be supported by theoretical calculations of chemical shifts using DFT. Similarly, the assignment of the C3, C4 and C10 quaternary carbon atoms was impossible based solely on NMR measurements. For C3 and C4, only ambiguous ^1^H–^13^C correlations from two H2 atoms (via three and two bonds) were observed in the HMBC spectra, which did not enable distinguishing between the C3 and C4 NMR signals. In turn, the C10 carbon atom in the carboxyl group is simply too far from any observable hydrogen atom to give a ^1^H–^13^C correlation. The summary of both experimental and computed chemical shifts is shown in Table 1 with atom labels explained in Scheme 1. Figure 1 shows the ^1^H NMR spectrum of CBP with magnification for the most interesting ranges, while the ^13^C and correlation spectra can be found in the Supplementary Material along with a detailed description of the interpretation.

In theoretical DFT calculations, it is important to consider that CBP is a relatively large molecule with many freely rotating chemical bonds and flexible rings. Therefore, a complex equilibrium of many conformations of this molecule in solution is expected. To calculate NMR chemical shifts with sufficient accuracy, it was necessary to include at least some of the most probable conformers. Therefore, NMR shielding calculations were performed for forty selected CBP conformers ordered by increasing energy. NMR chemical shifts were then calculated using a linear fit of shieldings (averaged over a given number of structures) to the experimental data (Table S1). The best fit (Figure S7, R^2^ = 0.9989) was obtained when the shieldings were averaged for fifteen CBP conformers without applying any weights (Table S1). Meanwhile, when Boltzmann population weights are applied, the R^2^ value levelled off after averaging only four structures, reflecting the fact that Boltzmann weights became negligible for conformers beyond the fifth (as presented in Table S1). This is not very surprising, as it is well known that quantum computations are limited in accurately describing with the Boltzmann distribution of conformations in solution [13]. The detailed description of calculations performed with and without applying the Boltzmann weights can be found in the Supplementary Material.

It is worth mentioning that the performed DFT calculations of NMR shieldings were accompanied by determining the vibrational frequencies of each analyzed CBP conformer. This ensured that the proposed conformer structures corresponded to true minima on the potential energy surface. Knowledge of the frequencies also enabled the estimation of the zero-point vibrational energy (ZPVE), which was subsequently used to obtain more accurate values of energy differences, necessary for correctly determining the Boltzmann population weights (Table S1). Moreover, the vibrational analysis performed for the 15 lowest-energy conformers allowed the simulation of the theoretical IR spectrum, which was then compared with the experimental ATR-FTIR spectrum (Figure S8). However, the agreement between the theoretical and experimental IR spectra was found unsatisfactory, which was attributed to the presence of intermolecular hydrogen bonds in the solid sample, both in its amorphous and crystalline forms, not covered in the performed DFT calculations.

### 2.2. Physicochemical Properties of CBP

#### 2.2.1. Considerations on the Hydrophobic Behaviour and the Solubility of CBP

The low water solubility of the active form of CBP (≈0.05 mg/mL) is difficult to rationalize based solely on its structural formula, as the molecule contains several ionisable functional groups together with many hydrogen bond donors and acceptors. While its poor aqueous solubility could suggest lipophilicity, calculated logP value is negative [14], which indicates the opposite. Since logP represents the logarithm of the partition coefficient between octanol and water, a negative logarithm value implies this coefficient below one, meaning that CBP preferentially dissolves in water rather than in octanol. This apparent contradiction was also evident during the development of a high performance liquid chromatographic (HPLC) method for CBP determination [15], in which retention on hydrophobic stationary phases (C18 or biphenyl) required an initial mobile phase to be 100% aqueous (buffer of pH 5.8). Even the presence of 5% acetonitrile in mobile phase, standard in many HPLC analytical methods, caused CBP to elute at the column dead volume time, which suggests weak interactions with the hydrophobic column packing and thus a pronounced hydrophilic character of the analyte [15]. In another experiment, CBP was extensively mixed with various cyclodextrins (CDs) in an attempt to improve solubility. However, this led to only a slight increase [16], whereas similar strategies with other compounds typically yield substantial solubility enhancement [17].

In this work, to further investigate the properties of CBP, its solubility in 18 selected organic solvents was assessed using a previously developed selective HPLC method [15]. The tested solvents encompassed polar protic (methanol, ethanol, *n*-butanol, propylene glycol), polar aprotic (dimethyl sulfoxide (DMSO), acetonitrile, dimethylformamide (DMF), acetone, ethyl acetate, tetrahydrofuran (THF), dichloromethane, dioxane, dimethyl carbonate), and non-polar solvents (hexane, cyclohexane, toluene, diethyl ether, chloroform). However, it turned out that the solubility of CBP in most of them was approximately 1 μg/mL or less, with the following exceptions: DMSO (1.4 mg/mL), chloroform (similar to water), propylene glycol (15 μg/mL) and methanol (4 μg/mL). The fact that the solubility of CBP in organic solvents is generally even worse than in water unambiguously indicates that CBP is not a lipophilic substance. This result corresponds well with the calculated negative logP value as well as with rapid elution in reversed-phase HPLC, and may additionally serve as a proof for zwitterionic nature of CBP in solution [18].

Out of the solvents tested, DMSO turned out to be by far the best. Meanwhile, the extraordinary solubility of CBP in DMSO cannot be explained simply by the high dipole moment of this solvent (3.96 D for DMSO is similar to 3.92 D for acetonitrile and 3.82 D for DMF, but the latter two are poor solvents) or the polarizability (6.5 Å^3^ for DMSO is between the 5.2 Å^3^ for ethanol or 7.5 Å^3^ for THF). When it comes to the dielectric constant, DMSO has the highest value among the organic solvents tested (46.7), but, on the other hand, water, in which CBP is only sparingly soluble is characterized by an even higher one (78.5). Taking all this into account, it can be stated that the beneficial action of DMSO cannot be simply explained using such terms as dipole moment, polarizability, and dielectric constant. The exceptional capability of DMSO to solubilise CBP may result from the fact that a sulfoxide oxygen acts as a very strong hydrogen bond acceptor [19], which may solvate H-bond donors of CBP molecule, thereby reducing CBP-CBP intermolecular interactions and hence facilitating its solubilisation.

#### 2.2.2. Acid-Base Properties

The theoretical calculations described in our previous work [16] predicted that the ionisable functional groups of CBP include the carboxyl group and the secondary amino group in the pyrrolidine ring (N9′ according to atom labelling in Scheme 1). The current work presents two-step experimental verification of those findings. First, the total charge of CBP as a function of pH was investigated by capillary zone electrophoresis (CZE) using a low-pH background electrolytes (BGEs) in the range 1.8–3.3. The study was qualitative, not quantitative, thus serving evidence of the cationic nature of CBP at pH below 3. Then, the analysis of publicly available NMR spectra of CBP recorded in DMSO-d_6_ with and without the addition of trifluoroacetic acid allowed to indicate the amino group that undergoes protonation most likely. The combined results from CZE and NMR constitute experimental support for theoretical protonation states presented in [16].

**Figure 2:**
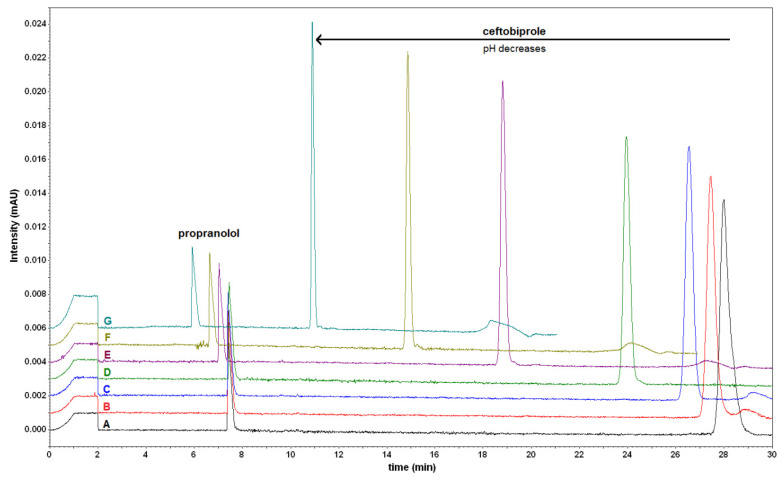
Electropherograms of an aqueous solution of CBP (0.8 mg/mL) and propranolol (0.5 mg/mL) recorded with BGEs at various pH values: (**A**) 3.3, (**B**) 3.1, (**C**) 2.9, (**D**) 2.5, (**E**) 2.3, (**F**) 2.1, and (**G**) 1.8.

The CZE experiments were performed with a series of BGEs prepared by adjusting 50 mM H_3_PO_4_ with NaOH to different pH values. It was assumed that at pH 3.3 the migration time of CBP is equal to the migration time of electroosmotic flow (EOF). When BGEs of lower pH are used, the silanol groups on the capillary walls remain largely uncharged, thus limiting the formation of the electrical double layer and substantially reducing the EOF. At pH values below 2.5, most silanol groups exist in their neutral form, resulting in a minimal zeta potential and negligible EOF flow, and consequently extremely long migration times of the EOF marker [20], of the order ≈300 min. This means that under negligible EOF conditions, the migration of an analyte toward the detector is primarily governed by its charge-to-mass ratio at low pH. Hence, the electrophoretic behaviour of CBP was assessed by reference to another compound, propranolol, which is established as a strong cation under these conditions (pK_a_ 9.5). Across the tested pH range (1.8–3.3), the migration time of propranolol, being a small ion (259.3 g/mol) with a +1 charge, was in the range 6–8 min. As the pH of the BGE decreased from 3.3 to 1.8, the migration time of CBP (534.6 g/mol) decreased significantly and continuously from 27 to 11 min, as shown in Figure 2, thus shortening its distance to propranolol. This unambiguously indicates that CBP carried a net positive charge at pH values below 3, which agrees well with the result of our previous computational study, predicting the presence of CBP in its cationic state at that pH range [16].

After confirming the cationic nature of CBP at pH values below 3, the next step was to experimentally determine the site of protonation. Out of the eight nitrogens in the CBP molecule, there are many N atoms not expected to undergo protonation for chemical reasons: those participating in amide bonds (N5, N5′, N1″), the oxime group (N10″), and those belonging to the aromatic thiadiazole ring (N5″, N8″). The remaining two represent the primary (N12″) and secondary (N9′) amino groups, and hence experimental data was needed to indicate the correct protonation site out of them. In this context, NMR spectra acquired only in DMSO-d_6_, rather than D_2_O, provided more conclusive evidence, as only DMSO allows the detection of signals also originating from the labile protons of the amino groups, which are undetectable in aqueous solvent. Table 2 cites the values of chemical shifts for heavily deshielded protons (δ > 7.5 ppm) from the available patent documents, thus comparing the results obtained in pure DMSO [10,11] with their counterparts recorded in DMSO acidified with deuterated trifluoroacetic acid (CF_3_COOD) [21]. This summary clearly indicates that the acidification of DMSO with use of CF_3_COOD caused the disappearance of the signal of one proton at 10.3 ppm and the appearance of the signal of two equivalent protons at 8.9 ppm. The observed change in the signal integral from one to two clearly illustrates the transition, occurring in DMSO solvent, from the uncharged secondary amino group –NH– to its protonated counterpart –NH_2_^+^–, while the noticed decrease in the chemical shift results from the increased shielding caused by the presence of an additional atom in the neighbourhood of the analyzed proton. On the other hand, if instead the primary amino group were the one that is protonated (a change from –NH_2_ to –NH_3_^+^), the NMR spectrum obtained in acidified DMSO would show the disappearance of a signal with integral two and the appearance of a signal with integral three, which, however, is not mentioned in the cited patents.

In the theoretical prediction of CBP protonation states, the pK_a_ value for carboxyl group was estimated as 3.00 by Chemicalize (from ChemAxon [22]) or 3.24 by Epik 7 (a part of the Schrödinger software package [23]), reflecting the fact a relatively low pH (well below 3) is required to reverse the dissociation of the carboxyl group. This is most likely due to the fact that this group is part of a system of four conjugated double bonds, spanning up to the C=O bond in the pyrrolidone ring (O=C−C4=C3−C1′=C2′−C6′=O, according to the atom labels shown in Scheme 1). The negative charge of the dissociated carboxylate (R–COO^−^) can be easily delocalized over the discussed long conjugated system, thus stabilizing the anion and making the attachment of H^+^ less favourable, and hence causing the pK_a_ value to be significantly low. The above mentioned impact of a conjugated bond system on acidity may be clearly demonstrated by comparing the pK_a_ values for two simple model carboxylic acids: 4.25 for acrylic acid (CH_2_=CH–COOH, manifesting the conjugation) and 4.87 for propionic acid (CH_3_–CH_2_–COOH, without conjugation). Meanwhile, the relationship between the aromaticity of the thiadiazole ring and the basicity of the attached primary amino group is analogous but opposite: the electron-withdrawing properties of the thaidiazole ring lead to the destabilization of the protonated amine and hence decrease the basicity of the discussed –NH_2_ group.

#### 2.2.3. Computational Studies of Intermolecular Interactions at Different pH

MD seems to be the technique of choice to study intermolecular interactions of CBP in different protonation states and investigate the formation of molecular aggregates (dimers, trimers, tetramers). Given the available computational power, MD simulations involved six randomly arranged cationic or zwitterionic CBP molecules in a periodic box of dimensions 140 × 140 × 140 Å, containing water molecules. This arrangement corresponded to a CBP concentration of 1.94 mg/mL, which is slightly lower than the solubility limit of CBP freeze-dried in 0.1 M HCOOH, 2.1 mg/mL [9].

In the case of zwitterionic CBP, a dimer formed 2.4 ns after starting the simulation. The radial distribution function (RDF), determined based on the centres of mass (COMs) of individual CBP species, was dominated by an intensive band in the COM-COM distance range of 4–6 Å, characteristic of the dimeric system. Surprisingly, CBP in its cationic state exhibited an even higher tendency to form multi-molecular agglomerates. A first dimer formed after 5.3 ns of simulation, and the other was created after 17.5 ns. Interestingly, in the simulation time range between 20.5 and 29.2 ns, these two aggregates joined together, thus forming a tetramer. Then, one cation left the agglomerate, thereby forming a trimer. Accordingly, the RDF curve obtained for cationic CBP turned out to be more complex than the analogous plot acquired for the zwitterionic species. The intensive peak, ranging from 3 to 5 Å and corresponding to the dimeric structure, was accompanied by a long tail, spanning up to 15 Å, and representing the tri- and tetrameric forms of CBP.

Given the fact that both the visualization of the obtained trajectories and the performed RDF analysis clearly demonstrated the great significance of dimeric structures, a more quantitative study was dedicated to characterize them in detail. Indicating the intermolecular interactions between individual CBP species in a dimer is vital to understand the observed limited solubility of this compound at neutral pH and significantly higher solubility in a highly acidic medium. If CBP-CBP attraction is greater than CBP-water interactions, an equilibrium state may be reached with a very limited aqueous solubility.

**Figure 3:**
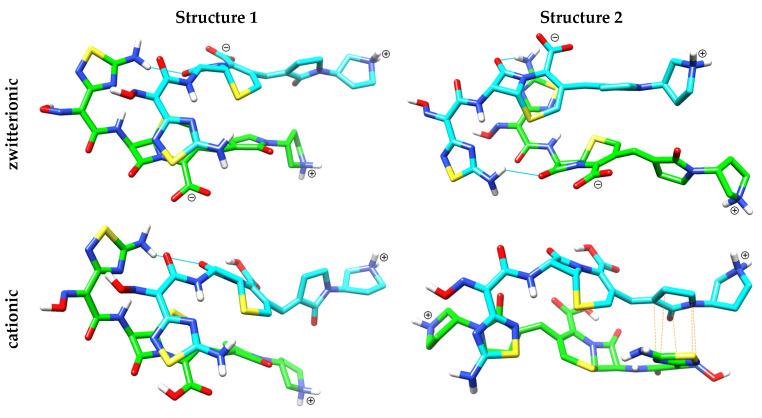
The most representatives structure of CBP dimers depending on the protonation state. Light blue continuous lines between atoms indicate hydrogen bonds. Dimeric structures were aligned so that the rings oriented parallel to each other are placed exactly one above the other (i.e., are overlaid). For Structure 2 of cationic CBP, the parallel rings forming the second pair are linked with dashed orange lines. These four strutures are available in the form of PDB files enclosed in Supplementary Material.

In order to check whether the dimeric structures (both for zwitterionic and cationic CBP) change in the course of the simulation, cluster analysis was performed based on calculations of root mean square-deviation (RMSD). The analysis resulted in obtaining a certain number of clusters, but, regardless of the protonation state, the majority of snapshots saved throughout the simulation belonged to the two largest clusters. For each of them, further denoted as “Structure 1” and “Structure 2”, it was possible to determine a central structure, i.e., a structure with the smallest RMSD in relation to all other snapshots belonging to the same cluster. The obtained molecular models of the dimeric structures are presented in Figure 3 as well as enclosed in the electronic form of PDB files in the Supplementary Material to facilitate their visual inspection.

Given that each CBP molecule includes several heterocyclic rings, the intermolecular forces between them may involve π-π or stacking interactions. For this reason, a detailed geometric analysis of their mutual arrangement was conducted for each cluster independently, thus allowing us to obtain the averaged perpendicular intermolecular distances ($d_{⊥}$) of the ring planes, lateral displacements ($d_{∥}$) of the geometrical centres of the rings, as well as the angular tilts between the ring planes (Table 3). Obtaining stable values for $d_{⊥}$, less than 4 Å, accompanied by small $d_{∥}$ values and tilt angles, supports the hypothesis of the existence of π-π or stacking interactions. Meanwhile, the presence of many hydrogen bond donors and acceptors in each CBP molecule strongly indicates that the intermolecular interactions between them may include hydrogen bonding. Hence, for each cluster, a quantitative analysis of hydrogen bonding was performed. A geometric criterion was verified: a hydrogen bond was detected if the donor-acceptor distance was not greater than 3.5 Å and the hydrogen-donor-acceptor angle was not higher than 30^°^. The hydrogen bond occupancy was determined as a percentage of the snapshots in which a specified hydrogen bond was detected relative to all snapshots forming a cluster. However, it should be emphasized that obtaining both high values for standard deviations of geometric measures as well as fractional values of hydrogen bond occupancies results directly from the dynamic character of the simulations. The atoms are still in motion, thus leading to continuous minor conformational changes in the molecules, which results in sequential breaking and recreating the intermolecular interactions.

Regarding zwitterionic CBP, a dimer with geometry of Structure 1 (as shown in Figure 3) was present between 7 and 16 ns of the simulation. Unexpectedly, the two CBP molecules aligned parallel to each other with pyrrolidine rings facing the same direction despite the repulsive interaction between the two positively charged protonated secondary amino groups (N9′). Meanwhile, significant intermolecular hydrogen bonds were detected between the −NH_2_ group (N12″) attached to the thiadiazole ring and both the carbonyl oxygen (O9″) in C7 substituent (occupancy: 18%) as well as carbonyl oxygen (O9) in the β-lactam ring (12%). Furthermore, the planes of the thiadiazole and dihydrothiazine rings of two interacting CBP molecules were placed approximately parallel to each other and distanced at 3.95 Å, which suggests the presence of favourable π-π or stacking interactions. Subsequently, after approximately 16 ns from the beginning of the simulation, a rearrangement to Structure 2 occurred. As can be seen in Figure 3, the CB molecule shown in blue moved relative to the molecule shown in green, which had a similar effect as if they swapped places with each other. The quantitative hydrogen bond analysis revealed the important role of the N12″-O9″ interaction (occupancy of 19%) as well as the pair of mutual N12″-O9 interactions (16% and 9%, respectively). The thiadiazole and dihydrothiazine rings of corresponding CBP molecules were positioned one above the other at a similar distance as previously, enabling their attractive interactions.

In the case of the dimer formed by two CBP cations, the cluster analysis again allowed to propose two most representative structures. Initially (6–10 ns of simulation, Structure 1), a dimer was created with an arrangement very similar to the dimer initially formed by zwitterionic CBP. The thiadiazole and dihydrothiazine rings of two interacting cations were distanced at ca. 3.92 Å, while the occupancies of the detected hydrogen bonds reached 34% for N12″-O9″ and 11% for N12″-O9. However, around 10 ns, a rearrangement occurred, resulting in the cations aligning in opposite directions (Structure 2). This allowed for greater spatial separation of the two positively charged secondary amino groups, thus weakening their repulsive interaction. The new arrangement prevented the formation of any intermolecular hydrogen bond between NH_2_ and CO, as these groups became spatially separated. Furthermore, the altered dimer geometry did not allow for parallel arrangement of the thiadiazole and dihydrothiazine rings of the interacting cations, but Structure 2 revealed an interesting alignment of the five-member rings. Namely, the thiadiazole ring of each cation was aligned almost parallel to the pirrolidone ring of the other, both distanced at 3.72 Å, which indicates the possibility of stacking or π-π interactions.

The performed MD analysis clearly demonstrated that changing the pH (and thus the dominant protonation state) caused a significant difference in the geometric arrangement of the formed aggregates and hence altered the interaction pattern between individual species. In the case of zwitterionic CBP, the presence of strong CBP-CBP hydrogen bonds was detected, which may be thermodynamically preferred relative to CBP-water interactions. On the other hand, it may be hypothesized that the interaction of CBP hydrogen bond donor (N12″) with sulfoxide oxygen of DMSO is even stronger than the CBP-CBP hydrogen bonds discussed above, which may be the reason for exceptionally high solubility of ceftobiprole in DMSO. Meanwhile, in the case of cationic CBP, only weak stacking interactions could be considered, which seem to be easier to be overcome through interactions with water molecules from the solvent. Furthermore, as more and more cations approach each other, attempting to form an aggregate, its net charge steadily increases, thereby preventing further agglomeration. This may serve as a mechanistic explanation for the enhanced solubility of CBP in a strongly acidic environment.

### 2.3. Biological Properties of a CBP-CD Drug Delivery System

The limited solubility of the active form of CBP prompted us to develop a new drug delivery system with improved physicochemical properties. Preliminary 18 MD simulations of inclusion complexes formed between various CDs and CBP in different protonation states demonstrated that the most thermodynamically favourable conditions involved the cationic form of CBP (corresponding to pH well below 3) and anionic SBE-β-CD [16]. Based on these findings, we conducted a series of experiments in which newly developed drug delivery systems consisting of CBP, SBE-β-CD, and a selected acid were evaluated in terms of CBP solubility and physical and chemical stability [9]. Among the tested formulations, the most promising system was obtained by freeze-drying a solution containing CBP, MA, and SBE-β-CD at a molar ratio of 1:25:4. This system outperformed the analogous CBP/MA/SBE-β-CD formulations prepared at other molar ratios (1:5:4, 1:15:4, and 1:20:4) with respect to CBP solubility. Our previous article [9] focused on physicochemical analyses, whereas the present paper discusses the biological properties of the most promising CBP delivery system.

#### 2.3.1. Antibacterial Activity

Hospital-acquired pneumonia is primarily caused by certain bacterial species: *S. aureus*, including MRSA strains, *Streptococcus pneumoniae*, and less frequently by *Klebsiella pneumoniae*, *Escherichia coli*, *Acinetobacter baumannii*, *Pseudomonas aeruginosa*, and *Stenotrophomonas maltophilia*. Considering these common pathogens, the activity of the new CBP/MA/SBE-β-CD system was investigated against them. CBP-M was used as the reference compound. According to the procedure described in [24], the broth microdilution method was employed to determine the minimal inhibitory concentration (MIC), and the minimal bactericidal concentration (MBC) values. The antibacterial activity of the CBP/MA/SBE-β-CD system was shown to be due to the activity of CBP alone. The other two components of the system, MA and SBE-β-CD, were inactive against all tested strains (MIC values > 32 mg/L for MA and >200 mg/L for SBE-β-CD). The new CBP/MA/SBE-β-CD system, similarly to CBP-M and CBP, exhibited high activity against *S. pneumoniae* strains (in the MIC range 0.0078–0.0625 mg/L) and was also active against methicillin-sensitive *S. aureus* (MSSA) and methicillin-sensitive *Staphylococcus epidermidis* (MSSE) (MIC 0.25–0.5 mg/L, Table 4). According to the recommendation of the European Committee on Antimicrobial Susceptibility Testing (EUCAST) from 2025 [25], the CBP cut-off point defining susceptibility of *S. aureus*, including MRSA, is ≤2 mg/L. In the case of MRSA, for 4 of the 6 strains tested, borderline susceptibility to CBP, CBP-M and CBP/MA/SBE-β-CD was observed (MIC 1–2 mg/L).

The 2- to 4-fold higher activity of the CBP/MA/SBE-β-CD system and CBP compared with CBP-M against the majority of Gram-negative rods (Table 5), in terms of the MIC and MBC values, was revealed. However, only *E. coli* and *K. pneumoniae* strains lacking acquired β-lactamase genes were susceptible to CBP/MA/SBE-β-CD, CBP and CBP-M. The CBP cut-off value defining susceptibility of *Enterobacterales* is ≤0.25 mg/L [25]. In the case of the isolates producing CMY-2 cephalosporinases, *E. coli* remained susceptible, while *K. pneumoniae* showed resistance to all tested compounds. According to the summary of product characteristics (SmPC) [26], CBP-M is inactive against *Enterobacterales* strains overexpressing genes encoding cephalosporinases such as *K. pneumoniae* MUW 78 CMY-2 (+).

#### 2.3.2. Cytotoxicity Study

Beyond confirming the microbiological efficacy of new antibiotics or their delivery systems, it is essential to evaluate their cytotoxicity in human cells and to compare the concentrations producing a 50% inhibitory effect, expressed as the selectivity index (SI) [27]. Cytotoxicity assays were performed using human lung adenocarcinoma (A549), ovarian cancer (SKOV-3), and normal lung fibroblast (CCD-8Lu) cell lines [28,29]. The MTT assay was employed to assess the cytotoxicity of the CBP/MA/SBE-β-CD system, CBP, and its prodrug, CBP-M. In addition, the cytotoxicity of MA and SBE-β-CD at concentrations corresponding to those present in the delivery system was examined.

**Figure 4:**
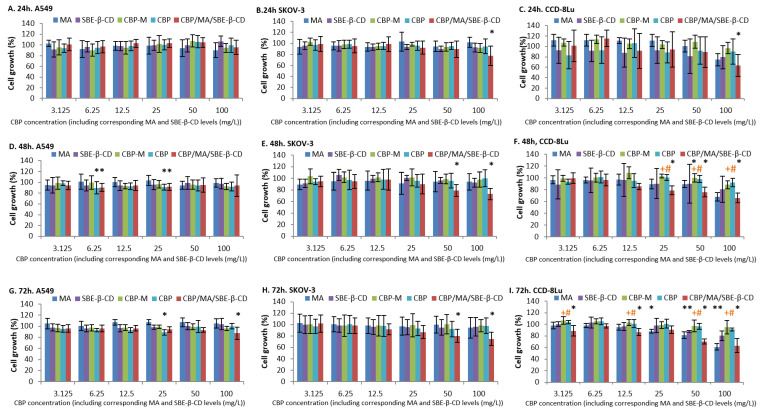
Changes in cell growth after treatment with MA, SBE-β-CD, CBP-M, CBP, and the CBP/MA/SBE-β-CD system, versus control, in A549 cells (**A**,**D**,**G**), SKOV-3 cells (**B**,**E**,**H**), and CCD-8Lu cells (**C**,**F**,**I**) after 24 h (**A**–**C**), 48 h (**D**–**F**), and 72 h (**G**–**I**) incubation. The bars represent the mean ± S.D., * *p* value < 0.05, difference from control (Dunnett’s test); + *p* value < 0.05, difference between CBP-M and CBP/MA/SBE-β-CD system (Tukey’s test) and; # *p* value < 0.05, difference between CBP and CBP/MA/SBE-β-CD system (Tukey’s test).

The results of these assays are shown in Figure 4. Both the CBP/MA/SBE-β-CD system and the reference compounds exhibited low cytotoxicity in A549 cells (Figure 4A,D,G). More pronounced effects were observed in SKOV-3 cells after treatment with the CBP/MA/SBE-β-CD system at 50 mg/L and 100 mg/L for 48 and 72 h, resulting in an approximately 25% decrease in cell growth compared with the control (Figure 4B,E,H). CBP-M, MA, and SBE-β-CD did not display significant cytotoxicity in SKOV-3 cells within the tested concentration ranges. Moreover, the cytotoxicity of CBP and the CBP/MA/SBE-β-CD system was not statistically different, whereas statistically significant differences in cell growth between CBP-M and the CBP/MA/SBE-β-CD system were observed only at the highest concentrations, reaching a maximum of approximately 16%.

In normal CCD-8Lu fibroblasts, MA at the two highest concentrations led to reduced cell growth after 48 and 72 h, and the magnitude of this effect was comparable to that observed for the CBP/MA/SBE-β-CD system. At 50 mg/L and 100 mg/L, both MA and the CBP/MA/SBE-β-CD system decreased cell growth by approximately 30–40%. These findings indicate that MA contributes to the overall cytotoxic response of the formulation. Although this effect is undesirable, it occurred only at concentrations markedly exceeding those required for antibacterial activity. Consistent with the contribution of MA to the system’s cytotoxicity, the CBP/MA/SBE-β-CD formulation showed higher cytotoxicity than CBP or CBP-M at the highest concentrations tested: the difference relative to CBP did not exceed 26%, whereas compared with CBP-M, the system reduced cell growth by approximately 33% at 100 mg/L and by 20% at 50 mg/L.

The MTT assay results showed that the 50% inhibitory concentration (IC_50_) could not be determined, as it exceeded 100 mg/L, substantially higher than the MIC values. Based on the highest MIC of 4 mg/L for the CBP/MA/SBE-β-CD system, the selectivity index (SI) was estimated to exceed 24, indicating selective antimicrobial activity and a favourable safety profile [27,30].

To obtain qualitative insights into the effects of the tested compounds on cell and population morphology, morphological observations were performed [31].

**Figure 5:**
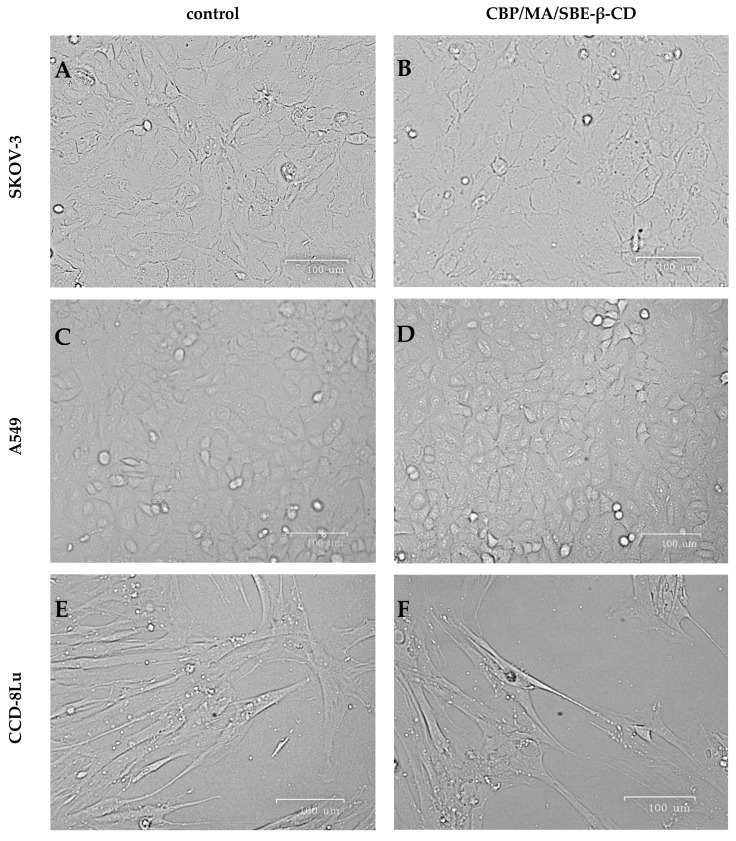
Morphology of SKOV-3 (**A**,**B**), A549 (**C**,**D**) and CCD-8Lu (**E**,**F**) cells after 72 h of incubation with the CBP/MA/SBE-β-CD system. Control cells: (**A**,**C**,**E**); cells treated with CBP/MA/SBE-β-CD: (**B**,**D**,**F**). Scale bar = 100 µm.

As shown in Figure 5, a 72 h treatment with the highest tested concentration (100 mg/L) of the CBP/MA/SBE-β-CD system did not alter the morphology of any of the tested cell lines. However, in the normal CCD-8Lu cell line, the number of cells was reduced compared with the control. These findings are consistent with the MTT assay results.

## 3. Materials and Methods

### 3.1. Materials

CBP was purchased from BenchChem (Austin, TX, USA).

The deionised water was obtained from a Direct-Q 3 UV Millipore system (Merck, Darmstadt, Germany).

In the solubility studies, the following organic solvents of HPLC or analytical grade were used: methanol (Sigma-Aldrich, St. Louis, MO, USA), anhydrous ethanol (POCH, Gliwice, Poland), *n*-butanol (Sigma-Aldrich, St. Louis, MO, USA), propylene glycol (Chempur, Piekary Śląskie, Poland), DMSO (Chempur, Piekary Śląskie, Poland), acetonitrile (Honeywell, Seelze, Germany), DMF (Honeywell, Seelze, Germany), acetone (Sigma-Aldrich, St. Louis, MO, USA), ethyl acetate (POCH, Gliwice, Poland), THF (Sigma-Aldrich, St. Louis, MO, USA), dichloromethane (Sigma-Aldrich, St. Louis, MO, USA), dioxane (Sigma-Aldrich, St. Louis, MO, USA), dimethyl carbonate (Sigma-Aldrich, St. Louis, MO, USA).

In CZE studies, BGEs were prepared using phosphoric acid (for HPLC, 85%, Honeywell, Seelze, Germany) and sodium hydroxide (Avantor Performance Materials Poland, Gliwice, Poland). The prepared solutions of 50 mM H_3_PO_4_ as well as 5 M, 1 M and 0.1 M NaOH were filtered using 0.45 μm nylon filter. Appropriate volumes of 5 M NaOH were added to 50 mM H_3_PO_4_ to obtain buffers of the following pH values: 2.1, 2.3, 2.5, 2.9, 3.1 and 3.3. Propranolol hydrochloride (United States Pharmacopeial Convention, Rockville, MD, USA) was used as a reference compound for comparison with the tested substance. The analyzed sample solution for CZE studies contained 0.8 mg/mL CBP and 0.5 mg/mL propranolol.

Sample preparation using the freeze-drying technique (CBP freeze-dried in 0.1 M HCOOH and CBP/MA/SBE-β-CD in a 1:25:4 ratio) was described in detail in our previous paper [9].

Direct antimicrobial activity was determined against the following standard strains: (1) Gram-positive cocci: methicillin-sensitive *Staphylococcus aureus* ATCC 29213 (MSSA), methicillin-sensitive *Staphylococcus epidermidis* ATCC 12228 (MSSE), methicillin-resistant *S. aureus* subsp. *aureus* ATCC 43300 (MRSA), five clinical MRSA strains (*S. aureus* 664, *S. aureus* 1576, *S. aureus* 1712, *S. aureus* 1991, and *S. aureus* 2541), *Streptococcus pneumoniae* ATCC 49619, three clinical *S. pneumoniae* strains (*S. pneumoniae* MUW 2/299o21, *S. pneumoniae* MUW 3/39085, and *S. pneumoniae* MUW 4/cw); (2) Gram-negative bacteria from *Enterobacteriales* order: *Escherichia coli* ATCC 25922, *E. coli* NCTC 8196, *E. coli* NCTC 10538, *E. coli* MUW 77 CMY-2(+), *Klebsiella pneumoniae* ATCC 13883, *K. pneumoniae* MUW 78 CMY-2(+); (3) Gram-negative non-fermentative rods: *Acinetobacter baumannii* ATCC 19606, and *Pseudomonas aeruginosa* ATCC 27853. Among the strains listed above, four of them (*S. aureus* ATCC 29213 MSSA, *S. pneumoniae* ATCC 49619, *E. coli* ATCC 25922, and *P. aeruginosa* ATCC 27853) were simultaneously used as quality control strains for determining antibacterial activity according to CLSI recommendations [32]. All strains were stored at −80 °C. Prior to testing, each bacterial strain, except *S. pneumoniae* was subcultured twice on tryptic soy agar (TSA; bioMérieux, Marcy l’Etoile, France) for 24 h at 37 °C. *S. pneumoniae* strains were subcultured twice on Columbia agar with sheep blood (bioMérieux, Marcy l’Etoile, France) for 48 h at 37 °C in an atmosphere containing 5% CO_2_. Cation-adjusted Mueller-Hinton broth with 5% lysed horse blood and 20 mg/L β-NAD (MH-F broth; Liofilchem srl, Roseto degli Abruzzi, Italy) and cation-adjusted Mueller-Hinton broth (Becton, Dickinson and Company, Franklin Lakes, NJ, USA) were used as culture media.

The human lung adenocarcinoma cell line (A549), ovarian carcinoma cell line (SKOV-3), and normal lung fibroblasts (CCD-8Lu) were obtained from the American Type Culture Collection (ATCC, Manassas, VA, USA). The cells were cultured in F-12K medium with L-glutamine (Gibco, Thermo Fisher Scientific, Waltham, MA, USA), McCoy’s 5A medium with L-glutamine (Biowest, Nuaillé, France), and DMEM/F-12K medium with L-glutamine (Biowest, Nuaillé, France) for A549, SKOV-3, and CCD-8Lu cells, respectively. All culture media were supplemented with 10% (*v*/*v*) heat-inactivated fetal bovine serum (FBS; Gibco, Thermo Fisher Scientific, Waltham, MA, USA) and 1% (*v*/*v*) antibiotic–antimycotic solution (Sigma-Aldrich, St. Louis, MO, USA).

3-(4,5-Dimethylthiazol-2-yl)-2,5-diphenyltetrazolium bromide (MTT) was purchased from Sigma-Aldrich (Sigma-Aldrich, St. Louis, MO, USA).

### 3.2. HPLC Studies

In each experiment, 1 mL of a solvent was added to 1 mg of CBP, and the resultant suspension was sonicated for 15 min, mechanically shaken at 1500 rpm for 15 min and centrifuged at 14,800 rpm for 15 min. In the case of DMSO, the volume of solvent added was decreased to 250 μL to allow for obtaining higher concentrations. Then, clear supernatants were transferred to clean HPLC vials and the determination of CBP in each sample was performed using the HPLC method developed in [15] with an injection volume of 1 μL.

### 3.3. CZE Studies

A Beckman PA 800 plus Pharmaceutical Analysis System from Beckman Coulter, Inc. (Fullerton, CA, USA) equipped with a photodiode array detector and a liquid-cooling device was used. Uncoated fused-silica capillaries from Polymicro Technologies (Kehl, Germany) with a total length of 49 cm (an effective length of 39 cm) and  internal diameter of 75 μm were used throughout the analysis. They were operated at an applied voltage of 18 kV with a ramp time of 1 min (the operating current did not exceed 120 μA) in normal polarity mode (detected at the cathode) and an operating temperature of 22 °C. Samples were hydrodynamically injected with a pressure of 0.5 psi for 3 s. The detection wavelength was 320 nm corresponding to the absorption maximum of CBP. The electropherograms were recorded using a 32 Karat (Scientific Software, 32 Karat^TM^ Version 9.1 and PA 800 plus Software Version 1.1 from Beckman Coulter, Inc., Fullerton, CA, USA) chromatographic data system.

New capillaries were pre-conditioned by performing a 3 min high pressure (20 psi) rinse with 1 M NaOH followed by 0.1 M NaOH and deoinised water. Between consecutive injections, the capillary was conditioned by washing with 0.1 M NaOH (20 psi, 0.5 min) followed by deionised water (20 psi for 0.5 min) and a proper BGE (20 psi, 2 min).

### 3.4. NMR Studies

All ^1^H and correlation NMR experiments were recorded on a Varian vnmrs 600 MHz spectrometer (Agilent Technologies, Santa Clara, CA, USA) using an Auto XID (inverse configuration) probehead, with resonance frequencies of 599.8 MHz for ^1^H and 150.8 MHz for ^13^C measurements. All spectra were run in D_2_O solutions at room temperature (25 °C). The ^1^H NMR spectra and the ^1^H dimension in two-dimensional (2D) heteronuclear spectra were referenced to the solvent (D_2_O, δ_H_ = 4.64 ppm). For both acquisition and processing of data, the standard parameters and procedures were applied. The duration of the 90° pulse for ^1^H was between 6.75 and 6.90 μs. The spectral width for proton spectra was set to 5000 Hz. The relaxation delay time in the experiments was set to 1 s, unless stated otherwise.

The 1D ^1^H NMR spectra were obtained using a pulse duration of 2.5 μs, which corresponds to the tilt angle approximately 32.6°. In each single experiment, 32 scans were recorded, each with 16,384 complex data points. The assignment of signals to individual atoms was based on the following correlation 2D spectra: Correlation Spectroscopy (COSY), Rotating-frame Overhauser Enhancement Spectroscopy (ROESY), Heteronuclear Single-Quantum Correlation (HSQC) and Heteronuclear Multiple-Bond Correlation (HMBC). The ^1^H-^1^H couplings through chemical bonds were determined using the gCOSY (gradient selected COSY) pulse sequence with 256 increments applied for t_1_, each comprising 2 scans with 1024 complex points. Meanwhile, the ^1^H-^1^H couplings through space were determined using the ROESY pulse sequence with 200 increments applied for t_1_, each comprising 4 scans with 1024 complex points and 300 ms spinlock mixing time. Data processing was carried out using the OpenVnmrJ 3.1A software [33].

The coupling between ^1^H and ^13^C nuclei through one bond was determined using the gHSQCAD sequence with a spectral width of 5000 Hz in F2 and 19,607.8 Hz in F1 and the ^1^J(C,H) coupling constant of 146 Hz. 400 increments were applied for t_1_, each comprising 2 scans with 1024 complex points. The long-range coupling between ^1^H and ^13^C nuclei through more than one bond was determined using the gHMCAD sequence with a spectral width of 5000 Hz in F2 and 27,146.3 Hz in F1 and with an ^n^J(C,H) coupling constant of 8 Hz. A total of 256 t_1_ increments were collected, each consisting of 64 scans with 1500 complex points.

Meanwhile, due to the inherently low sensitivity of the 1D ^13^C spectrum, it was recorded using different instrumentation. Specifically, a Varian vnmrs 500 MHz spectrometer equipped with an AutoXDB probe (direct configuration) probehead operating at a resonance frequency of 125.7 MHz for ^13^C. Spectra were acquired using a 3.13 μs pulse (corresponding to a tilt angle of 30°), a spectral width of 37,868.8 Hz, and a relaxation delay of 0.5 s. A total of 52,000 scans were collected, each containing 45,455 complex data points.

### 3.5. ATR-FTIR Studies

A Nicolet iS5 FT-IR spectrometer (Thermo Scientific, Waltham, MA, USA) with an attenuated total reflectance (ATR) attachment with diamond crystal was used to collect spectra in the region from 4000 to 400 cm^−1^. Spectra were recorded using 32 scans with a resolution of 4 cm^−1^. Data were collected using OMNIC 9.8.

### 3.6. DFT Calculations

DFT calculations were performed using the Gaussian 09 software package [34]. Ninety-six CBP structures were manually constructed and minimized using density functional theory (DFT) and geometry optimizations with the Becke, three-parameter, Lee-Yang-Parr B3LYP [35] exchange-correlation function with a basis set of 6-31G(d) [36]. Forty of these structures were then selected based on their lowest energy, and geometry optimized at the B3LYP/6-311+G(d,p) [37] level with IEFPCM [38] (polarizable continuum model) for water solvent effects simulation (SCRF theory). No imaginary frequencies were obtained for the optimized geometries, and thus all the optimized structures represented true minima on the potential energy surface. The chemical shielding calculations were conducted with the gauge including atomic orbital (GIAO) method [39] using the same level as for geometry optimization. The resulting NMR shieldings were averaged for structures arranged according to increasing energy. The NMR chemical shifts were calculated by a linear fit of shieldings to the experimental data. The optimal fit to the experimental data was obtained when fifteen CBP structures were averaged (R^2^ = 0.9989).

### 3.7. MD Simulations

#### 3.7.1. Performing the Simulation

The procedure of performing the MD simulations was similar to that described in [16] with numerous adjustments. The structure of the CBP molecule was obtained from PubChem (CID 135413542, [40]), and the structures of the individual protonation states (cationic, zwitterionic) were created using Avogadro 1.2.0 [41]. These species of CBP were then parametrised with the use of General AMBER Force Field (GAFF) by means of the following parts of AmberTools 20 suite [42]: antechamber [43,44] to calculate partial charges for each atom on the AM1/BCC level of theory, parmchk2 to obtain the missing parameters of GAFF, and LEaP to generate AMBER topologies. Then, AMBER topology files were converted to GROMACS files with the use of acpype 2022.6.6 script [45].

Two MD simulations were performed in GROMACS 2022.5 [46]: one for cationic and the other for the zwitterionic CBP. Topology files were created manually based on the results obtained with acpype. Cubic periodic boxes of dimensions 14 × 14 × 14 nm were created and six residues of CBP (either cationic or zwitterionic) were randomly inserted into newly created boxes. The boxes were filled with TIP3P water molecules (89,562 for the protonated CBP and 89,559 in the case of zwitterionic one). To neutralize the charge of the system with protonated CBP, six Cl^−^ anions were added. Potential energy of the systems was minimized using the steepest decent method. Afterwards, 100 ps of equilibration in NVT ensemble and 100 ps in NPT ensemble were performed. Position restrains were put on non-hydrogen atoms of the solute only during the equilibration.

Then, 35 ns of production run was performed with the set of parameters chosen based on the GROMACS tutorial [47] and parameters proposed by acpype, listed below, similar to our previous study [16]. The equations of motion were solved using leap-frog integrator with a time step of 2 fs, and with the use of LINCS holonomic constraint algorithm applied for bonds to hydrogen atoms. A Verlet list of short-range neighbours was used. For van der Waals forces, a switching function at distances between 0.9 and 1.1 nm was applied (according to the output of acpype). The Particle Mesh Ewald (PME) method was used to calculate long-range electrostatic forces. Solute and solvent were coupled to two separate V-rescale thermostats at 298.15 K. Pressure of the system (1 bar) was controlled by Parrinello-Rahman barostat. Snapshots of the system were saved in 10 ps intervals.

#### 3.7.2. Trajectory Post-Processing

The cluster analysis was performed on the obtained trajectories using the *gmx rms* followed by *gmx cluster* command with a cut-off of 0.12 nm, disregarding first 6 ns of simulation (the time needed to form a stable dimer).

Geometric statistics describing the arrangement of pairs of interacting rings were calculated using the MDAnalysis 2.9.0 [48,49] and NumPy 2.3.0 [50] Python packages as well as an in-house Python 3.10 script. For each ring, only heavy atoms forming that ring (without the attached substituents) were considered in the process of fitting the optimal ring plane. Using these atoms, a geometrical centre c(t) of each ring was calculated, then the positions of individual atoms $r_{i}(t)$ were referenced to a corresponding ring centre: $x_{i}(t)=r_{i}(t)-c(t$), and arranged into a matrix X, consisting of $x_{i}$ as rows. Singular value decomposition (SVD) of X was performed, and the right-singular vector corresponding to the smallest singular value was interpreted as the normal vector v(t) to the best-fit ring plane. Afterwards, for each pair of the ring planes to be analyzed, the unit normal vectors $n_{1}(t)$ and $n_{2}(t)$ were determined by means of normalization of $v_{1}(t)$ and $v_{2}\left(t\right)$, respectively.

For each frame, the vector connecting the two centroids was defined as $d(t)=c_{2}(t)-c_{1}\left(t\right)$. To obtain a symmetric measure of the stacking distance (perpendicular separation of the ring planes), an average normal vector $n_{avg}\left(t\right)$ was constructed:

(1) $$n_{avg}(t)=\frac{n_{1}\left(t\right)+n_{2}\left(t\right)}{\left|n_{1}\left(t\right)+n_{2}\left(t\right)\right|}$$

The perpendicular inter-planar distance $d_{⊥}\left(t\right)$ was determined as the projection of the inter-centroid vector onto this average normal (as the absolute value of the scalar product):

(2) $$d_{⊥}(t)=|d(t)\cdot n_{avg}(t)|$$

The lateral displacement $d_{∥}$ between the two ring centres (describing how far the corresponding centres are shifted from each other within the ring plane) was calculated according to the Pythagoras’ theorem:

(3) $$d_{∥}\left(t\right)=\sqrt{d^{2}\left(t\right)-d_{⊥}^{2}\left(t\right)}$$

The relative orientation of the two rings was quantified by the tilt angle, defined as an angle between their normal vectors, $n_{1}(t)$ and $n_{2}(t)$:

(4) $$\theta (t)=\arccos(|n_{1}(t)\cdot n_{2}(t)|)\;$$

Geometrical analysis was performed for each interacting pair of rings from an individual cluster independently. The means and standard deviations were calculated using only those simulation snapshots in which the interactions may have taken place, i.e., the following conditions were fulfilled: $d_{⊥}<5\;Å$, $d_{∥}<4\;Å$ and tilt angle <50°.

### 3.8. Antimicrobial Activity Studies

The antimicrobial activity was evaluated as previously described [24], using double agent dilutions method, according to the recommendations of the Clinical and Laboratory Standards Institute (CLSI) [51,52]. The determination of the MIC values for *S. pneumoniae* strains was performed in Cation-adjusted Mueller-Hinton broth with 5% lysed horse blood and 20 mg/L β-NAD. For the other strains, the MIC values were determined in Cation-adjusted Mueller-Hinton broth. The tested agents were dissolved in water. Due to the limited solubility of CBP in water, the MIC values of CBP, CBP-M, and CBP/MA/SBE-β-CD were determined in the concentration range up to 8 mg/L. However, MA and SBE-β-CD agents were tested at concentrations resulting from the molar ratio of CBP/MA/SBE-β-CD (1:25:4) system. The MIC values of the tested compounds were determined in three independent replicates.

### 3.9. Cytotoxicity Studies

#### 3.9.1. MTT Assay

Cytotoxicity was evaluated using the 3-(4,5-dimethylthiazol-2-yl)-2,5-diphenyltetrazolium bromide (MTT) assay. Cells in the logarithmic growth phase were seeded into 96-well plates at the following densities: A549—1.0 × 10^5^ cells/mL, SKOV-3—1.3 × 10^5^ cells/mL, and CCD-8Lu—1.4 × 10^5^ cells/mL. After 24 h of incubation, the cells were treated with CBP, MA, SBE-β-CD, CBP-M, or the CBP/MA/SBE-β-CD system. All compounds were dissolved in 10% DMSO in water.

The concentrations of CBP, CBP-M, and the cyclodextrin-based formulation were normalized to the CBP content to ensure comparability between treatments. Solutions containing equivalent CBP concentrations were directly compared. The concentrations of MA and SBE-β-CD corresponded to their respective proportions present in the cyclodextrin-based formulation.

After 24, 48, and 72 h of incubation, the cells were washed with PBS and incubated with 0.5 mg/mL MTT solution for 3 h. The resulting formazan crystals were dissolved in isopropanol, and absorbance was measured at 570 nm using a PowerWave X microplate spectrophotometer (BioTek Instruments, Winooski, VT, USA). All experiments were performed in triplicate.

Cell growth was calculated according to the following equation:

% *cell growth = (absorbance of treated cells/absorbance of control cells)* × 100%

#### 3.9.2. Statistical Analysis

Data are presented as mean ± standard deviation. All experiments were conducted in a minimum of three independent biological replicates. Statistical analysis was performed using one-way analysis of variance (ANOVA) followed by Dunnett’s post hoc test for pairwise comparisons with the untreated control the post hoc Tukey’s test was used to compare pairs of group means (*p* < 0.05). All statistical analyses were performed using GraphPad Prism 7.05 (GraphPad Software, San Diego, CA, USA).

#### 3.9.3. Morphology Studies

The cells were seeded and prepared as described in Section 3.9.1. To assess the effect of the cyclodextrin-based CBP delivery system (100 µg/mL) on cell morphology after 72 h of incubation, microscopic observations were performed using a Zoe™ Fluorescent Cell Imager (Bio-Rad Laboratories, Hercules, CA, USA) equipped with a 40× objective lens. The morphology of cells treated with the cyclodextrin-based CBP system was compared with that of the untreated control.

## 4. Conclusions

In summary, this study provides a comprehensive insight into the structural and physicochemical characteristics of CBP in its native form. For the first time, a complete interpretation of its NMR spectra in D_2_O (^1^H, ^13^C, COSY, HSQC, HMBC) has been achieved, supported by DFT calculations to resolve ambiguities in the ^13^C assignments. The findings from CZE and NMR studies confirm that the cationic species of CBP, with a protonated secondary amino group, prevails at pH well below 3, whereas the zwitterionic species, with both a protonated secondary amino group and a dissociated carboxyl group dominates at higher pH values.

Computational results indicate a strong tendency of CBP molecules to form dimers or multimers through hydrogen bonding or π-π interactions, depending on their protonation state. In the case of protonated CBP, the proximity of two cations is stabilized by relatively weak stacking or π-π interactions, and the aggregate size is limited because the overall positive charge increases with each additional cation. In contrast, zwitterionic CBP molecules associate through stronger hydrogen bonds, leading to the formation of more stable multimers that resist dissociation into monomers, which, in turn, restricts its solubilization.

To overcome these intrinsic solubility issues, a novel freeze-dried cyclodextrin-based formulation (CBP/MA/SBE-β-CD, 1:25:4 molar ratio) was developed. This system markedly enhances the solubility of CBP while retaining its antibacterial activity, which originates solely from the CBP component. The CBP/MA/SBE-β-CD formulation exhibits strong activity against *S. pneumoniae* and staphylococci, with borderline susceptibility observed for MRSA strains. The 2- to 4-fold higher activity of the CBP/MA/SBE-β-CD system and CBP compared with CBP-M against majority Gram-negative rods was revealed.

The CBP/MA/SBE-β-CD system demonstrated selective antimicrobial activity with minimal cytotoxic effects in human cell lines. The absence of significant morphological alterations and the high selectivity index (>24) indicate a favourable safety profile and good biocompatibility. These results suggest that the CBP/MA/SBE-β-CD system may serve as a promising and safe antibiotic delivery platform.

Overall, the CBP/MA/SBE-β-CD system represents a promising alternative formulation of CBP, offering improved solubility and enhanced activity against selected Gram-negative pathogens, while maintaining stable activity against Gram-positive strains. The present findings expand our understanding of ceftobiprole’s structural behaviour, intermolecular interactions, and formulation potential, providing a solid foundation for further analytical and pharmaceutical research on this antibiotic.

## Figures and Tables

**Table 1:** The ^1^H and ^13^C chemical shifts δ for CBP in water.

Atom		NMR Chemical Shift (ppm)	
	^1^H (exp.)	^13^C (exp.) ^(a)^	^13^C (calc.) ^(b)^
2	3.69; 3.63	25.7	27.1
3	-	**116.2**	114.7
4	-	**133.6**	142.0
6	5.14	57.4	60.0
7	5.77	58.9	55.6
8	-	163.7	162.8
10	-	**168.9**	166.4
1′	6.91	127.2	130.9
2′	-	132.1	131.2
3′	2.87	23.7	21.1
4′	3.45	43.4	44.5
6′	-	171.2	171.1
7′	4.53	52.1	51.6
8′	3.44; 3.37	46.6	48.5
10′	3.44; 3.26	45.1	43.8
11′	2.24; 2.11	27.2	25.9
2″	-	164.4	162.5
3″	-	**147.0**	144.3
4″	-	**161.4**	162.7
6″	-	184.4	183.5

^(a)^ the experimental chemical shifts marked in bold were assigned to corresponding nuclei based mainly on calculations. ^(b)^ calculated ^13^C chemical shifts.

**Table 2:** Summary of chemical shifts (δ > 7.5 ppm) for heavily deshielded CBP protons in DMSO [10,11] and DMSO/CF_3_COOD [21].

Chemical Shifts δ (ppm) of Selected Protons in CBP Molecule	
In DMSO [10,11]	In DMSO with CF_3_COOD [21]
8.1 ppm (singlet, 2H)	8.1 ppm (singlet, 2H)
9.5 ppm (doublet, 1H)	**8.9 ppm (singlet, 2H)**
**10.3 ppm (broad singlet, 1H)**	9.5 ppm (doublet, 1H)
12.0 ppm (broad singlet, 1H)	12.0 ppm (broad singlet, 1H)

The characteristics for signals discussed in the text are shown in bold.

**Table 3:** Summary of geometric arrangement statistics for ring pairs that may interact via π-π or stacking interaction.

	Structure	The Pair of Interacting Rings	Perpendicular Inter-Planar Distance $d_{⊥}$ (Å)	Lateral Displacement $d_{∥}$ (Å)	Tilt (°)
zwitterionic	1	thiadiazole-dihydrothiazine	3.95 ± 0.25	1.34 ± 0.77	14.7 ± 9.6
	2	dihydrothiazine-thiadiazole	3.94 ± 0.25	1.54 ± 0.77	19.6 ± 11.9
cationic	1	thiadiazole-dihydrothiazine	3.92 ± 0.24	2.00 ± 0.78	15.4 ± 10.0
	2	2-pyrrolidone-thiadiazole	3.72 ± 0.26	1.30 ± 0.59	22.2 ± 10.6
		thiadiazole-2-pyrrolidone	3.72 ± 0.26	1.28 ± 0.59	20.9 ± 9.9

**Table 4:** Antibacterial activity of the tested agents against standard and clinical Gram-positive strains.

Strains	MIC (mg/L) [MBC (mg/L)]				
	CBP-M	CBP	CBP/MA/SBE-β-CD	SBE-β-CD	MA
*S. aureus* ATCC 29213 MSSA	0.5 [>8]	0.25 [>8]	0.25 [>8]	>32 [>32]	>200 [>200]
*S. epidermidis* ATCC 12228 MSSE	0.25 [0.5]	0.25 [0.5]	0.25 [0.5]	>32 [>32]	>200 [>200]
*S. aureus* ATCC 43300 MRSA	1 [2]	1 [1]	1 [1]	>32 [>32]	>200 [>200]
*S. aureus* 664 MRSA	4 [>8]	2 [>8]	4 [>8]	>32 [>32]	>200 [>200]
*S. aureus* 1576 MRSA	2 [>8]	2 [2]	2 [>8]	>32 [>32]	>200 [>200]
*S. aureus* 1712 MRSA	2 [2]	1 [2]	1 [2]	>32 [>32]	>200 [>200]
*S. aureus* 1991 MRSA	2 [4]	2 [2]	2 [2]	>32 [>32]	>200 [>200]
*S. aureus* 2541 MRSA	4 [>8]	4 [4]	4 [>8]	>32 [>32]	>200 [>200]
*S. pneumoniae* ATCC 49619	0.0078 [0.5]	0.0039 [0.5]	0.0078 [1]	>32 [>32]	>200 [>200]
*S. pneumoniae* MUW 2/299o21	0.0156 [0.0156]	0.0156 [0.0156]	0.0156 [0.0156]	>32 [>32]	>200 [>200]
*S. pneumoniae* MUW 3/39085	0.0625 [0.125]	0.0625 [0.0625]	0.0625 [0.0625]	>32 [>32]	>200 [>200]
*S. pneumoniae* MUW 4/cw	0.0156 [0.0156]	0.0078 [0.0156]	0.0078 [0.0156]	>32 [>32]	>200 [>200]

**Table 5:** Antibacterial activity of the tested agents against standard and clinical Gram-negative strains.

Strains	MIC (mg/L) [MBC (mg/L)]				
	CBP-M	CBP	CBP/MA/SBE-β-CD	SBE-βCD	MA
*E. coli* ATCC 25922	0.125 [0.25]	0.0625 [0.125]	0.0625 [0.0625]	>32 [>32]	>200 [>200]
*E. coli* NCTC 8196	0.0625 [0.0625]	0.03125 [0.03125]	0.03125 [0.03125]	>32 [>32]	>200 [>200]
*E. coli* NCTC 10538	0.125 [0.125]	0.0625 [0.0625]	0.0625 [0.0625]	>32 [>32]	>200 [>200]
*E. coli* MUW 77 CMY-2(+)	0.5 [1]	0.25 [0.25]	0.25 [0.25]	>32 [>32]	>200 [>200]
*K. pneumoniae* ATCC 13883	0.25 [0.25]	0.125 [0.0625]	0.0625 [0.0625]	>32 [>32]	>200 [>200]
*K. pneumoniae* MUW 78 CMY-2(+)	4 [4]	1 [4]	1 [1]	>32 [>32]	>200 [>200]
*P. aeruginosa* ATCC 27853	4 [8]	4 [>8]	2 [4]	>32 [>32]	>200 [>200]
*A. baumannii* ATCC 19606	8 [>8]	4 [1]	4 [>8]	>32 [>32]	>200 [>200]

## Data Availability

Data is contained within the article and the Supplementary Material.

## Supplementary Materials

The following supporting information can be downloaded at: https://www.mdpi.com/article/10.3390/ijms262412108/s1.
