# Supplementary material for: Physicochemical Characterisation of Ceftobiprole and Investigation of the Biological Properties of Its Cyclodextrin-Based Delivery System
Source: Int J Mol Sci. 2025 Dec 16;26(24):12108. doi: 10.3390/ijms262412108 (PMC12733258; doi:10.3390/ijms262412108)
Supplement: Supplementary file 1 [file ijms-26-12108-s001.zip › ijms-4004050-supplementary.pdf]

Supplementary Material for:

Physicochemical characterisation of ceftobiprole and investigation of the biological properties of its cyclodextrin-based delivery system

Dariusz Boczar [1,*], Wojciech Bocian [2], Krystian Małek [3], Małgorzata Milczarek [3], Agnieszka Ewa Laudy [4] and Katarzyna Michalska [1,*]

1 Department of Synthetic Drugs, National Medicines Institute, Chełmska 30/34, 00-725 Warsaw, Poland; d.boczar@nil.gov.pl (D. Boczar); k.michalska@nil.gov.pl (K. Michalska)

2 Laboratory for Analysis of Bioactive Compounds, Institute of Organic Chemistry, Polish Academy of Sciences, Kasprzaka 44/52, 01-224 Warsaw, Poland; wo.bocian@gmail.com

3 Department of Biomedical Research, National Medicines Institute, Chełmska 30/34, 00-725 Warsaw, Poland; k.malek@nil.gov.pl (K. Małek); m.milczarek@nil.gov.pl (M. Milczarek)

4 Department of Pharmaceutical Microbiology and Bioanalysis, Medical University of Warsaw, Banacha 1b, 02-097, Warsaw, Poland. alaudy@wp.pl

* Correspondence: d.boczar@nil.gov.pl (D. Boczar); k.michalska@nil.gov.pl (K. Michalska)

NMR measurements

The assignment of the recorded signals to individual atoms of the ceftobiprole (CBP) molecule was carried out using the following NMR spectra: ^{1}H NMR (in the main text), Correlation Spectroscopy (COSY, Figure S3), Rotating-frame Overhauser Enhancement Spectroscopy (ROESY, Figure S4), Heteronuclear Single-Quantum Correlation (HSQC, Figure S5) and Heteronuclear Multiple-Bond Correlation (HMBC, Figure S6), supported by the theoretical calculations. Additionally, the 1D ^{13}C NMR spectrum is shown in Figure S1.

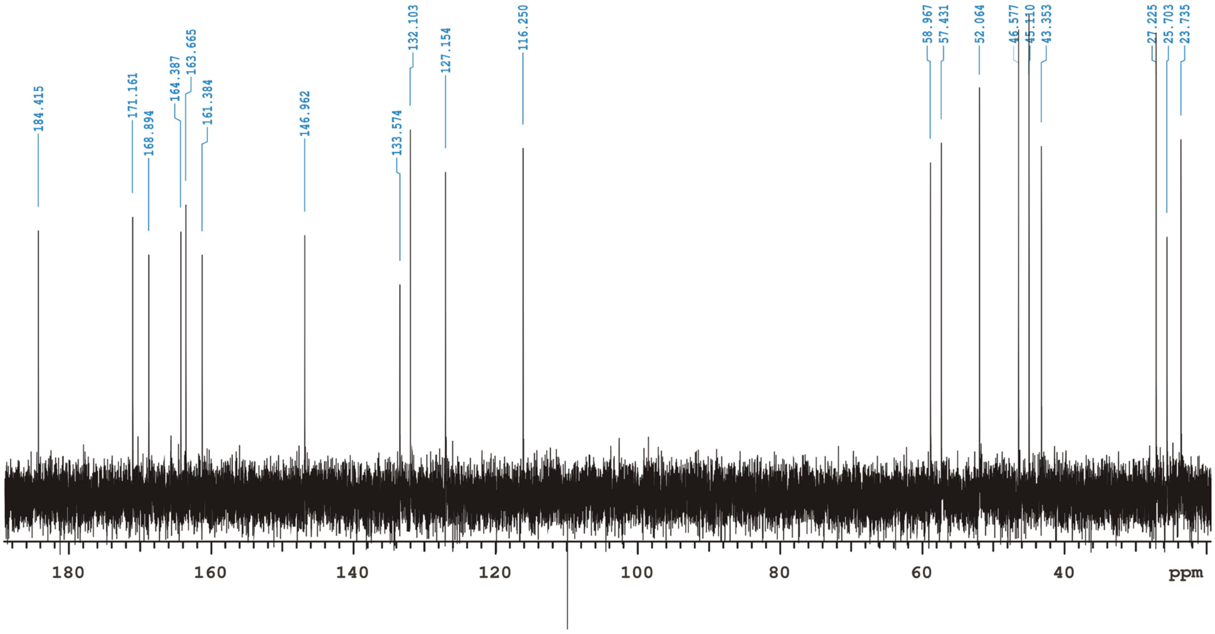

Figure S1. The ^{13}C spectrum of 3.75 mM D_2O solution of CBP freeze-dried in 0.1 M HCOOH.

Correct identification of the protons within the cephem group requires particular attention. The singlet at 6.91 ppm is unambiguously assigned to H1’, as it belongs to the system of four conjugated double bonds. COSY and ROESY data indicate a strong correlation between this signal and the broad multiplet at 3.60 - 3.72 ppm (Figure S2A), attributable to the two protons at C2. ROESY data further show that only the higher-frequency portion of this multiplet ($\delta > 3.66$ ppm), exhibits a cross-peak with the signal at 5.14 ppm. Since the ROESY cross-peak intensity reflects spatial proximity of the interacting atoms, two important conclusions can be drawn (see Figure S2): (i) the signal at 5.14 ppm corresponds to H6, and (ii) the multiplet at 3.60 - 3.72 ppm consists of two parts – the signal at 3.66 - 3.72 ppm arises from the proton located on the same side of the cephem ring as H6 proton, whereas 3.60 - 3.66 ppm originates from the proton located on the opposite side. The signal assigned to H6 at 5.14 ppm forms a cross-peak with a signal at 5.77 ppm, indicating the latter as H7. HMBC data additionally support this assignment by showing clear cross-peaks between C6 and H2, as well as between H6 and C2. NMR spectra acquired in DMSO further corroborate these conclusions, although the chemical shifts shift slightly (to 5.07 and 5.75 ppm instead of 5.14 and 5.77 ppm, respectively), the 5.75-ppm signal appears as a doublet of doublets due to coupling with H1”, observable only in DMSO. A contradictory pattern emerges in the COSY spectrum, where 3.70 ppm couples with 5.77 ppm and 5.77 ppm couples with 5.14 ppm, but no cross-peak is observed between 3.69 ppm and 5.14 ppm. An interpretation based solely on COSY would suggest the opposite assignment: 5.77 ppm as H6 and 5.14 ppm as H7. Nevertheless, the combined evidence—ROESY, HMBC, spectra acquired in DMSO, and DFT-calculated chemical shifts for C6 and C7—consistently supports the assignment of 5.14 ppm to H6 and 5.77 ppm to H7.

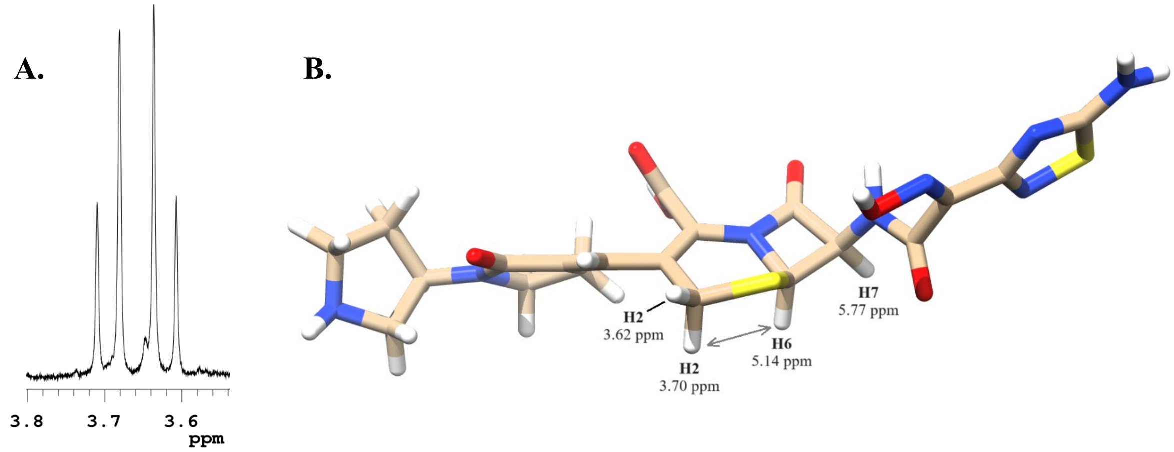

Figure S2. (A) ^{1}H NMR spectrum of 3.75 mM D_2O solution of CBP freeze-dried in 0.1 M HCOOH, in the range 3.6 – 3.8 ppm; (B) 3D structure of CBP showing the H2 protons on the same and opposite side of the cephem ring with respect to the H6 and H7 protons.

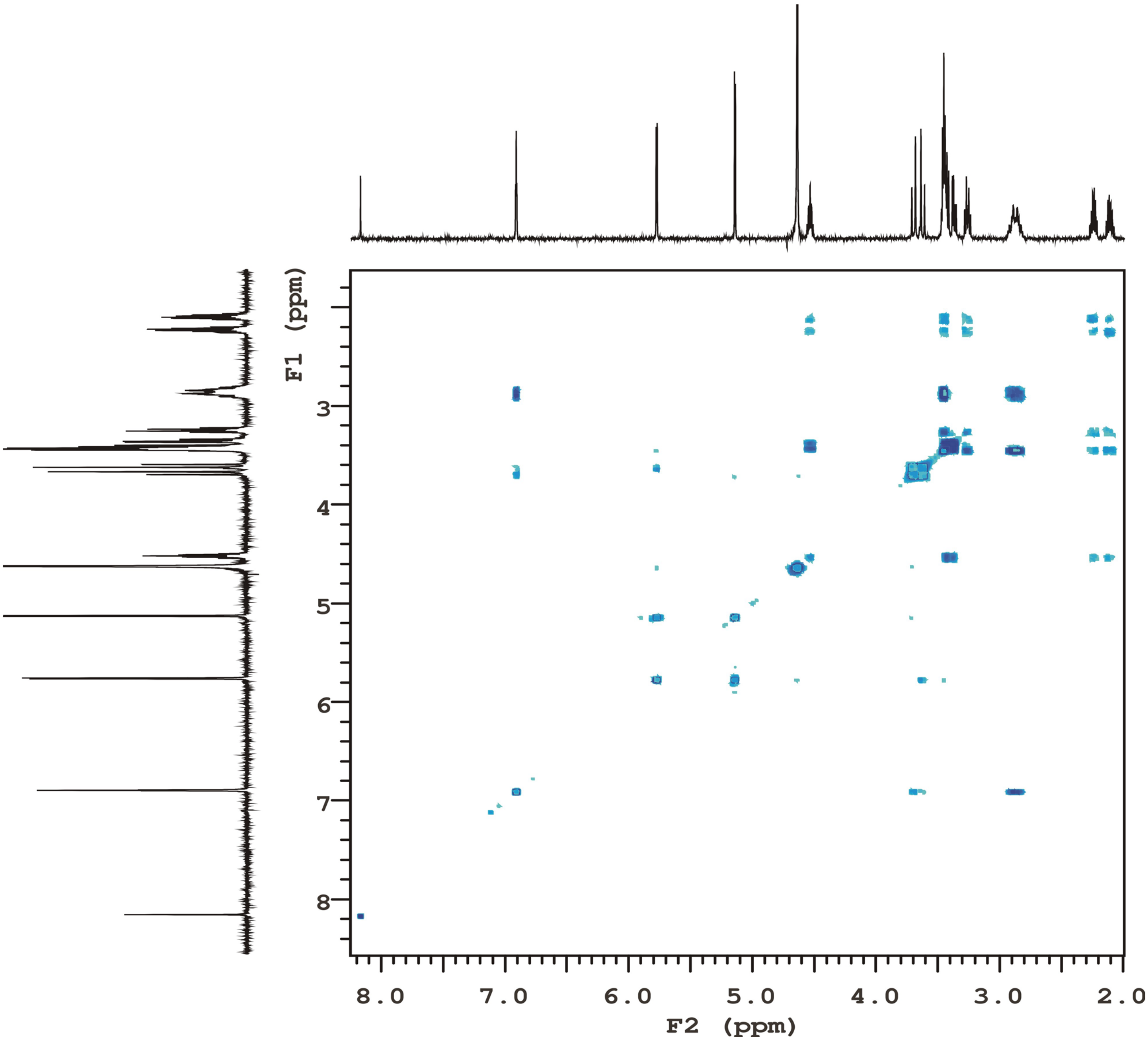

Figure S3. COSY spectrum of a 3.75 mM solution in D_2O of CBP freeze-dried in 0.1 M HCOOH.

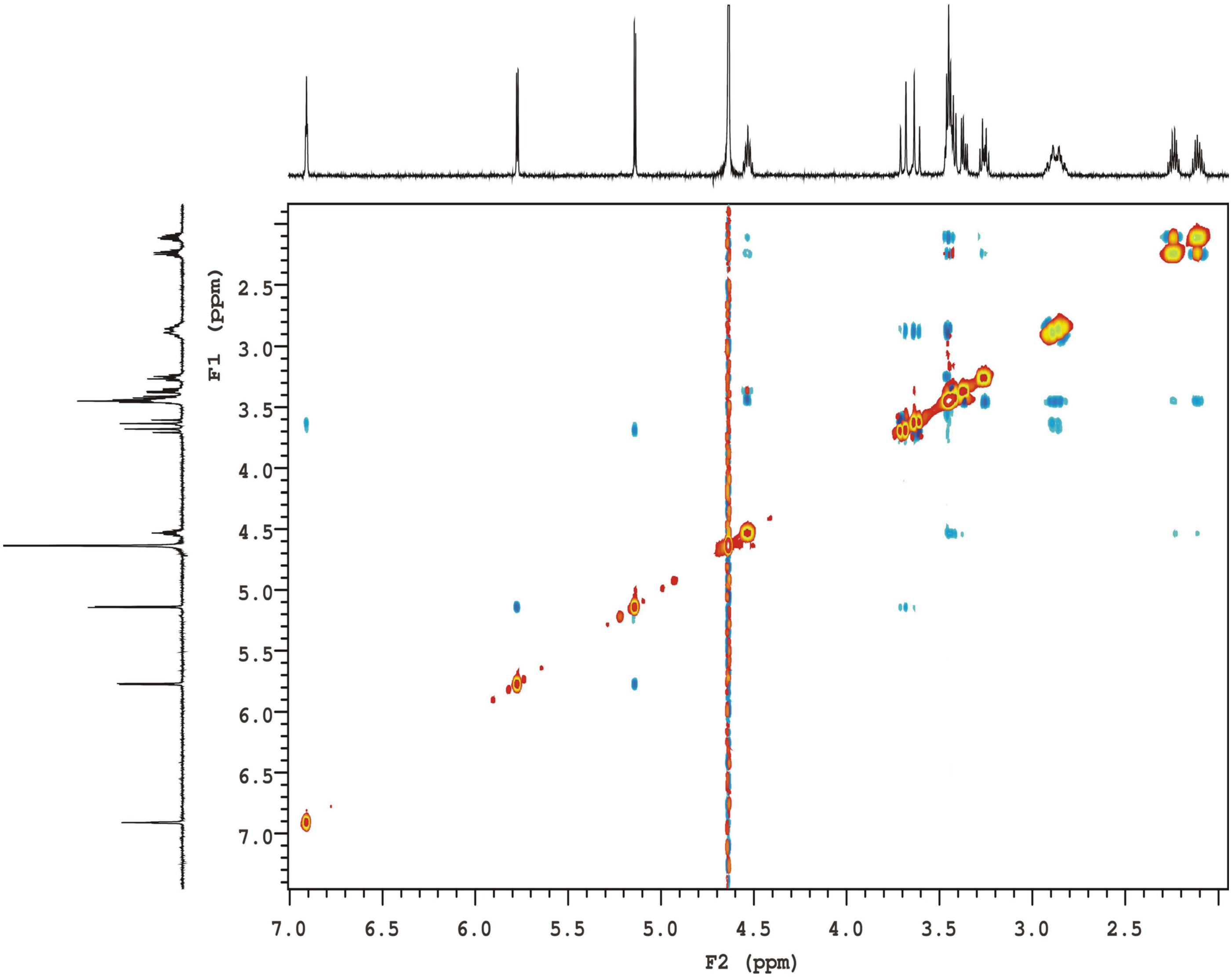

Figure S4. ROESY spectrum of a 3.75 mM solution in D_2O of CBP freeze-dried in 0.1 M HCOOH.

(a)

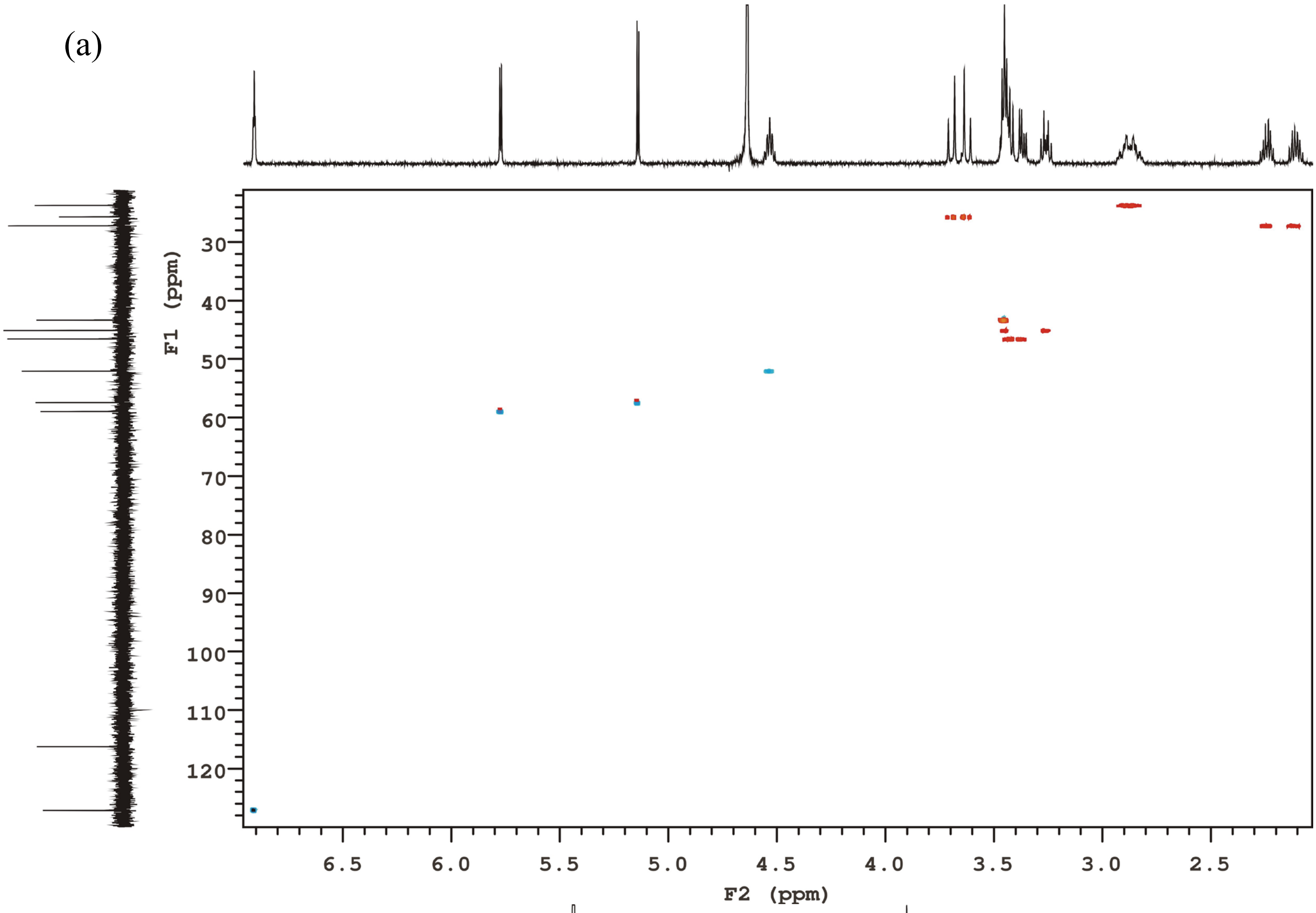

(b)

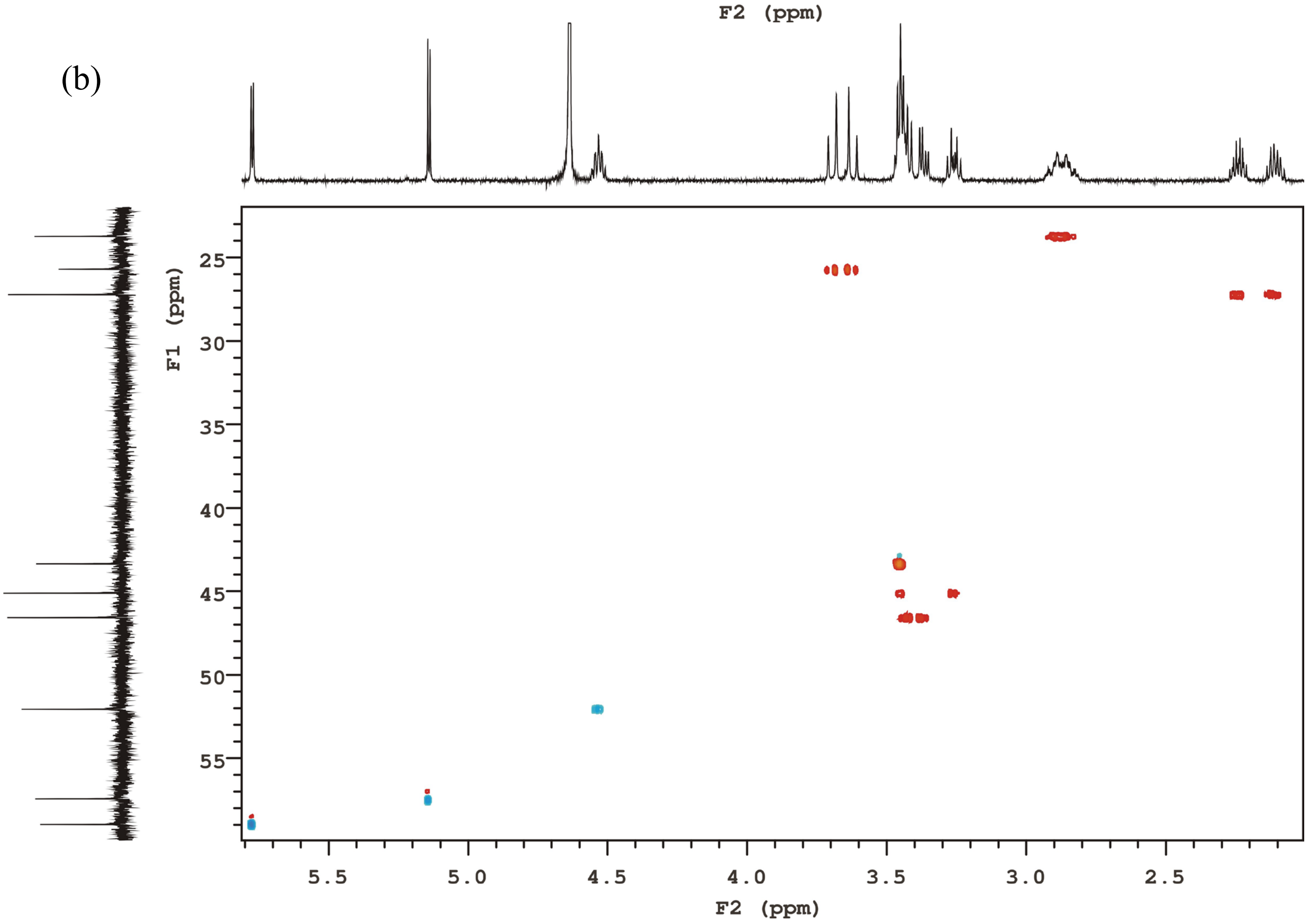

Figure S5. HSQC spectrum of a 3.75 mM solution in D_2O of CBP freeze-dried in 0.1 M HCOOH: (a) full range of chemical shifts, (b) magnification to 2.0 – 5.8 ppm for ^{1}H and 22 – 60 ppm for ^{13}C NMR.

(a)

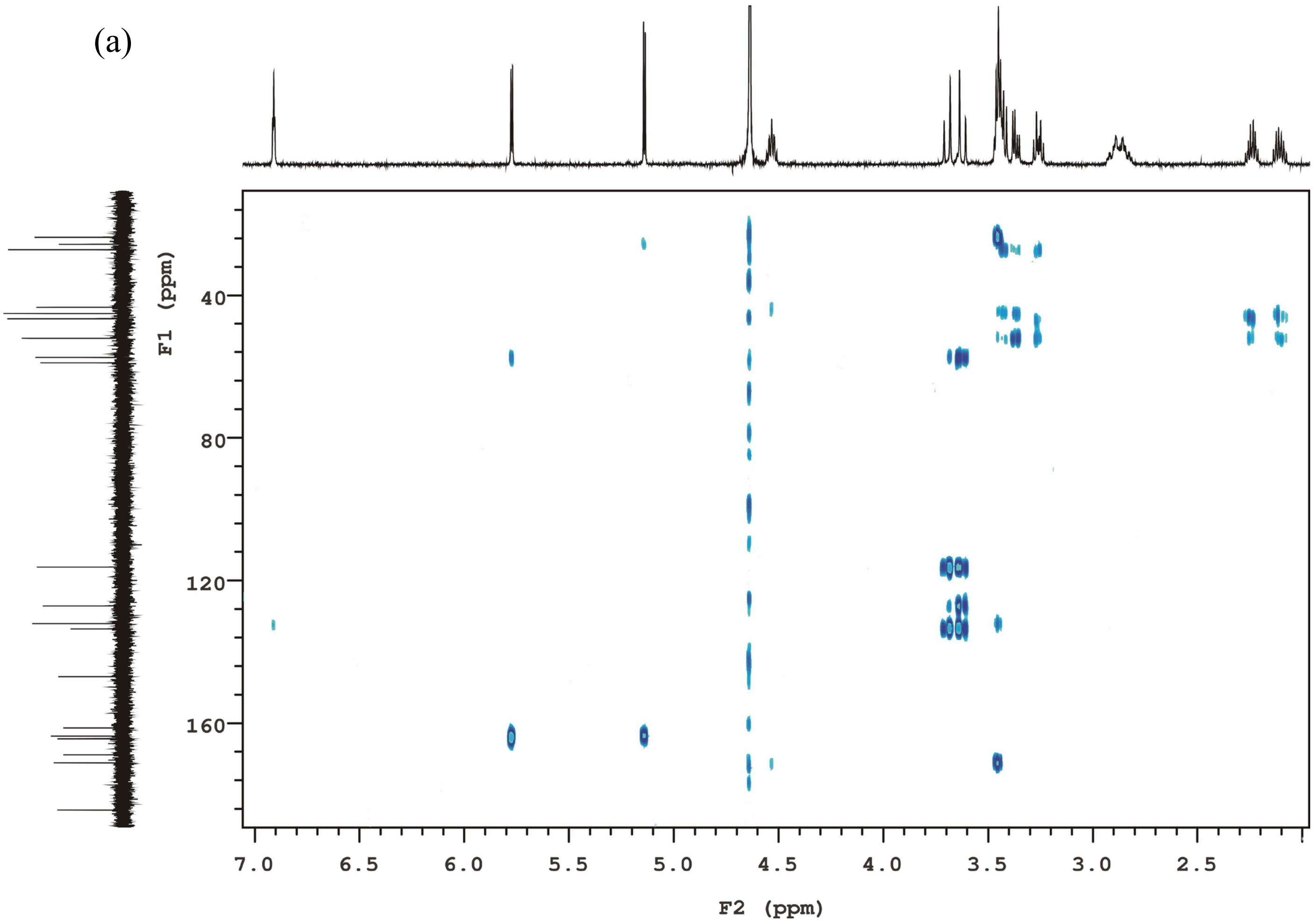
F1 (ppm)
40
80
120
160
7.0
6.5
6.0
5.5
5.0
4.5
4.0
3.5
3.0
2.5
F2 (ppm)

(b)

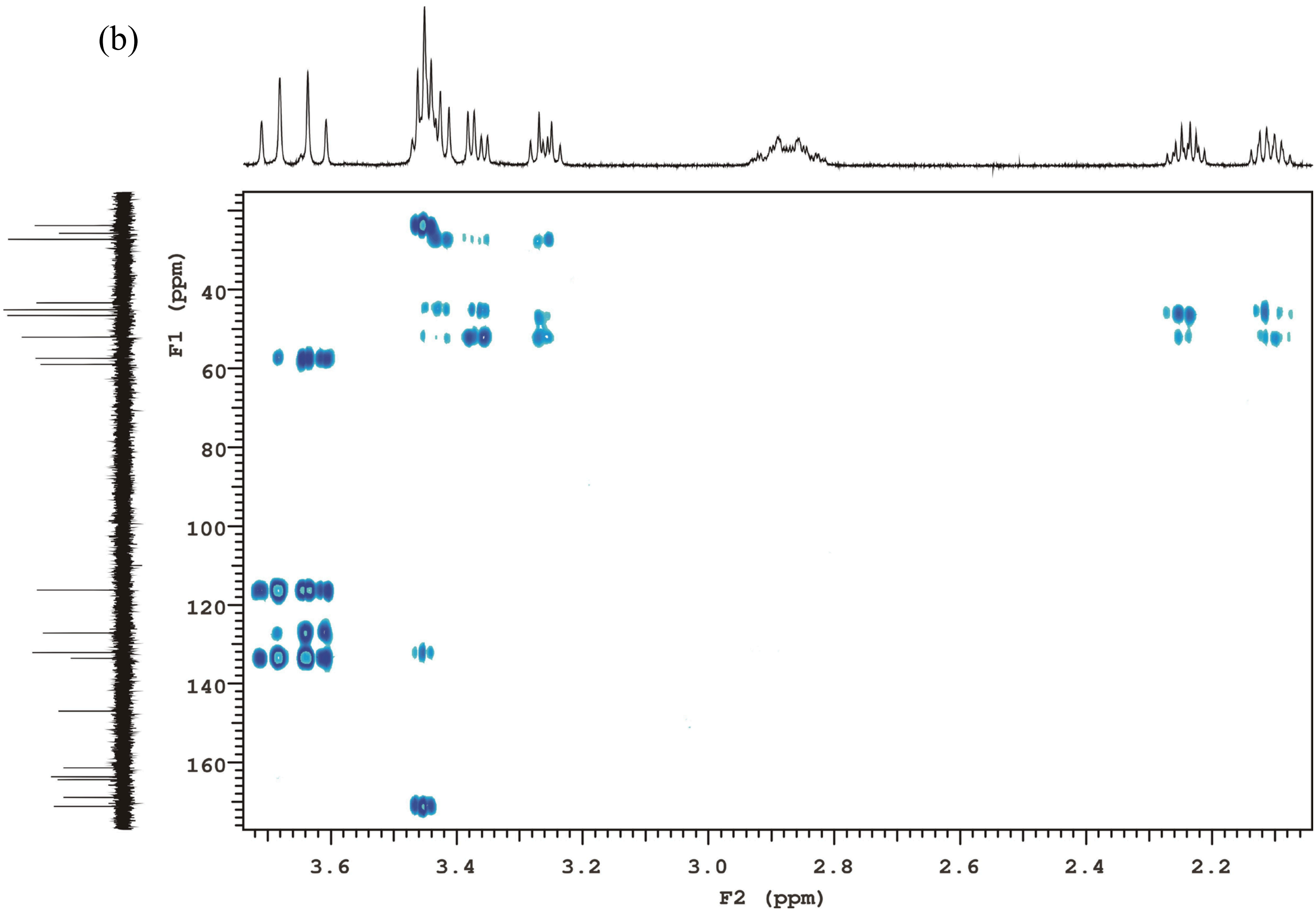
F1 (ppm)
40
60
80
100
120
140
160
3.6
3.4
3.2
3.0
2.8
2.6
2.4
2.2
F2 (ppm)

(c)

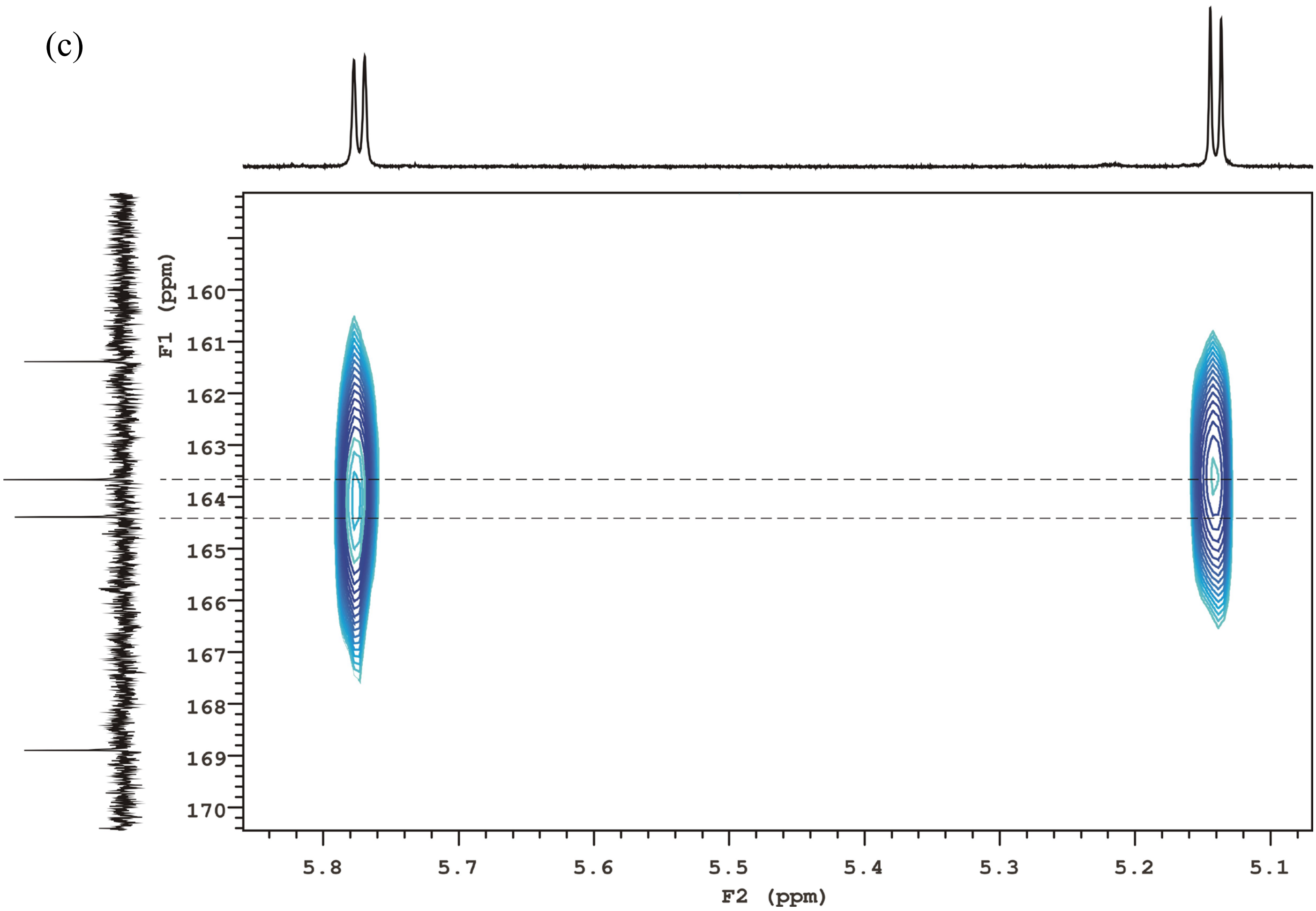

Figure S6. HMBC spectrum of a 3.75 mM solution in D_2O of CBP freeze-dried in 0.1 M HCOOH: (a) full range of chemical shifts, (b) magnification to 2.0 – 3.7 ppm for ^{1}H NMR, (c) magnification to 5.1 – 5.8 ppm for ^{1}H NMR and 160 - 170 ppm for ^{13}C NMR.

DFT Calculations

Table S1 presents energy differences for the 40 proposed least-energy CBP conformers. Additionally, the results of fitting the calculated shieldings to experimental chemical shifts, depending on the number of included CBP conformers, with and without applying the Boltzmann population weights, are compared. As shown in Table S1, the obtained R^2 values increase markedly with the number of averaged structures, reaching a maximum at fifteen. When Boltzmann population weights are applied, this improvement plateaus after averaging only four structures, which reflects the fact that Boltzmann weights become negligible for conformers beyond the fifth. Figure S7 compares the results of fitting the calculated to experimental ^{13}C chemical shifts, with and without Boltzmann weighting, and reveals the largest deviations from linearity for atoms 3, 4, 1′, and 2′. These deviations are substantially reduced when fittings are performed without Boltzmann weights. Atoms 3, 4, 1′, and 2′ belong to the conjugated system spanning the dihydrothiazine and 2-pyrrolidone rings and their linker. The three lowest-energy conformers (structures 1–3) preserve the spatial arrangement of this fragment, whereas subsequent conformers introduce conformational changes in this region. These observations clearly suggest that the accurate representation of CBP conformational space and reliable simulating the NMR spectrum require consideration of substantially more conformations than those emphasized by the Boltzmann distribution. This discrepancy can likely be attributed to limitations of the DFT protocol used, in particular neglecting the intermolecular hydrogen-bonding interactions with solvent and between CBP molecules. However, explicit inclusion of these effects in the calculations would be technically very challenging and computationally prohibitive.

Table S1. Energies of the CBP conformers and coefficients of determination from fits between calculated and experimental NMR data, with or without Boltzmann population weighting. Fifteen low energy structures used to final NMR chemical shifts calculation are shown in bold.

**Structure number**	**Energy difference (to the lowest energy structure), kcal/mol [(a)]**		**Coefficients of determination R^2 [(c)]**	**Boltzmann population weights [(d)]**	**Boltzmann weighted R^2 [(e)]**
	**ΔE (electronic)**	**ΔE (ZPVE) [(b)]**			
**1**	**0**	**0**	**0.99705**	**0.77936**	**0.99705**
**2**	**0.74**	**0.79**	**0.99711**	**0.20567**	**0.99708**
**3**	**2.76**	**2.81**	**0.99736**	**0.00682**	**0.99709**
**4**	**3.07**	**2.84**	**0.99790**	**0.00648**	**0.99711**

**5**	**3.74**	**3.97**	**0.99817**	**0.00096**	**0.99711**
**6**	**4.49**	**4.46**	**0.99822**	**0.00042**	**0.99711**
**7**	**5.62**	**5.49**	**0.99823**	**0.00007**	**0.99711**
**8**	**5.92**	**5.64**	**0.99842**	**0.00006**	**0.99711**
**9**	**5.86**	**5.65**	**0.99849**	**0.00006**	**0.99711**
**10**	**6.11**	**5.75**	**0.99864**	**0.00005**	**0.99711**
**11**	**6.42**	**6.41**	**0.99873**	**0.00002**	**0.99711**
**12**	**6.65**	**6.54**	**0.99877**	**0.00001**	**0.99711**
**13**	**6.91**	**6.68**	**0.99882**	**0.00001**	**0.99711**
**14**	**7.55**	**7.31**	**0.99886**	**0.00000**	**0.99711**
**15**	**7.59**	**7.33**	**0.99892**	**0.00000**	**0.99711**
16	7.71	7.74	0.99891	0.00000	0.99711
17	8.12	8.19	0.99891	0.00000	0.99711
18	8.18	8.21	0.99890	0.00000	0.99711
19	8.78	8.36	0.99890	0.00000	0.99711
20	8.34	8.41	0.99890	0.00000	0.99711
21	8.25	8.51	0.99887	0.00000	0.99711
22	8.93	8.60	0.99891	0.00000	0.99711
23	9.23	9.14	0.99892	0.00000	0.99711
24	9.25	9.14	0.99890	0.00000	0.99711
25	9.26	9.14	0.99887	0.00000	0.99711
26	9.28	9.39	0.99883	0.00000	0.99711
27	9.51	9.41	0.99884	0.00000	0.99711
28	9.99	9.61	0.99888	0.00000	0.99711
29	9.34	9.69	0.99883	0.00000	0.99711
30	9.72	9.91	0.99878	0.00000	0.99711
31	9.94	9.96	0.99879	0.00000	0.99711
32	10.39	9.96	0.99880	0.00000	0.99711
33	10.04	10.07	0.99885	0.00000	0.99711
34	10.94	10.87	0.99883	0.00000	0.99711
35	11.65	11.55	0.99880	0.00000	0.99711
36	12.02	11.96	0.99884	0.00000	0.99711
37	12.62	12.20	0.99887	0.00000	0.99711
38	12.37	12.47	0.99885	0.00000	0.99711
39	12.94	12.94	0.99882	0.00000	0.99711
40	12.94	12.94	0.99878	0.00000	0.99711

(a) Energies were calculated at the DFT B3LYP/6-311+G(d,p) level.

(b) E (ZPVE) denotes the electronic energy with harmonic zero-point vibrational energy correction.

(c) Coefficients of determination from fittings of averaged calculated shieldings to the experimental NMR data calculated over an increasing number of structures.

(d) Normalized equilibrium population weights for the given structures at room temperature calculated using the Boltzmann distribution law: $w_i \propto exp\left(-\frac{\Delta E_i^{ZPVE}}{k_B T}\right)$

(e) Coefficients of determination from fittings of averaged calculated shieldings to the experimental NMR data calculated over an increasing number of structures. The shieldings were averaged using Boltzmann population weights.

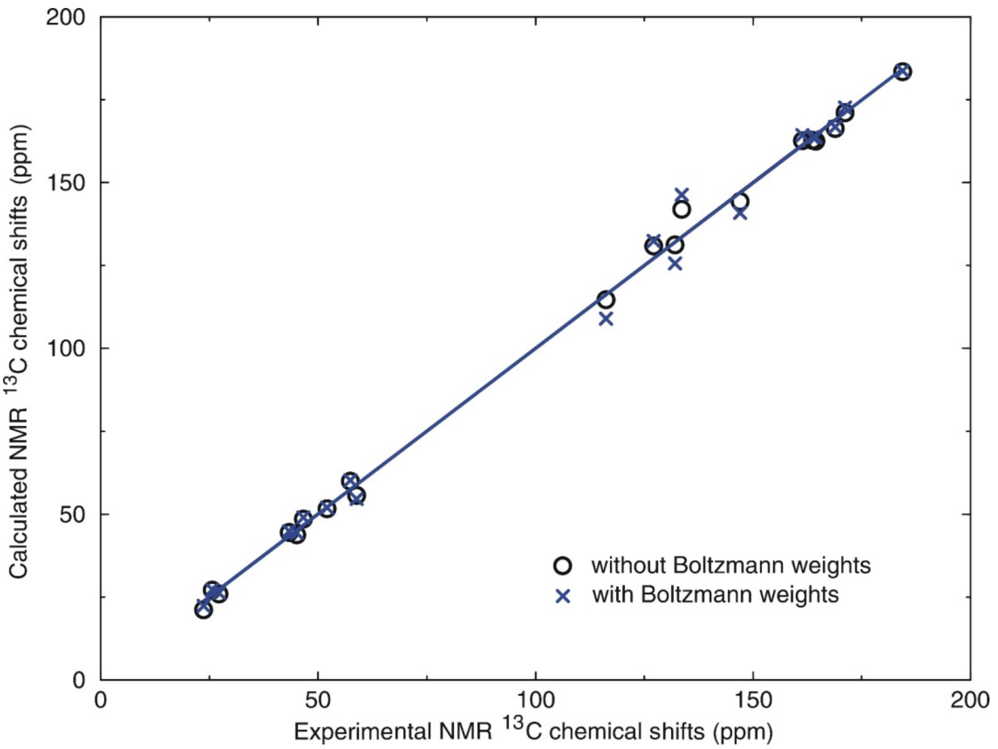

Figure S7. Comparison of fittings of averaged calculated shieldings to experimental NMR data. Fifteen structures were averaged both with and without Boltzmann weights.

The simulation of the IR spectra was performed using DFT calculations for the fifteen structures mentioned above, which are thermodynamically most preferred. The frequencies were calculated at the same level of theory (DFT/BLYP/6-311+G(d,p)) and scaled by a factor of 0.984 to reduce the calculation error that was caused by ignoring the anharmonic effect. The following procedure was applied to improve the agreement between the theory and experiment, using a Python script: for each individual frequency ν_i of a normal mode of a specified conformer j, a contribution y_{ij} to the overall IR spectrum was calculated assuming the Lorentzian distribution:

$$y_{ij}(\bar{\nu}) = \frac{I_{ij}}{\bar{\nu}_{ij}^2} \frac{\gamma^2}{\left(\bar{\nu} - f\,\bar{\nu}_{ij}\right)^2 + \gamma^2} \quad (1)$$

Here, the index i denotes individual normal modes and j refers to individual conformations. This equation expresses y_{ij} (a contribution to the spectrum) as a function of wavenumber $\bar{\nu}$, using: $\bar{\nu}_{ij}$ – the corresponding wavenumber obtained from DFT calculations, I_{ij} – the intensity of this vibration, obtained from the same DFT calculations, γ – the half width at half maximum (HWHM) of the peak, and f – the scaling factor for the wavenumber. The intensity was additionally scaled (by means of dividing I_{ij} by $\bar{\nu}_{ij}^2$) to further improve the agreement

between theoretical and experimental results. Then, the contributions for the same normal mode were summed up for all the fifteen conformations:

$$y_i(\bar{\nu}) = \sum_{j=1}^{15} y_{ij}(\bar{\nu}) \qquad (2)$$

The individual contributions from each normal mode $y_i(\bar{\nu})$ were presented in Figure S8 in the form of dashed lines, labelled as line D.

Finally, the summation of the contributions from all the determined normal modes allowed to simulate the IR spectrum:

$$y(\bar{\nu}) = \sum_i y_i(\bar{\nu}) \qquad (3)$$

This result is shown in Figure S8, line C.

The obtained theoretical spectrum was compared with experimental spectra, recorded in an ATR mode for a crystalline unprocessed CBP obtained from the manufacturer (Figure S8B) as well as an its amorphous counterpart, freeze-dried in water (Figure S8A). The optimum results were obtained for FWHM of 9 cm^{-1} and scaling factor of 0.984.

Since the CBP molecule contains N = 58 atoms and is non-linear, it has 3N – 6 = 168 possible normal modes. However, as can be seen in Figure S8, the number of peaks and bands observed experimentally in IR spectrum is significantly lower because each signal is characterised by a specified peak width, causing the contributions from many normal modes of similar frequencies to coalesce into one band. It is also worth mentioning that some modes involve vibrations of multiple groups in different parts of the molecule at similar frequencies. As shown in Figure S8, the agreement between theoretical and experimental spectra is not perfect, even after adjusting the wavenumber scaling factor and peak width as well as scaling the intensity. This discrepancy may be attributed to the presence of intermolecular hydrogen bonds in the solid sample, both in its amorphous and crystalline forms. Given these issues, interpretation of the IR spectrum in terms of individual molecular vibrations was not performed.

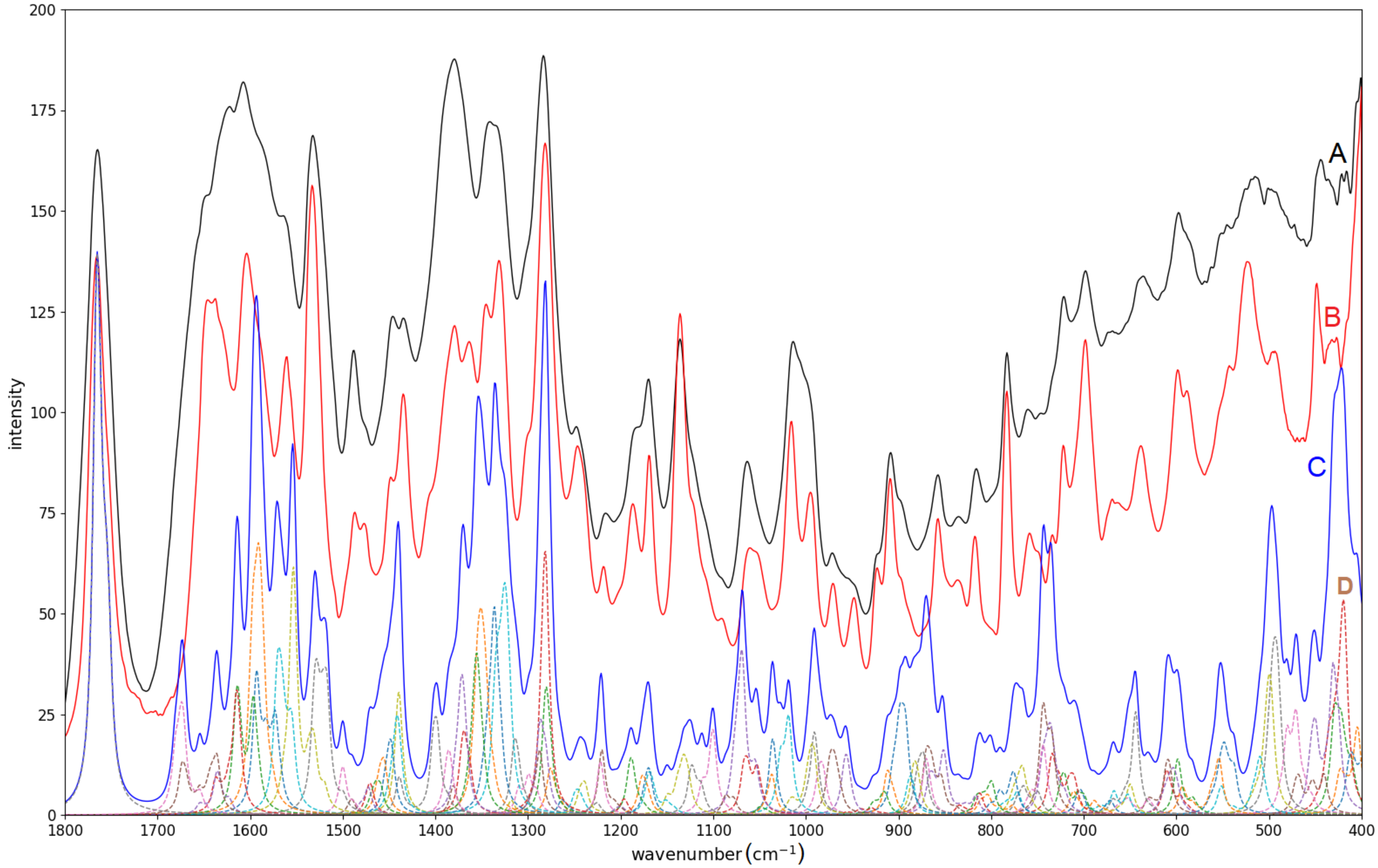

Figure S8. The experimental ATR-IR spectra of **(A)** CBP freeze-dried in water and **(B)** unprocessed CBP; accompanied with **(C)** calculated IR spectrum based on the sum of (**D**) contributions of individual modes, presented with dashed lines.

Calculated atomic coordinates and magnetic shieldings for the analysed conformers:

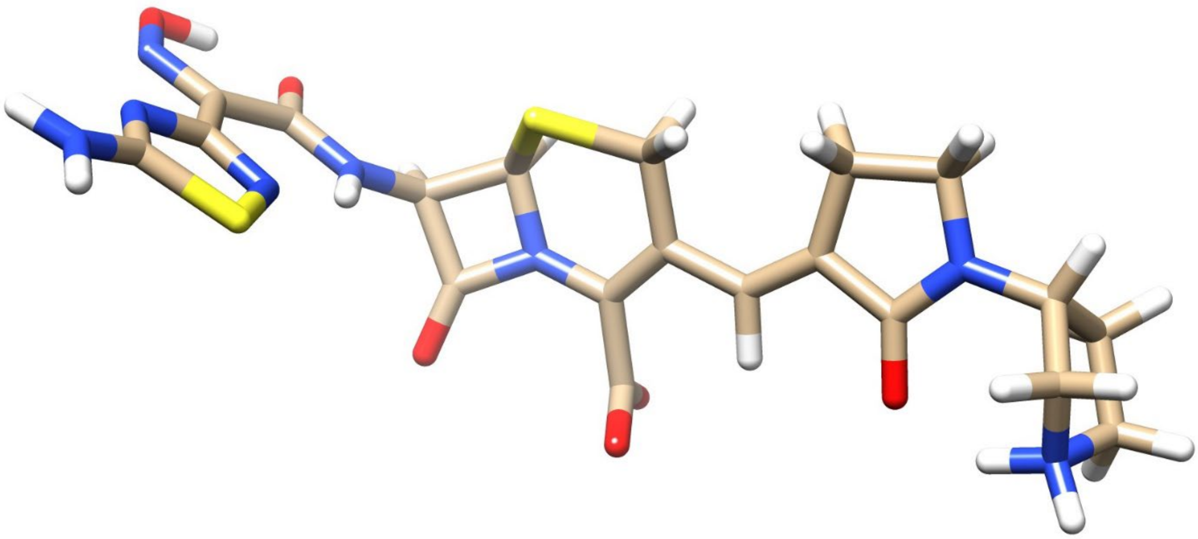

1

Energy = −2461.61846061 Hartree

Atomic coordinates (Å):

	X	Y	Z
1 S	−0.982954	−0.045738	−1.587091
2 C	0.855906	−0.214601	−1.568751
3 C	1.463138	−0.705924	−0.263910
4 C	0.775242	−1.476551	0.631406
5 N	−0.517870	−1.893348	0.334280
6 C	−1.229303	−1.729769	−0.938676
7 C	−2.553244	−2.074148	−0.181725
8 C	−1.659437	−2.126948	1.086129
9 O	−1.853183	−2.275438	2.266081
10 C	1.336412	−1.997494	1.971885
11 O	1.520598	−1.152657	2.874626
12 O	1.545969	−3.231887	1.995736
2a H	1.211042	0.790829	−1.787208
2b H	1.137870	−0.853852	−2.408727
6 H	−0.925709	−2.462987	−1.685720
7 H	−2.951354	−3.045201	−0.471376
1” N	−3.620625	−1.115246	−0.150315
2” C	−4.830788	−1.370091	−0.679622
3” C	−5.901699	−0.313421	−0.570243
4” C	−5.684081	1.006977	0.080187
5” N	−6.681665	1.938999	0.097865
6” C	−6.262792	3.018157	0.720753

7” S	−4.597379	2.843459	1.283119
8” N	−4.527256	1.285271	0.643608
1” H	−3.502758	−0.204648	0.304584
9” O	−5.094553	−2.444416	−1.253682
10” N	−7.098509	−0.484687	−1.045464
11” O	−7.420274	−1.640844	−1.661789
11” H	−6.606235	−2.226322	−1.651700
12” N	−6.994560	4.128981	0.890005
12”a H	−7.964147	4.119370	0.610840
12”b H	−6.674463	4.896378	1.458244
1’ C	2.835314	−0.358045	0.048377
2’ C	3.785030	0.262367	−0.687060
3’ C	3.813652	0.814226	−2.096308
4’ C	5.312007	1.055250	−2.376448
5’ N	5.934710	1.017317	−1.045871
6’ C	5.122034	0.484665	−0.094761
7’ C	7.369022	1.252635	−0.883220
8’ C	7.713696	2.097903	0.355692
9’ N	8.011244	1.067575	1.405384
10’ C	8.819797	0.006761	0.705521
11’ C	8.202990	−0.059572	−0.702974
1’ H	3.164664	−0.646286	1.041986
3’a H	3.270588	1.762271	−2.155967
3’b H	3.382001	0.139081	−2.834181
4’a H	5.501914	2.018333	−2.852505
4’b H	5.735831	0.268368	−3.008759
12’ O	5.469474	0.237439	1.075997
7’ H	7.686475	1.790764	−1.773748
8’a H	6.913420	2.752266	0.691427
8’b H	8.620863	2.675855	0.186111
9’a H	8.477213	1.449464	2.228406
9’b H	7.084154	0.664358	1.660970
10’a H	9.855008	0.343047	0.690042
10’b H	8.749506	−0.920870	1.268639
11’a H	8.984477	−0.131179	−1.457716
11’b H	7.555056	−0.928466	−0.809798

SCF GIAO magnetic shielding (ppm):

1 S	Isotropic = 485.9481 Anisotropy = 224.8117
2 C	Isotropic = 148.6978 Anisotropy = 20.4494
3 C	Isotropic = 67.5082 Anisotropy = 137.0833
4 C	Isotropic = 30.2937 Anisotropy = 151.3892
5 N	Isotropic = 64.4551 Anisotropy = 116.5154
6 C	Isotropic = 115.9163 Anisotropy = 31.4566
7 C	Isotropic = 121.5676 Anisotropy = 47.7431
8 C	Isotropic = 13.0929 Anisotropy = 77.5423
9 O	Isotropic = −60.5734 Anisotropy = 520.2293
10 C	Isotropic = 9.8751 Anisotropy = 85.4300

11 O	Isotropic = −28.5045 Anisotropy = 369.1705
12 O	Isotropic = −22.7110 Anisotropy = 366.3047
2a H	Isotropic = 28.3711 Anisotropy = 13.0596
2b H	Isotropic = 27.9595 Anisotropy = 9.5843
6 H	Isotropic = 26.7334 Anisotropy = 7.9788
7 H	Isotropic = 26.0225 Anisotropy = 5.1843
1" N	Isotropic = 118.8507 Anisotropy = 75.5378
2" C	Isotropic = 12.6471 Anisotropy = 106.6695
3" C	Isotropic = 35.9294 Anisotropy = 135.3516
4" C	Isotropic = 11.6781 Anisotropy = 83.1522
5" N	Isotropic = −3.2248 Anisotropy = 256.5279
6" C	Isotropic = −6.4018 Anisotropy = 126.3285
7" S	Isotropic = 79.8676 Anisotropy = 276.3228
8" N	Isotropic = −9.5540 Anisotropy = 229.5750
1" H	Isotropic = 21.0876 Anisotropy = 17.5532
9" O	Isotropic = 9.4522 Anisotropy = 462.5605
10" N	Isotropic = −230.5483 Anisotropy = 477.0850
11" O	Isotropic = 52.1249 Anisotropy = 334.7872
11" H	Isotropic = 15.3469 Anisotropy = 20.2963
12" N	Isotropic = 167.6861 Anisotropy = 87.7624
12"a H	Isotropic = 26.1782 Anisotropy = 12.5287
12"b H	Isotropic = 26.5810 Anisotropy = 9.8883
1' C	Isotropic = 44.2411 Anisotropy = 160.2504
2' C	Isotropic = 50.9686 Anisotropy = 113.2420
3' C	Isotropic = 153.4428 Anisotropy = 20.1194
4' C	Isotropic = 131.0568 Anisotropy = 53.0089
5' N	Isotropic = 94.5081 Anisotropy = 100.8489
6' C	Isotropic = 3.9368 Anisotropy = 103.4176
7' C	Isotropic = 124.1483 Anisotropy = 28.0176
8' C	Isotropic = 126.9247 Anisotropy = 38.7665
9' N	Isotropic = 182.5359 Anisotropy = 38.8866
10' C	Isotropic = 131.8167 Anisotropy = 55.4173
11' C	Isotropic = 149.7029 Anisotropy = 39.4559
1' H	Isotropic = 24.2702 Anisotropy = 11.7327
3'a H	Isotropic = 28.8231 Anisotropy = 7.1887
3'b H	Isotropic = 28.9190 Anisotropy = 7.6031
4'a H	Isotropic = 28.4264 Anisotropy = 6.7023
4'b H	Isotropic = 28.2582 Anisotropy = 6.6985
12' O	Isotropic = 33.7406 Anisotropy = 454.7172
7' H	Isotropic = 27.9546 Anisotropy = 6.2616
8'a H	Isotropic = 28.2482 Anisotropy = 7.0347
8'b H	Isotropic = 28.4438 Anisotropy = 9.9197
9'a H	Isotropic = 27.1229 Anisotropy = 16.9577
9'b H	Isotropic = 21.5848 Anisotropy = 21.4763
10'a H	Isotropic = 28.6937 Anisotropy = 11.0382
10'b H	Isotropic = 28.0462 Anisotropy = 9.2464
11'a H	Isotropic = 29.2080 Anisotropy = 9.7756
11'b H	Isotropic = 29.8235 Anisotropy = 6.2173

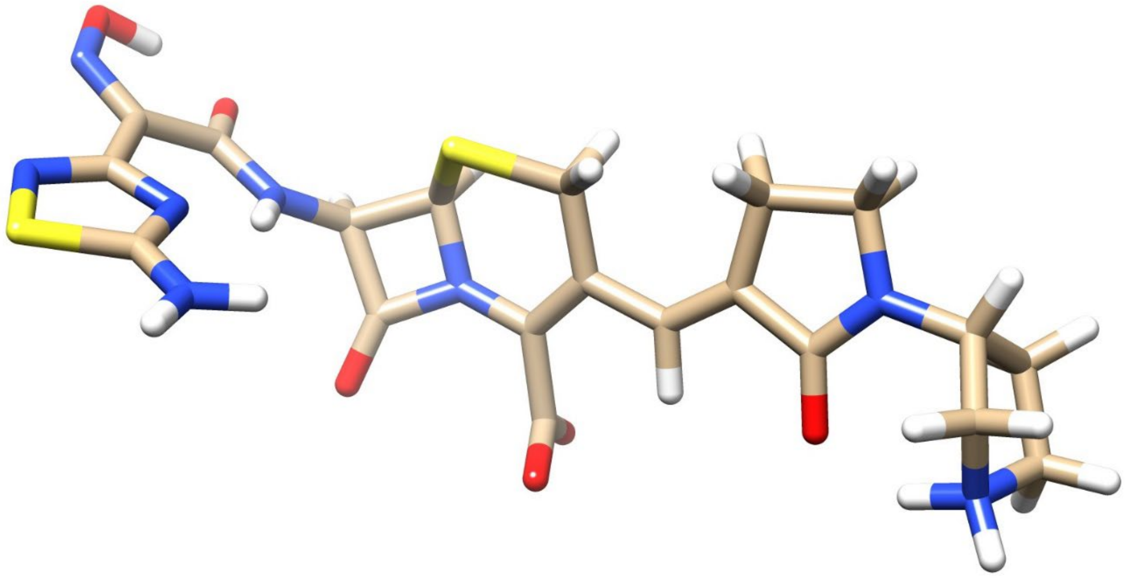

2

Energy = −2461.61727439 Hartree

Atomic coordinates (Å):

	X	Y	Z
1 S	−1.007377	−0.309889	−1.586972
2 C	0.828047	−0.486999	−1.498586
3 C	1.411330	−0.678497	−0.107166
4 C	0.696131	−1.202775	0.933232
5 N	−0.599926	−1.659465	0.719019
6 C	−1.285258	−1.795787	−0.570479
7 C	−2.626406	−1.944785	0.219488
8 C	−1.758675	−1.687641	1.480387
9 O	−1.976059	−1.537450	2.655949
10 C	1.225259	−1.393604	2.370634
11 O	1.426159	−0.355984	3.038549
12 O	1.394017	−2.588585	2.704761
2a H	1.198318	0.441943	−1.929078
2b H	1.115999	−1.299368	−2.169979
6 H	−0.975595	−2.684480	−1.119896
7 H	−3.015796	−2.960425	0.168794
1” N	−3.698404	−1.019057	−0.008955
2” C	−4.874758	−1.404636	−0.538641
3” C	−5.946241	−0.361900	−0.723379
4” C	−5.847099	1.009692	−0.149056
5” N	−4.612352	1.556899	0.086227
6” C	−4.748018	2.774954	0.573947
7” S	−6.448001	3.200736	0.723473
8” N	−6.930384	1.694511	0.128102

1” H	−3.586535	−0.019256	0.181712
9” O	−5.101781	−2.579174	−0.883803
10” N	−7.037186	−0.590278	−1.386217
11” O	−7.266749	−1.819155	−1.901488
11” H	−6.509480	−2.410512	−1.623507
12” N	−3.728464	3.571840	0.914615
12”a H	−2.782350	3.263012	0.749422
12”b H	−3.872783	4.513883	1.238776
1’ C	2.792860	−0.309488	0.131411
2’ C	3.762875	0.096480	−0.718340
3’ C	3.804978	0.320123	−2.214777
4’ C	5.308183	0.462492	−2.535104
5’ N	5.935079	0.684185	−1.224478
6’ C	5.109339	0.395045	−0.183719
7’ C	7.377586	0.898488	−1.114328
8’ C	7.760725	1.963516	−0.072640
9’ N	8.034305	1.156230	1.162057
10’ C	8.803248	−0.049902	0.688351
11’ C	8.169129	−0.378881	−0.674967
1’ H	3.112029	−0.378186	1.166855
3’a H	3.280636	1.240364	−2.488551
3’b H	3.360846	−0.494658	−2.785525
4’a H	5.519823	1.302432	−3.198464
4’b H	5.711524	−0.446972	−2.991794
12’ O	5.453622	0.391455	1.013778
7’ H	7.705147	1.234623	−2.095769
8’a H	6.984857	2.698129	0.126702
8’b H	8.684058	2.466180	−0.356647
9’a H	8.518535	1.681174	1.889952
9’b H	7.096400	0.846350	1.495465
10’a H	9.847861	0.244206	0.600613
10’b H	8.710364	−0.842283	1.427494
11’a H	8.939354	−0.629876	−1.402464
11’b H	7.491106	−1.228110	−0.600278

SCF GIAO magnetic shielding (ppm):

1 S	Isotropic = 484.2652 Anisotropy = 230.8341
2 C	Isotropic = 148.7933 Anisotropy = 20.5541
3 C	Isotropic = 67.2517 Anisotropy = 137.3699
4 C	Isotropic = 29.5238 Anisotropy = 152.3278
5 N	Isotropic = 64.1762 Anisotropy = 115.0240
6 C	Isotropic = 115.7566 Anisotropy = 31.7774
7 C	Isotropic = 120.9751 Anisotropy = 47.6720
8 C	Isotropic = 12.6442 Anisotropy = 76.6464
9 O	Isotropic = −61.5266 Anisotropy = 520.0462
10 C	Isotropic = 9.7357 Anisotropy = 85.4649
11 O	Isotropic = −29.5188 Anisotropy = 369.1386

12 O	Isotropic =	−22.8805	Anisotropy =	367.5085
2a H	Isotropic =	28.2534	Anisotropy =	12.9010
2b H	Isotropic =	27.9476	Anisotropy =	9.6861
6 H	Isotropic =	26.7442	Anisotropy =	8.0048
7 H	Isotropic =	25.9528	Anisotropy =	4.9888
1” N	Isotropic =	120.6672	Anisotropy =	78.5366
2” C	Isotropic =	13.9316	Anisotropy =	110.4482
3” C	Isotropic =	35.1162	Anisotropy =	132.6147
4” C	Isotropic =	14.8073	Anisotropy =	87.4734
5” N	Isotropic =	−1.5662	Anisotropy =	240.8989
6” C	Isotropic =	−8.3996	Anisotropy =	129.3281
7” S	Isotropic =	89.8102	Anisotropy =	251.1448
8” N	Isotropic =	−19.4933	Anisotropy =	231.4249
1” H	Isotropic =	21.3500	Anisotropy =	16.8446
9” O	Isotropic =	7.3698	Anisotropy =	480.8904
10” N	Isotropic =	−217.4721	Anisotropy =	461.7544
11” O	Isotropic =	58.0944	Anisotropy =	320.9327
11” H	Isotropic =	15.7277	Anisotropy =	18.9028
12” N	Isotropic =	166.5207	Anisotropy =	83.6240
12”a H	Isotropic =	25.9670	Anisotropy =	11.8182
12”b H	Isotropic =	26.3429	Anisotropy =	10.1198
1’ C	Isotropic =	43.4491	Anisotropy =	161.3441
2’ C	Isotropic =	50.8758	Anisotropy =	114.1651
3’ C	Isotropic =	154.0877	Anisotropy =	19.4026
4’ C	Isotropic =	131.6835	Anisotropy =	52.8286
5’ N	Isotropic =	94.4419	Anisotropy =	101.1099
6’ C	Isotropic =	4.4006	Anisotropy =	103.7542
7’ C	Isotropic =	123.5600	Anisotropy =	30.1015
8’ C	Isotropic =	127.3675	Anisotropy =	39.5168
9’ N	Isotropic =	182.2715	Anisotropy =	38.4457
10’ C	Isotropic =	131.0375	Anisotropy =	56.8354
11’ C	Isotropic =	149.7090	Anisotropy =	38.7138
1’ H	Isotropic =	24.2162	Anisotropy =	11.6330
3’a H	Isotropic =	28.8112	Anisotropy =	7.2176
3’b H	Isotropic =	28.9417	Anisotropy =	7.5700
4’a H	Isotropic =	28.4340	Anisotropy =	6.7246
4’b H	Isotropic =	28.2870	Anisotropy =	6.6960
12’ O	Isotropic =	34.0893	Anisotropy =	459.5366
7’ H	Isotropic =	27.8796	Anisotropy =	6.3294
8’a H	Isotropic =	28.2476	Anisotropy =	7.0877
8’b H	Isotropic =	28.5221	Anisotropy =	10.0591
9’a H	Isotropic =	27.1813	Anisotropy =	16.9958
9’b H	Isotropic =	21.6013	Anisotropy =	21.4309
10’a H	Isotropic =	28.6443	Anisotropy =	11.1025
10’b H	Isotropic =	28.0406	Anisotropy =	9.0620
11’a H	Isotropic =	29.2238	Anisotropy =	9.7555
11’b H	Isotropic =	29.8443	Anisotropy =	6.3931

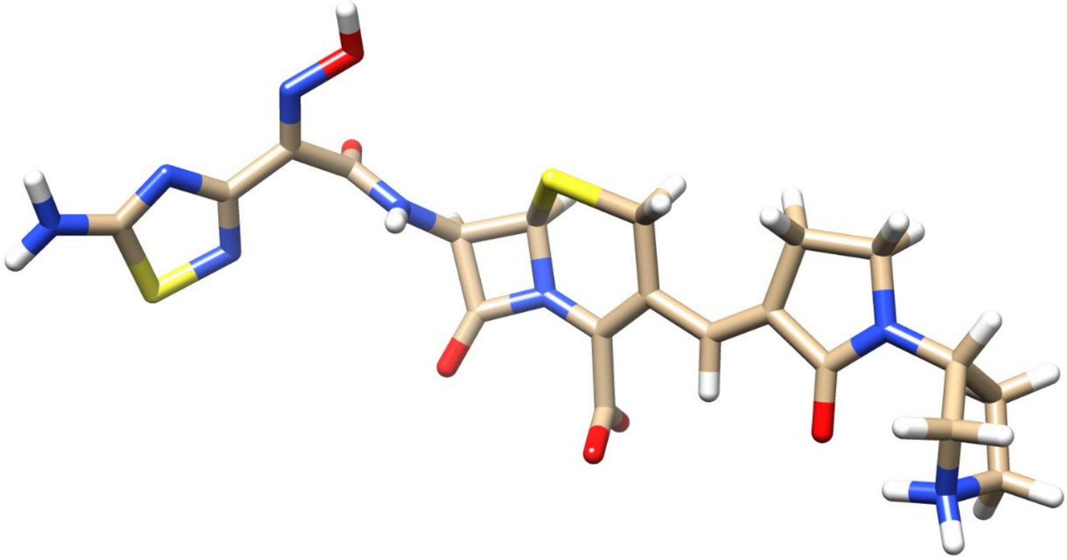

3

Energy = −2461.61407031 Hartree

Atomic coordinates (Å):

	X	Y	Z
1 S	−0.504515	1.901966	−0.233225
2 C	1.310554	1.631245	−0.443284
3 C	1.765788	0.181594	−0.502586
4 C	0.949626	−0.838069	−0.905795
5 N	−0.338619	−0.558470	−1.347623
6 C	−0.922693	0.764800	−1.594062
7 C	−2.327247	0.080231	−1.619867
8 C	−1.554304	−1.222363	−1.278467
9 O	−1.858517	−2.358743	−1.017635
10 C	1.353032	−2.326664	−0.973690
11 O	1.522236	−2.916673	0.115402
12 O	1.466156	−2.775783	−2.137041
2a H	1.747261	2.120516	0.425706
2b H	1.627327	2.187398	−1.328713
6 H	−0.604084	1.192854	−2.544419
7 H	−2.764753	0.072718	−2.617159
1” N	−3.339397	0.484008	−0.685212
2” C	−4.571806	0.897990	−1.074958
3” C	−5.525089	1.229093	0.066947
4” C	−6.415047	0.159349	0.550318
5” N	−7.379084	0.402535	1.487413
6” C	−8.022164	−0.715391	1.741690
7” S	−7.387613	−2.058733	0.782932
8” N	−6.252414	−1.047726	0.053616

1” H	−3.151253	0.409610	0.305295
9” O	−4.931762	1.017608	−2.236137
10” N	−5.627263	2.400741	0.573471
11” O	−4.722697	3.295879	0.015334
11” H	−4.899916	4.123758	0.480542
12” N	−9.017248	−0.837384	2.633769
12”a H	−9.387323	−0.003049	3.064190
12”b H	−9.560558	−1.682362	2.704503
1’ C	3.129998	−0.142144	−0.134587
2’ C	4.182427	0.651551	0.165638
3’ C	4.368796	2.152284	0.228364
4’ C	5.899291	2.339590	0.294981
5’ N	6.407368	1.008989	0.657673
6’ C	5.480037	0.026854	0.502000
7’ C	7.838876	0.785308	0.860598
8’ C	8.150127	−0.146910	2.043825
9’ N	8.268606	−1.495634	1.397385
10’ C	9.041863	−1.263588	0.125914
11’ C	8.549315	0.111971	−0.361171
1’ H	3.353387	−1.204011	−0.098978
3’a H	3.907359	2.566058	1.130036
3’b H	3.947508	2.677115	−0.628220
4’a H	6.201429	3.073336	1.043654
4’b H	6.311292	2.644381	−0.672485
12’ O	5.709326	−1.191301	0.630260
7’ H	8.268241	1.764106	1.062570
8’a H	7.382844	−0.168095	2.813431
8’b H	9.114034	0.100084	2.486043
9’a H	8.691894	−2.200309	2.000871
9’b H	7.291722	−1.762756	1.147310
10’a H	10.099682	−1.258521	0.383002
10’b H	8.841678	−2.079560	−0.564522
11’a H	9.387187	0.716627	−0.704413
11’b H	7.849228	0.010677	−1.189316

SCF GIAO magnetic shielding (ppm):

1 S	Isotropic = 491.4481 Anisotropy = 226.8512
2 C	Isotropic = 148.8812 Anisotropy = 20.7783
3 C	Isotropic = 67.4316 Anisotropy = 136.9499
4 C	Isotropic = 30.0586 Anisotropy = 151.2963
5 N	Isotropic = 64.5590 Anisotropy = 117.4091
6 C	Isotropic = 116.0985 Anisotropy = 31.5969
7 C	Isotropic = 120.8228 Anisotropy = 47.9201
8 C	Isotropic = 12.8433 Anisotropy = 75.9823
9 O	Isotropic = −60.2119 Anisotropy = 517.9127
10 C	Isotropic = 9.8656 Anisotropy = 85.4654

11 O	Isotropic =	−28.3580	Anisotropy =	368.5603
12 O	Isotropic =	−24.1978	Anisotropy =	367.3086
2a H	Isotropic =	28.3268	Anisotropy =	12.9852
2b H	Isotropic =	28.0245	Anisotropy =	9.5044
6 H	Isotropic =	26.7582	Anisotropy =	7.3571
7 H	Isotropic =	25.8996	Anisotropy =	4.8237
1” N	Isotropic =	124.2744	Anisotropy =	109.3054
2” C	Isotropic =	13.4795	Anisotropy =	113.3740
3” C	Isotropic =	24.9286	Anisotropy =	117.1380
4” C	Isotropic =	12.9411	Anisotropy =	83.8874
5” N	Isotropic =	−2.6788	Anisotropy =	260.1539
6” C	Isotropic =	−8.4199	Anisotropy =	124.6655
7” S	Isotropic =	75.7317	Anisotropy =	247.8337
8” N	Isotropic =	−38.8357	Anisotropy =	254.4857
1” H	Isotropic =	25.9118	Anisotropy =	8.0976
9” O	Isotropic =	−61.5584	Anisotropy =	590.5385
10” N	Isotropic =	−156.8903	Anisotropy =	369.6034
11” O	Isotropic =	95.1517	Anisotropy =	143.7746
11” H	Isotropic =	24.5105	Anisotropy =	8.7366
12” N	Isotropic =	167.9878	Anisotropy =	88.4956
12”a H	Isotropic =	26.2768	Anisotropy =	12.1918
12”b H	Isotropic =	26.5706	Anisotropy =	10.1290
1’ C	Isotropic =	43.6856	Anisotropy =	161.3749
2’ C	Isotropic =	50.7490	Anisotropy =	113.4140
3’ C	Isotropic =	154.3233	Anisotropy =	18.6734
4’ C	Isotropic =	131.0330	Anisotropy =	53.3682
5’ N	Isotropic =	93.7034	Anisotropy =	100.8870
6’ C	Isotropic =	4.1822	Anisotropy =	103.8056
7’ C	Isotropic =	123.8229	Anisotropy =	29.1613
8’ C	Isotropic =	127.0386	Anisotropy =	38.4023
9’ N	Isotropic =	182.3691	Anisotropy =	38.9727
10’ C	Isotropic =	132.1254	Anisotropy =	55.6498
11’ C	Isotropic =	149.5216	Anisotropy =	39.5246
1’ H	Isotropic =	24.2627	Anisotropy =	11.7150
3’a H	Isotropic =	28.8272	Anisotropy =	7.1954
3’b H	Isotropic =	28.9444	Anisotropy =	7.5805
4’a H	Isotropic =	28.3956	Anisotropy =	6.7865
4’b H	Isotropic =	28.2795	Anisotropy =	6.7493
12’ O	Isotropic =	34.5148	Anisotropy =	455.9602
7’ H	Isotropic =	27.9883	Anisotropy =	6.1409
8’a H	Isotropic =	28.2441	Anisotropy =	7.0511
8’b H	Isotropic =	28.4852	Anisotropy =	10.0919
9’a H	Isotropic =	27.1614	Anisotropy =	17.0515
9’b H	Isotropic =	21.4601	Anisotropy =	21.8347
10’a H	Isotropic =	28.6407	Anisotropy =	11.0776
10’b H	Isotropic =	28.0673	Anisotropy =	9.1725
11’a H	Isotropic =	29.2612	Anisotropy =	9.7212
11’b H	Isotropic =	29.8560	Anisotropy =	6.2943

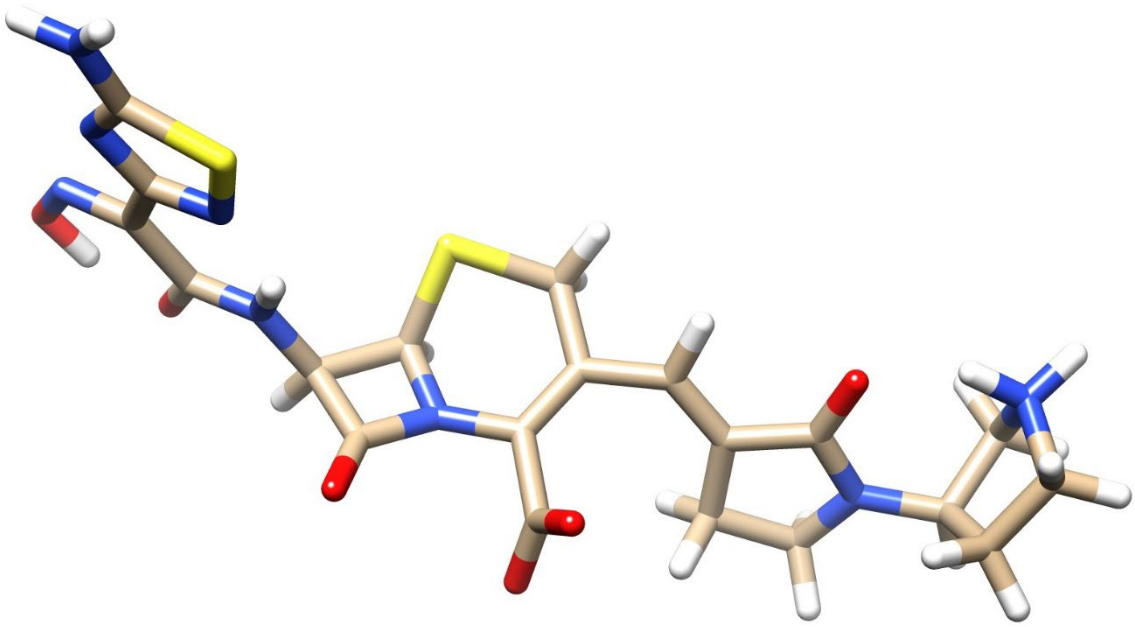

4

Energy = −2461.61356987 Hartree

Atomic coordinates (Å):

	X	Y	Z
1 S	−1.145882	0.035705	−1.728032
2 C	0.696152	0.080501	−1.783798
3 C	1.424722	−0.207136	−0.477268
4 C	0.884889	−0.901309	0.554812
5 N	−0.393337	−1.457301	0.404375
6 C	−1.170403	−1.556018	−0.833827
7 C	−2.421522	−1.885982	0.044037
8 C	−1.481266	−1.607654	1.245531
9 O	−1.627371	−1.512587	2.439121
10 C	1.532462	−1.144544	1.933097
11 O	1.890998	−0.133377	2.576327
12 O	1.620737	−2.350535	2.264887
2a H	0.942396	1.090248	−2.118758
2b H	1.030212	−0.604998	−2.567365
6 H	−0.831132	−2.358227	−1.488908
7 H	−2.709868	−2.932422	−0.041720
1” N	−3.593522	−1.062175	−0.039683
2” C	−4.770570	−1.532132	−0.489001
3” C	−5.960578	−0.605341	−0.501014
4” C	−5.901764	0.807348	−0.037638
5” N	−7.022826	1.586215	−0.061484
6” C	−6.733035	2.785810	0.390916
7” S	−5.031977	2.919715	0.847538
8” N	−4.768689	1.315406	0.399705

1” H	−3.577407	−0.085267	0.268017
9” O	−4.909634	−2.703435	−0.892679
10” N	−7.130719	−0.981648	−0.921844
11” O	−7.313690	−2.242562	−1.365903
11” H	−6.435861	−2.721268	−1.286969
12” N	−7.609343	3.795582	0.480927
12”a H	−8.578468	3.624367	0.259011
12”b H	−7.365591	4.677342	0.901017
1’ C	2.774681	0.364418	−0.465649
2’ C	3.940471	−0.236261	−0.178940
3’ C	4.257313	−1.653632	0.220812
4’ C	5.775365	−1.785055	−0.023740
5’ N	6.234892	−0.393985	−0.166445
6’ C	5.223405	0.497391	−0.307339
7’ C	7.658000	−0.059166	−0.221165
8’ C	8.063561	0.727493	−1.481909
9’ N	7.991141	2.157996	−1.032759
10’ C	8.597973	2.169949	0.346051
11’ C	8.118750	0.846791	0.967839
1’ H	2.849591	1.392601	−0.817876
3’a H	3.681242	−2.386037	−0.346949
3’b H	4.030595	−1.816174	1.276579
4’a H	5.999843	−2.341887	−0.938611
4’b H	6.293011	−2.265953	0.807528
12’ O	5.369012	1.714655	−0.523554
7’ H	8.190505	−1.007376	−0.211053
8’a H	7.414016	0.572364	−2.339718
8’b H	9.096180	0.515078	−1.754742
9’a H	8.442730	2.808377	−1.675605
9’b H	6.975160	2.371890	−0.949144
10’a H	9.679118	2.205977	0.224010
10’b H	8.263235	3.059310	0.874610
11’a H	8.923965	0.373924	1.527523
11’b H	7.287036	1.010694	1.651064

SCF GIAO magnetic shielding (ppm):

1 S	Isotropic = 494.9116 Anisotropy = 200.0665
2 C	Isotropic = 147.3289 Anisotropy = 30.0550
3 C	Isotropic = 64.4859 Anisotropy = 132.0036
4 C	Isotropic = 37.1744 Anisotropy = 143.4482
5 N	Isotropic = 67.8810 Anisotropy = 120.9855
6 C	Isotropic = 116.0039 Anisotropy = 30.9968
7 C	Isotropic = 122.6054 Anisotropy = 45.5738
8 C	Isotropic = 12.6729 Anisotropy = 79.0964
9 O	Isotropic = −52.1498 Anisotropy = 509.7231
10 C	Isotropic = 8.8022 Anisotropy = 90.5053

11 O	Isotropic = −16.2772 Anisotropy = 354.1334
12 O	Isotropic = −5.6731 Anisotropy = 354.2198
2a H	Isotropic = 28.6243 Anisotropy = 9.0673
2b H	Isotropic = 28.3415 Anisotropy = 9.0679
6 H	Isotropic = 26.6883 Anisotropy = 8.0016
7 H	Isotropic = 26.0343 Anisotropy = 5.0437
1” N	Isotropic = 118.1378 Anisotropy = 76.7817
2” C	Isotropic = 13.1389 Anisotropy = 106.9639
3” C	Isotropic = 35.7518 Anisotropy = 134.7492
4” C	Isotropic = 11.1780 Anisotropy = 82.2275
5” N	Isotropic = −2.9150 Anisotropy = 253.9290
6” C	Isotropic = −6.4906 Anisotropy = 124.3722
7” S	Isotropic = 83.7885 Anisotropy = 272.2541
8” N	Isotropic = −11.9264 Anisotropy = 225.9223
1” H	Isotropic = 21.0463 Anisotropy = 17.8504
9” O	Isotropic = 10.8423 Anisotropy = 464.5322
10” N	Isotropic = −231.2427 Anisotropy = 476.3096
11” O	Isotropic = 51.5407 Anisotropy = 332.9116
11” H	Isotropic = 15.4585 Anisotropy = 20.4789
12” N	Isotropic = 167.4345 Anisotropy = 88.4540
12”a H	Isotropic = 26.1478 Anisotropy = 12.5833
12”b H	Isotropic = 26.4999 Anisotropy = 9.9937
1’ C	Isotropic = 44.8340 Anisotropy = 144.7938
2’ C	Isotropic = 41.2091 Anisotropy = 124.3284
3’ C	Isotropic = 153.9926 Anisotropy = 19.3759
4’ C	Isotropic = 130.4147 Anisotropy = 54.0730
5’ N	Isotropic = 89.2361 Anisotropy = 108.4940
6’ C	Isotropic = 5.4041 Anisotropy = 102.8672
7’ C	Isotropic = 123.9059 Anisotropy = 29.9146
8’ C	Isotropic = 126.6481 Anisotropy = 36.9345
9’ N	Isotropic = 181.4247 Anisotropy = 38.0123
10’ C	Isotropic = 132.0035 Anisotropy = 55.0615
11’ C	Isotropic = 149.7386 Anisotropy = 38.2444
1’ H	Isotropic = 24.9291 Anisotropy = 8.5423
3’a H	Isotropic = 29.4565 Anisotropy = 5.8886
3’b H	Isotropic = 28.9003 Anisotropy = 10.0165
4’a H	Isotropic = 28.2805 Anisotropy = 7.1863
4’b H	Isotropic = 28.3140 Anisotropy = 5.9147
12’ O	Isotropic = 30.7948 Anisotropy = 459.8555
7’ H	Isotropic = 27.8417 Anisotropy = 6.2805
8’a H	Isotropic = 28.2907 Anisotropy = 6.8965
8’b H	Isotropic = 28.5377 Anisotropy = 10.0798
9’a H	Isotropic = 27.0288 Anisotropy = 16.9411
9’b H	Isotropic = 21.8792 Anisotropy = 20.7790
10’a H	Isotropic = 28.6791 Anisotropy = 11.0549
10’b H	Isotropic = 28.0017 Anisotropy = 9.4254
11’a H	Isotropic = 29.1209 Anisotropy = 9.6596
11’b H	Isotropic = 29.7031 Anisotropy = 6.4221

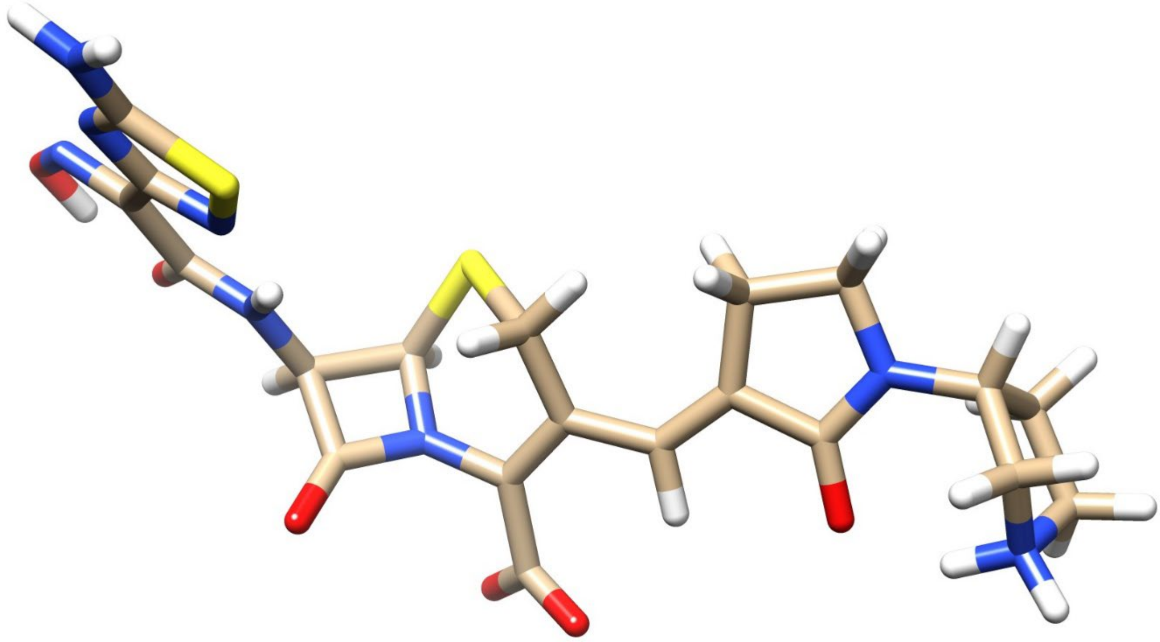

5

Energy = −2461.61249954 Hartree

Atomic coordinates (Å):

	X	Y	Z
1 S	0.737157	−0.215406	1.683264
2 C	−0.345548	0.287689	0.255902
3 C	−1.328261	−0.771413	−0.196934
4 C	−0.807499	−1.975135	−0.577379
5 N	0.554019	−2.145824	−0.256671
6 C	1.203842	−1.863340	1.033859
7 C	2.578724	−2.140765	0.316824
8 C	1.714029	−2.369931	−0.954103
9 O	1.937339	−2.627845	−2.114315
10 C	−1.528078	−3.195166	−1.179919
11 O	−2.436991	−2.970536	−2.013499
12 O	−1.113883	−4.304482	−0.766522
2a H	0.312449	0.550574	−0.574065
2b H	−0.827568	1.197378	0.594088
6 H	0.965441	−2.604594	1.796621
7 H	3.052481	−3.051624	0.678899
1” N	3.561243	−1.101265	0.236031
2” C	4.791305	−1.229787	0.761169
3” C	5.765694	−0.088345	0.606017
4” C	5.440466	1.176390	−0.107510
5” N	6.392330	2.143680	−0.259684
6” C	5.882545	3.162047	−0.915747
7” S	4.189936	2.885427	−1.339140
8” N	4.233811	1.372123	−0.596068

1” H	3.350392	−0.208765	−0.218737
9” O	5.156719	−2.258387	1.365268
10” N	6.971570	−0.133334	1.087549
11” O	7.391156	−1.230049	1.752669
11” H	6.631906	−1.886482	1.759776
12” N	6.562627	4.268937	−1.245679
12”a H	6.116984	5.061087	−1.678627
12”b H	7.509112	4.377836	−0.913751
1’ C	−2.757459	−0.524266	−0.277721
2’ C	−3.502779	0.504971	0.175551
3’ C	−3.180857	1.769588	0.939558
4’ C	−4.557873	2.368050	1.303191
5’ N	−5.515714	1.582576	0.514191
6’ C	−4.967694	0.486536	−0.067160
7’ C	−6.948605	1.876005	0.561891
8’ C	−7.629414	1.818780	−0.817182
9’ N	−8.175642	0.422886	−0.876077
10’ C	−8.747189	0.161636	0.492886
11’ C	−7.763742	0.869955	1.441636
1’ H	−3.310486	−1.303731	−0.789837
3’a H	−2.620630	2.473487	0.317830
3’b H	−2.593450	1.581015	1.838768
4’a H	−4.637348	3.424575	1.042281
4’b H	−4.777383	2.260119	2.369341
12’ O	−5.602930	−0.374434	−0.702006
7’ H	−7.033739	2.886984	0.954340
8’a H	−6.962372	1.993806	−1.657149
8’b H	−8.469556	2.510014	−0.860395
9’a H	−8.850796	0.283801	−1.627579
9’b H	−7.343487	−0.188321	−1.015785
10’a H	−9.742057	0.603111	0.519495
10’b H	−8.822865	−0.912115	0.647651
11’a H	−8.303236	1.390508	2.231139
11’b H	−7.087886	0.157397	1.912837

SCF GIAO magnetic shielding (ppm):

1 S	Isotropic = 426.9708 Anisotropy = 370.9540
2 C	Isotropic = 150.4460 Anisotropy = 18.1561
3 C	Isotropic = 40.4666 Anisotropy = 170.1558
4 C	Isotropic = 28.1567 Anisotropy = 138.5591
5 N	Isotropic = 60.6145 Anisotropy = 92.9093
6 C	Isotropic = 113.5719 Anisotropy = 33.7797
7 C	Isotropic = 118.8495 Anisotropy = 50.2065
8 C	Isotropic = 11.5598 Anisotropy = 84.7274
9 O	Isotropic = −36.7417 Anisotropy = 476.9201
10 C	Isotropic = 10.4253 Anisotropy = 80.5935
11 O	Isotropic = −19.6669 Anisotropy = 361.3085

12 O	Isotropic = −12.0486 Anisotropy = 355.1422
2a H	Isotropic = 28.5128 Anisotropy = 8.9371
2b H	Isotropic = 28.4749 Anisotropy = 14.4765
6 H	Isotropic = 26.9407 Anisotropy = 8.5619
7 H	Isotropic = 26.1949 Anisotropy = 5.6155
1” N	Isotropic = 113.8704 Anisotropy = 79.2638
2” C	Isotropic = 13.1230 Anisotropy = 106.2430
3” C	Isotropic = 36.3014 Anisotropy = 135.8678
4” C	Isotropic = 11.5413 Anisotropy = 82.4413
5” N	Isotropic = −2.9691 Anisotropy = 255.3843
6” C	Isotropic = −6.1279 Anisotropy = 125.5095
7” S	Isotropic = 82.2764 Anisotropy = 278.2584
8” N	Isotropic = −10.3400 Anisotropy = 227.6786
1” H	Isotropic = 21.0042 Anisotropy = 17.0501
9” O	Isotropic = 14.1750 Anisotropy = 452.8427
10” N	Isotropic = −230.3635 Anisotropy = 476.0496
11” O	Isotropic = 51.8115 Anisotropy = 336.6939
11” H	Isotropic = 15.2707 Anisotropy = 20.3201
12” N	Isotropic = 167.6879 Anisotropy = 88.2885
12”a H	Isotropic = 26.5091 Anisotropy = 9.9114
12”b H	Isotropic = 26.1354 Anisotropy = 12.6558
1’ C	Isotropic = 47.4796 Anisotropy = 167.5536
2’ C	Isotropic = 45.2106 Anisotropy = 119.1011
3’ C	Isotropic = 155.3659 Anisotropy = 16.7705
4’ C	Isotropic = 131.4389 Anisotropy = 54.4313
5’ N	Isotropic = 90.9405 Anisotropy = 99.4083
6’ C	Isotropic = 4.6645 Anisotropy = 101.6692
7’ C	Isotropic = 123.6662 Anisotropy = 30.0686
8’ C	Isotropic = 127.2454 Anisotropy = 39.4658
9’ N	Isotropic = 182.8444 Anisotropy = 39.3006
10’ C	Isotropic = 130.9589 Anisotropy = 56.3042
11’ C	Isotropic = 149.7800 Anisotropy = 38.5858
1’ H	Isotropic = 23.0671 Anisotropy = 11.8049
3’a H	Isotropic = 28.8175 Anisotropy = 7.3266
3’b H	Isotropic = 28.9641 Anisotropy = 8.0247
4’a H	Isotropic = 28.3283 Anisotropy = 6.8018
4’b H	Isotropic = 28.2111 Anisotropy = 6.4970
12’ O	Isotropic = 32.1211 Anisotropy = 471.4117
7’ H	Isotropic = 27.8602 Anisotropy = 6.1622
8’a H	Isotropic = 28.2885 Anisotropy = 7.0044
8’b H	Isotropic = 28.5025 Anisotropy = 9.8120
9’a H	Isotropic = 27.2436 Anisotropy = 17.0052
9’b H	Isotropic = 21.7985 Anisotropy = 21.0315
10’a H	Isotropic = 28.6996 Anisotropy = 11.0010
10’b H	Isotropic = 28.0207 Anisotropy = 9.2730
11’a H	Isotropic = 29.1881 Anisotropy = 9.7444
11’b H	Isotropic = 29.7972 Anisotropy = 6.2702

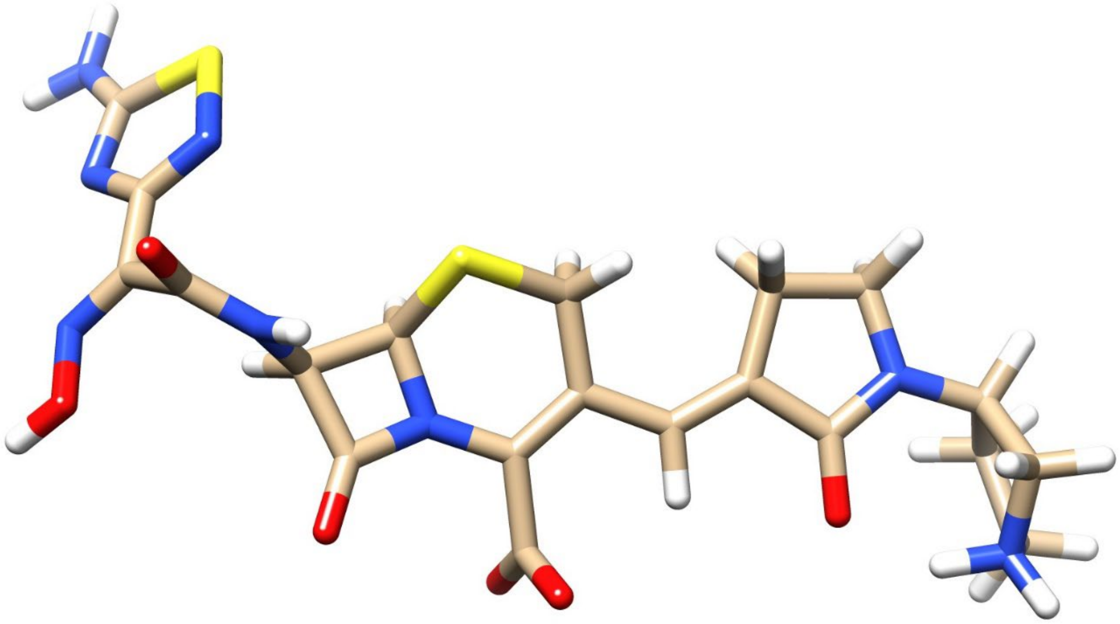

6

Energy = −2461.61130232 Hartree

Atomic coordinates (Å):

	X	Y	Z
1 S	−0.940936	−0.697438	−1.380773
2 C	0.748412	−0.947577	−0.679425
3 C	1.429831	0.290751	−0.118915
4 C	0.738130	1.376900	0.339578
5 N	−0.652525	1.349890	0.364368
6 C	−1.519273	0.197076	0.097428
7 C	−2.715955	1.195937	−0.032698
8 C	−1.643926	2.301709	0.184430
9 O	−1.639310	3.506227	0.171202
10 C	1.372738	2.664176	0.907868
11 O	1.982167	3.398475	0.100190
12 O	1.197131	2.823342	2.137476
2a H	1.321096	−1.336196	−1.519525
2b H	0.681758	−1.740612	0.069054
6 H	−1.580996	−0.486588	0.943893
7 H	−3.399620	1.115720	0.810887
1” N	−3.457163	1.265497	−1.259287
2” C	−4.784863	1.039248	−1.421418
3” C	−5.602324	0.699192	−0.179632
4” C	−5.787064	−0.724347	0.149556
5” N	−6.634793	−1.124373	1.142514
6” C	−6.597524	−2.435488	1.236036
7” S	−5.482093	−3.136111	0.055930
8” N	−5.094621	−1.606576	−0.538167

1” H	−2.956904	1.484080	−2.113683
9” O	−5.344384	1.107133	−2.506420
10” N	−6.198187	1.585382	0.527606
11” O	−5.911899	2.875274	0.099099
11” H	−6.406101	3.437973	0.709466
12” N	−7.296374	−3.155168	2.126384
12”a H	−7.977018	−2.685487	2.704519
12”b H	−7.317867	−4.161409	2.095636
1’ C	2.877682	0.322728	−0.047295
2’ C	3.802298	−0.628119	−0.309066
3’ C	3.711671	−2.070209	−0.760157
4’ C	5.137967	−2.623577	−0.554268
5’ N	5.966430	−1.423266	−0.375357
6’ C	5.235644	−0.300512	−0.145289
7’ C	7.413378	−1.532059	−0.191350
8’ C	8.208000	−0.453243	−0.947340
9’ N	8.403711	0.618236	0.084418
10’ C	8.738882	−0.110456	1.359251
11’ C	7.870329	−1.380307	1.298217
1’ H	3.297912	1.270433	0.274876
3’a H	3.440512	−2.133160	−1.818075
3’b H	2.982777	−2.652975	−0.197994
4’a H	5.492656	−3.199717	−1.410229
4’b H	5.201339	−3.256613	0.336612
12’ O	5.717460	0.810496	0.148264
7’ H	7.691629	−2.508923	−0.580636
8’a H	7.698228	−0.044884	−1.816128
8’b H	9.189634	−0.827617	−1.233939
9’a H	9.104498	1.311152	−0.177601
9’b H	7.467695	1.064717	0.192262
10’a H	9.804130	−0.333759	1.337202
10’b H	8.522802	0.537794	2.205369
11’a H	8.442115	−2.249088	1.620274
11’b H	6.999327	−1.293933	1.946338

SCF GIAO magnetic shielding (ppm):

1 S	Isotropic = 494.8350 Anisotropy = 225.1217
2 C	Isotropic = 148.8991 Anisotropy = 20.4686
3 C	Isotropic = 66.4814 Anisotropy = 138.2178
4 C	Isotropic = 30.1920 Anisotropy = 151.2697
5 N	Isotropic = 66.1075 Anisotropy = 116.1641
6 C	Isotropic = 116.1652 Anisotropy = 30.3841
7 C	Isotropic = 116.2413 Anisotropy = 31.0066
8 C	Isotropic = 13.6978 Anisotropy = 75.5819
9 O	Isotropic = −58.3173 Anisotropy = 516.8222
10 C	Isotropic = 9.6173 Anisotropy = 85.5997
11 O	Isotropic = −28.7745 Anisotropy = 367.6597

12 O	Isotropic = −23.4965 Anisotropy = 366.4635
2a H	Isotropic = 28.2747 Anisotropy = 13.4614
2b H	Isotropic = 28.0514 Anisotropy = 9.1923
6 H	Isotropic = 27.0364 Anisotropy = 6.6845
7 H	Isotropic = 27.0655 Anisotropy = 6.2837
1” N	Isotropic = 123.5336 Anisotropy = 120.6555
2” C	Isotropic = 11.4504 Anisotropy = 106.7113
3” C	Isotropic = 26.6703 Anisotropy = 116.0408
4” C	Isotropic = 13.9417 Anisotropy = 84.6008
5” N	Isotropic = −1.2648 Anisotropy = 259.5438
6” C	Isotropic = −8.1633 Anisotropy = 125.8950
7” S	Isotropic = 78.5127 Anisotropy = 245.8424
8” N	Isotropic = −34.6630 Anisotropy = 259.9654
1” H	Isotropic = 25.9496 Anisotropy = 5.2006
9” O	Isotropic = −70.8987 Anisotropy = 542.2505
10” N	Isotropic = −159.0226 Anisotropy = 374.7139
11” O	Isotropic = 93.4716 Anisotropy = 143.3921
11” H	Isotropic = 24.3859 Anisotropy = 9.2626
12” N	Isotropic = 168.0536 Anisotropy = 88.6539
12”a H	Isotropic = 26.1530 Anisotropy = 12.1575
12”b H	Isotropic = 26.5303 Anisotropy = 10.0149
1’ C	Isotropic = 43.8741 Anisotropy = 161.0771
2’ C	Isotropic = 50.4899 Anisotropy = 113.3062
3’ C	Isotropic = 153.3685 Anisotropy = 19.6727
4’ C	Isotropic = 131.2128 Anisotropy = 53.5535
5’ N	Isotropic = 93.9050 Anisotropy = 100.2735
6’ C	Isotropic = 4.1765 Anisotropy = 103.5601
7’ C	Isotropic = 124.0298 Anisotropy = 29.1399
8’ C	Isotropic = 126.8328 Anisotropy = 39.1977
9’ N	Isotropic = 182.5964 Anisotropy = 39.3767
10’ C	Isotropic = 132.1363 Anisotropy = 56.0824
11’ C	Isotropic = 149.9737 Anisotropy = 37.4998
1’ H	Isotropic = 24.2053 Anisotropy = 11.7064
3’a H	Isotropic = 28.7830 Anisotropy = 7.1532
3’b H	Isotropic = 28.9309 Anisotropy = 7.9615
4’a H	Isotropic = 28.4197 Anisotropy = 6.7607
4’b H	Isotropic = 28.2625 Anisotropy = 6.4678
12’ O	Isotropic = 36.2532 Anisotropy = 462.7575
7’ H	Isotropic = 27.8848 Anisotropy = 6.3679
8’a H	Isotropic = 28.3179 Anisotropy = 7.0190
8’b H	Isotropic = 28.5530 Anisotropy = 9.9562
9’a H	Isotropic = 27.2757 Anisotropy = 16.9601
9’b H	Isotropic = 21.6804 Anisotropy = 21.4462
10’a H	Isotropic = 28.7721 Anisotropy = 10.9705
10’b H	Isotropic = 28.1039 Anisotropy = 9.2142
11’a H	Isotropic = 29.1915 Anisotropy = 9.7606
11’b H	Isotropic = 29.8733 Anisotropy = 6.3358

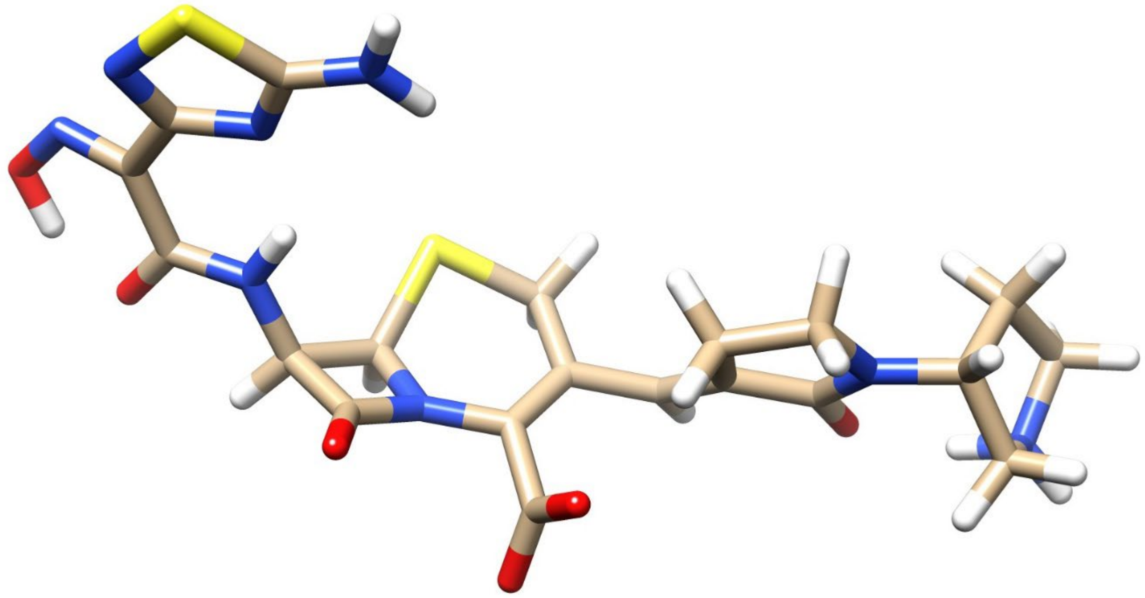

7

Energy = −2461.60871769 Hartree

Atomic coordinates (Å):

	X	Y	Z
1 S	−1.100389	−0.712338	−1.860580
2 C	0.714633	−1.038344	−1.886212
3 C	1.381238	−1.257690	−0.533579
4 C	0.741698	−1.684086	0.575013
5 N	−0.636326	−1.957506	0.501923
6 C	−1.440612	−2.091065	−0.712574
7 C	−2.713881	−2.022784	0.193848
8 C	−1.705090	−1.793998	1.353681
9 O	−1.795854	−1.548608	2.533292
10 C	1.403714	−2.064731	1.916051
11 O	1.836772	−1.140354	2.637910
12 O	1.419784	−3.299000	2.139624
2a H	1.141993	−0.154121	−2.363007
2b H	0.907817	−1.886601	−2.550145
6 H	−1.285708	−3.042511	−1.221491
7 H	−3.225111	−2.982190	0.250555
1” N	−3.683662	−0.984486	−0.011474
2” C	−4.944393	−1.246529	−0.403194
3” C	−5.892378	−0.086600	−0.564013
4” C	−5.566278	1.292176	−0.102720
5” N	−4.257412	1.696489	−0.049236
6” C	−4.192728	2.948562	0.360840
7” S	−5.802098	3.587593	0.671008
8” N	−6.522297	2.118692	0.246326

1” H	−3.428884	0.001786	0.087257
9” O	−5.348784	−2.400648	−0.638614
10” N	−7.064646	−0.209484	−1.104592
11” O	−7.494515	−1.427462	−1.506368
11” H	−6.791682	−2.093901	−1.256439
12” N	−3.056127	3.636648	0.517922
12”a H	−2.175494	3.199499	0.292367
12”b H	−3.050517	4.595638	0.823814
1’ C	2.850128	−1.088507	−0.603812
2’ C	3.573667	−0.102482	−0.061851
3’ C	3.147010	1.068863	0.782814
4’ C	4.410395	1.949193	0.891453
5’ N	5.485305	1.118621	0.321897
6’ C	5.042034	−0.015295	−0.272025
7’ C	6.860665	1.614241	0.253428
8’ C	7.910662	0.567632	0.665349
9’ N	8.330611	−0.035559	−0.643443
10’ C	8.444842	1.124798	−1.597724
11’ C	7.290374	2.055198	−1.185462
1’ H	3.385970	−1.796281	−1.236994
3’a H	2.813930	0.711937	1.758764
3’b H	2.314519	1.611761	0.331201
4’a H	4.654894	2.209488	1.923090
4’b H	4.319384	2.875686	0.317190
12’ O	5.755729	−0.830469	−0.883158
7’ H	6.910629	2.453043	0.944000
8’a H	7.538431	−0.207430	1.330192
8’b H	8.781545	1.049184	1.107311
9’a H	9.186390	−0.586792	−0.578652
9’b H	7.531885	−0.635556	−0.937625
10’a H	9.421620	1.579127	−1.440389
10’b H	8.377796	0.754079	−2.617774
11’a H	7.616079	3.094019	−1.194331
11’b H	6.446946	1.963172	−1.868372

SCF GIAO magnetic shielding (ppm):

1 S	Isotropic = 479.4611 Anisotropy = 244.0692
2 C	Isotropic = 148.1172 Anisotropy = 25.5302
3 C	Isotropic = 66.4439 Anisotropy = 126.7827
4 C	Isotropic = 42.1735 Anisotropy = 136.3774
5 N	Isotropic = 72.9992 Anisotropy = 116.7399
6 C	Isotropic = 117.5587 Anisotropy = 31.5426
7 C	Isotropic = 121.5645 Anisotropy = 47.0792
8 C	Isotropic = 13.4876 Anisotropy = 78.9343
9 O	Isotropic = −41.9734 Anisotropy = 492.9038
10 C	Isotropic = 9.0733 Anisotropy = 87.7627
11 O	Isotropic = −21.7730 Anisotropy = 371.9045

12 O	Isotropic =	−13.7238	Anisotropy =	332.9174
2a H	Isotropic =	28.9806	Anisotropy =	8.3697
2b H	Isotropic =	28.1433	Anisotropy =	9.8738
6 H	Isotropic =	26.8247	Anisotropy =	8.5582
7 H	Isotropic =	26.0936	Anisotropy =	5.0070
1” N	Isotropic =	120.0142	Anisotropy =	79.2961
2” C	Isotropic =	13.4893	Anisotropy =	108.0560
3” C	Isotropic =	34.7901	Anisotropy =	131.8644
4” C	Isotropic =	14.8222	Anisotropy =	87.7343
5” N	Isotropic =	−0.8230	Anisotropy =	242.1557
6” C	Isotropic =	−7.8676	Anisotropy =	130.3829
7” S	Isotropic =	96.3120	Anisotropy =	241.8966
8” N	Isotropic =	−19.0944	Anisotropy =	232.8672
1” H	Isotropic =	21.4654	Anisotropy =	16.5336
9” O	Isotropic =	8.1282	Anisotropy =	479.4288
10” N	Isotropic =	−215.5231	Anisotropy =	458.2984
11” O	Isotropic =	59.5442	Anisotropy =	319.9941
11” H	Isotropic =	15.7174	Anisotropy =	18.7220
12” N	Isotropic =	166.5917	Anisotropy =	83.8777
12”a H	Isotropic =	26.0626	Anisotropy =	12.0125
12”b H	Isotropic =	26.4182	Anisotropy =	9.9243
1’ C	Isotropic =	44.1237	Anisotropy =	154.1897
2’ C	Isotropic =	38.3126	Anisotropy =	128.0398
3’ C	Isotropic =	155.5377	Anisotropy =	21.9070
4’ C	Isotropic =	131.3017	Anisotropy =	55.5811
5’ N	Isotropic =	86.7259	Anisotropy =	99.3324
6’ C	Isotropic =	5.6652	Anisotropy =	103.4679
7’ C	Isotropic =	123.0868	Anisotropy =	31.4334
8’ C	Isotropic =	127.1983	Anisotropy =	41.3725
9’ N	Isotropic =	182.0401	Anisotropy =	39.3824
10’ C	Isotropic =	131.7657	Anisotropy =	56.2807
11’ C	Isotropic =	149.3479	Anisotropy =	38.7326
1’ H	Isotropic =	24.6561	Anisotropy =	7.5343
3’a H	Isotropic =	27.5398	Anisotropy =	10.9087
3’b H	Isotropic =	29.2983	Anisotropy =	7.4582
4’a H	Isotropic =	28.3744	Anisotropy =	6.4119
4’b H	Isotropic =	28.2533	Anisotropy =	6.4786
12’ O	Isotropic =	34.3416	Anisotropy =	474.7919
7’ H	Isotropic =	27.8862	Anisotropy =	5.9639
8’a H	Isotropic =	28.2759	Anisotropy =	6.9700
8’b H	Isotropic =	28.5230	Anisotropy =	9.8834
9’a H	Isotropic =	27.2342	Anisotropy =	17.0810
9’b H	Isotropic =	21.9864	Anisotropy =	20.3766
10’a H	Isotropic =	28.7172	Anisotropy =	11.0038
10’b H	Isotropic =	28.0547	Anisotropy =	9.3038
11’a H	Isotropic =	29.1541	Anisotropy =	9.5433
11’b H	Isotropic =	29.8303	Anisotropy =	6.4619

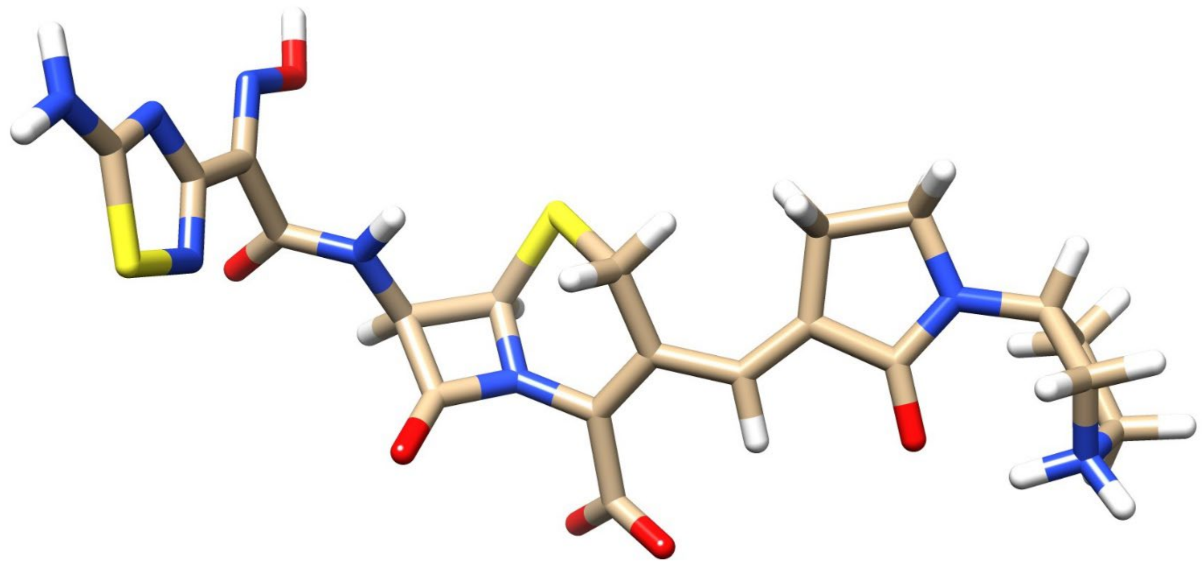

8

Energy = −2461.60822865 Hartree

Atomic coordinates (Å):

	X	Y	Z
1 S	−0.334295	−0.462147	1.822961
2 C	0.649868	−0.618626	0.250824
3 C	1.556339	0.554223	−0.058981
4 C	0.969656	1.783080	−0.152844
5 N	−0.357352	1.832424	0.318933
6 C	−0.865966	1.277971	1.582289
7 C	−2.307987	1.639918	1.082203
8 C	−1.588825	2.143069	−0.205006
9 O	−1.933332	2.611367	−1.264813
10 C	1.597871	3.124005	−0.575899
11 O	2.414002	3.097373	−1.526938
12 O	1.211216	4.116605	0.085551
2a H	−0.062292	−0.747391	−0.565618
2b H	1.188658	−1.552103	0.365543
6 H	−0.552034	1.850228	2.454530
7 H	−2.764812	2.451212	1.647537
1” N	−3.260105	0.576080	0.943579
2” C	−4.577860	0.724303	1.214494
3” C	−5.409118	−0.543982	1.061791
4” C	−6.087658	−0.773644	−0.225503
5” N	−6.925685	−1.837429	−0.409169
6” C	−7.388514	−1.812925	−1.639084
7” S	−6.749543	−0.433444	−2.542154

8” N	−5.859584	0.075545	−1.203693
1” H	−2.912665	−0.341053	0.692544
9” O	−5.106793	1.769543	1.567351
10” N	−5.593578	−1.377272	2.016760
11” O	−4.890528	−1.027313	3.164029
11” H	−5.114147	−1.725398	3.793134
12” N	−8.216679	−2.731903	−2.160595
12”a H	−8.645972	−2.603610	−3.062548
12”b H	−8.615927	−3.426085	−1.546454
1’ C	2.979452	0.391717	−0.304133
2’ C	3.798637	−0.652500	−0.063833
3’ C	3.590636	−2.017085	0.552921
4’ C	5.017552	−2.565897	0.772744
5’ N	5.879679	−1.646142	0.018601
6’ C	5.238299	−0.529013	−0.406307
7’ C	7.328345	−1.844259	−0.029349
8’ C	7.930514	−1.631483	−1.429484
9’ N	8.384891	−0.201495	−1.397929
10’ C	9.008202	−0.013095	−0.039663
11’ C	8.122524	−0.858811	0.891970
1’ H	3.453927	1.263551	−0.740912
3’a H	3.039981	−2.674704	−0.125859
3’b H	3.042688	−1.977066	1.494671
4’a H	5.137961	−3.583273	0.396801
4’b H	5.297070	−2.554852	1.830381
12’ O	5.784601	0.425785	−0.987006
7’ H	7.498603	−2.875249	0.272221
8’a H	7.232686	−1.783842	−2.248738
8’b H	8.809507	−2.258895	−1.568913
9’a H	9.013306	0.041580	−2.163406
9’b H	7.513337	0.365769	−1.447309
10’a H	10.028602	−0.387882	−0.096523
10’b H	9.024017	1.047589	0.199989
11’a H	8.734345	−1.403253	1.609269
11’b H	7.428332	−0.233134	1.451070

SCF GIAO magnetic shielding (ppm):

1 S	Isotropic = 426.7204 Anisotropy = 364.1228
2 C	Isotropic = 150.6873 Anisotropy = 19.0588
3 C	Isotropic = 41.4733 Anisotropy = 167.9625
4 C	Isotropic = 28.4926 Anisotropy = 139.0658
5 N	Isotropic = 61.8876 Anisotropy = 91.2121
6 C	Isotropic = 115.0929 Anisotropy = 35.9783
7 C	Isotropic = 119.8137 Anisotropy = 44.4538
8 C	Isotropic = 11.2156 Anisotropy = 84.3686
9 O	Isotropic = −36.0713 Anisotropy = 469.8293
10 C	Isotropic = 10.8141 Anisotropy = 81.0404

11 O	Isotropic =	−18.0790	Anisotropy =	363.5382
12 O	Isotropic =	−11.3177	Anisotropy =	360.1724
2a H	Isotropic =	28.7226	Anisotropy =	8.5446
2b H	Isotropic =	28.6191	Anisotropy =	14.0623
6 H	Isotropic =	26.8289	Anisotropy =	8.3075
7 H	Isotropic =	26.2934	Anisotropy =	4.8110
1” N	Isotropic =	118.0829	Anisotropy =	119.4613
2” C	Isotropic =	14.4683	Anisotropy =	111.2686
3” C	Isotropic =	24.6440	Anisotropy =	119.3079
4” C	Isotropic =	13.1066	Anisotropy =	84.3429
5” N	Isotropic =	−2.1095	Anisotropy =	261.4314
6” C	Isotropic =	−8.2980	Anisotropy =	125.3298
7” S	Isotropic =	77.1840	Anisotropy =	248.9955
8” N	Isotropic =	−35.0826	Anisotropy =	260.4871
1” H	Isotropic =	25.5251	Anisotropy =	9.7191
9” O	Isotropic =	−57.2847	Anisotropy =	573.2226
10” N	Isotropic =	−156.1352	Anisotropy =	366.3343
11” O	Isotropic =	96.1742	Anisotropy =	141.5876
11” H	Isotropic =	24.5880	Anisotropy =	8.8977
12” N	Isotropic =	168.1967	Anisotropy =	88.5296
12”a H	Isotropic =	26.5805	Anisotropy =	10.1033
12”b H	Isotropic =	26.1971	Anisotropy =	12.0931
1’ C	Isotropic =	47.5013	Anisotropy =	166.3861
2’ C	Isotropic =	45.2657	Anisotropy =	119.6853
3’ C	Isotropic =	154.9997	Anisotropy =	17.3835
4’ C	Isotropic =	131.2699	Anisotropy =	54.1743
5’ N	Isotropic =	90.8741	Anisotropy =	99.3808
6’ C	Isotropic =	4.2836	Anisotropy =	100.7927
7’ C	Isotropic =	123.8920	Anisotropy =	29.7831
8’ C	Isotropic =	127.1559	Anisotropy =	37.1746
9’ N	Isotropic =	182.5010	Anisotropy =	38.3806
10’ C	Isotropic =	131.5757	Anisotropy =	55.7877
11’ C	Isotropic =	149.9217	Anisotropy =	37.8310
1’ H	Isotropic =	23.2142	Anisotropy =	11.7457
3’a H	Isotropic =	28.8744	Anisotropy =	7.1208
3’b H	Isotropic =	28.9956	Anisotropy =	8.3973
4’a H	Isotropic =	28.3319	Anisotropy =	6.8254
4’b H	Isotropic =	28.2767	Anisotropy =	6.4324
12’ O	Isotropic =	26.9220	Anisotropy =	467.2386
7’ H	Isotropic =	27.7574	Anisotropy =	6.3438
8’a H	Isotropic =	28.2543	Anisotropy =	7.1080
8’b H	Isotropic =	28.5112	Anisotropy =	9.9347
9’a H	Isotropic =	27.1428	Anisotropy =	16.9805
9’b H	Isotropic =	21.8481	Anisotropy =	20.7159
10’a H	Isotropic =	28.7475	Anisotropy =	10.9313
10’b H	Isotropic =	28.0199	Anisotropy =	9.3416
11’a H	Isotropic =	29.1357	Anisotropy =	9.8160
11’b H	Isotropic =	29.7785	Anisotropy =	6.3140

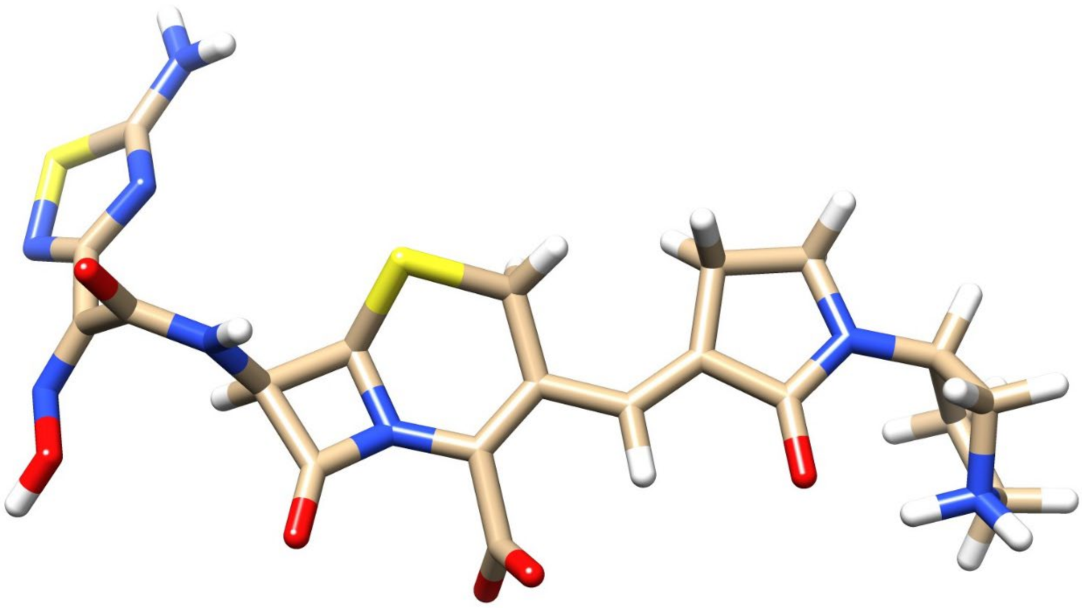

9

Energy = −2461.60950802 Hartree

Atomic coordinates (Å):

	X	Y	Z
1 S	0.954043	−0.843265	1.218261
2 C	−0.752660	−1.020452	0.535992
3 C	−1.441843	0.269400	0.119787
4 C	−0.756349	1.393565	−0.246532
5 N	0.632812	1.365483	−0.311074
6 C	1.500989	0.191104	−0.178824
7 C	2.704475	1.170597	0.020512
8 C	1.631304	2.293736	−0.060158
9 O	1.629121	3.490698	0.074751
10 C	−1.399461	2.731662	−0.669783
11 O	−1.989698	3.382230	0.219745
12 O	−1.251059	3.011507	−1.881280
2a H	−1.307280	−1.491517	1.345657
2b H	−0.705735	−1.733661	−0.290226
6 H	1.541088	−0.407086	−1.088972
7 H	3.367555	1.170748	−0.842887
1” N	3.475121	1.114154	1.229260
2” C	4.814079	0.927100	1.335102
3” C	5.619964	0.764335	0.051216
4” C	5.990628	−0.610501	−0.347940
5” N	5.289070	−1.638523	0.219339
6” C	5.732846	−2.782435	−0.258832
7” S	7.038287	−2.521432	−1.415310
8” N	6.945785	−0.848244	−1.215976

1” H	2.992742	1.224601	2.114271
9” O	5.399174	0.893924	2.408790
10” N	6.066987	1.754354	−0.625149
11” O	5.639080	2.973442	−0.115934
11” H	6.023910	3.624863	−0.716757
12” N	5.236422	−3.986099	0.067077
12”a H	4.560212	−4.043656	0.813970
12”b H	5.686982	−4.838779	−0.223143
1’ C	−2.890696	0.312875	0.088208
2’ C	−3.812919	−0.660877	0.257598
3’ C	−3.718063	−2.146532	0.530685
4’ C	−5.147666	−2.670722	0.277198
5’ N	−5.977450	−1.457870	0.262567
6’ C	−5.247942	−0.315462	0.158989
7’ C	−7.426120	−1.545536	0.080637
8’ C	−8.213300	−0.562870	0.965266
9’ N	−8.415706	0.622334	0.067671
10’ C	−8.764492	0.048076	−1.280273
11’ C	−7.896054	−1.219293	−1.376776
1’ H	−3.313543	1.292407	−0.112484
3’a H	−3.434147	−2.336500	1.569965
3’b H	−2.996915	−2.657290	−0.106541
4’a H	−5.491162	−3.350587	1.058347
4’b H	−5.224392	−3.185285	−0.685959
12’ O	−5.731548	0.823063	0.009169
7’ H	−7.701347	−2.561921	0.354201
8’a H	−7.695794	−0.259661	1.871624
8’b H	−9.192739	−0.968241	1.214355
9’a H	−9.111318	1.282551	0.414739
9’b H	−7.478678	1.075959	0.004131
10’a H	−9.829729	−0.176878	−1.273231
10’b H	−8.557052	0.791776	−2.046145
11’a H	−8.471202	−2.044177	−1.794085
11’b H	−7.030909	−1.057174	−2.018411

SCF GIAO magnetic shielding (ppm):

1 S	Isotropic = 493.7564 Anisotropy = 224.6895
2 C	Isotropic = 148.9844 Anisotropy = 20.5831
3 C	Isotropic = 66.6384 Anisotropy = 138.0519
4 C	Isotropic = 30.5671 Anisotropy = 150.8560
5 N	Isotropic = 66.3954 Anisotropy = 115.5443
6 C	Isotropic = 115.9593 Anisotropy = 30.4637
7 C	Isotropic = 116.2471 Anisotropy = 31.0540
8 C	Isotropic = 13.5549 Anisotropy = 75.7883
9 O	Isotropic = −58.6869 Anisotropy = 517.5637
10 C	Isotropic = 9.9493 Anisotropy = 85.2763
11 O	Isotropic = −27.8888 Anisotropy = 367.7165

12 O	Isotropic =	−23.4466	Anisotropy =	367.2155
2a H	Isotropic =	28.2583	Anisotropy =	13.1601
2b H	Isotropic =	27.9924	Anisotropy =	9.2389
6 H	Isotropic =	26.9201	Anisotropy =	6.6693
7 H	Isotropic =	27.0177	Anisotropy =	6.4779
1” N	Isotropic =	124.1165	Anisotropy =	117.8596
2” C	Isotropic =	11.1271	Anisotropy =	106.9179
3” C	Isotropic =	27.2368	Anisotropy =	116.2329
4” C	Isotropic =	15.7315	Anisotropy =	84.8184
5” N	Isotropic =	−13.2793	Anisotropy =	257.0544
6” C	Isotropic =	−9.2488	Anisotropy =	129.5996
7” S	Isotropic =	77.1388	Anisotropy =	253.1377
8” N	Isotropic =	−21.1403	Anisotropy =	242.8557
1” H	Isotropic =	26.0026	Anisotropy =	5.1388
9” O	Isotropic =	−64.8239	Anisotropy =	536.5289
10” N	Isotropic =	−152.8445	Anisotropy =	369.3482
11” O	Isotropic =	93.3743	Anisotropy =	152.5790
11” H	Isotropic =	24.3052	Anisotropy =	9.2326
12” N	Isotropic =	168.1482	Anisotropy =	85.6526
12”a H	Isotropic =	26.2097	Anisotropy =	10.7824
12”b H	Isotropic =	26.5646	Anisotropy =	10.2966
1’ C	Isotropic =	44.2041	Anisotropy =	160.9195
2’ C	Isotropic =	50.8185	Anisotropy =	113.1600
3’ C	Isotropic =	154.1731	Anisotropy =	19.3304
4’ C	Isotropic =	131.4101	Anisotropy =	53.2416
5’ N	Isotropic =	94.5550	Anisotropy =	99.4070
6’ C	Isotropic =	4.0766	Anisotropy =	103.7988
7’ C	Isotropic =	123.7343	Anisotropy =	29.8085
8’ C	Isotropic =	127.1424	Anisotropy =	40.4702
9’ N	Isotropic =	182.2054	Anisotropy =	38.8777
10’ C	Isotropic =	132.2944	Anisotropy =	55.4793
11’ C	Isotropic =	149.7004	Anisotropy =	37.8756
1’ H	Isotropic =	24.1889	Anisotropy =	11.7133
3’a H	Isotropic =	28.7755	Anisotropy =	7.1679
3’b H	Isotropic =	28.9360	Anisotropy =	7.9099
4’a H	Isotropic =	28.4025	Anisotropy =	6.8113
4’b H	Isotropic =	28.2968	Anisotropy =	6.5792
12’ O	Isotropic =	36.8331	Anisotropy =	463.0822
7’ H	Isotropic =	27.9324	Anisotropy =	6.2933
8’a H	Isotropic =	28.2934	Anisotropy =	6.9991
8’b H	Isotropic =	28.5262	Anisotropy =	10.0317
9’a H	Isotropic =	27.2144	Anisotropy =	17.0919
9’b H	Isotropic =	21.6861	Anisotropy =	21.4140
10’a H	Isotropic =	28.6842	Anisotropy =	11.0449
10’b H	Isotropic =	28.1139	Anisotropy =	9.2612
11’a H	Isotropic =	29.2621	Anisotropy =	9.7042
11’b H	Isotropic =	29.8940	Anisotropy =	6.3373

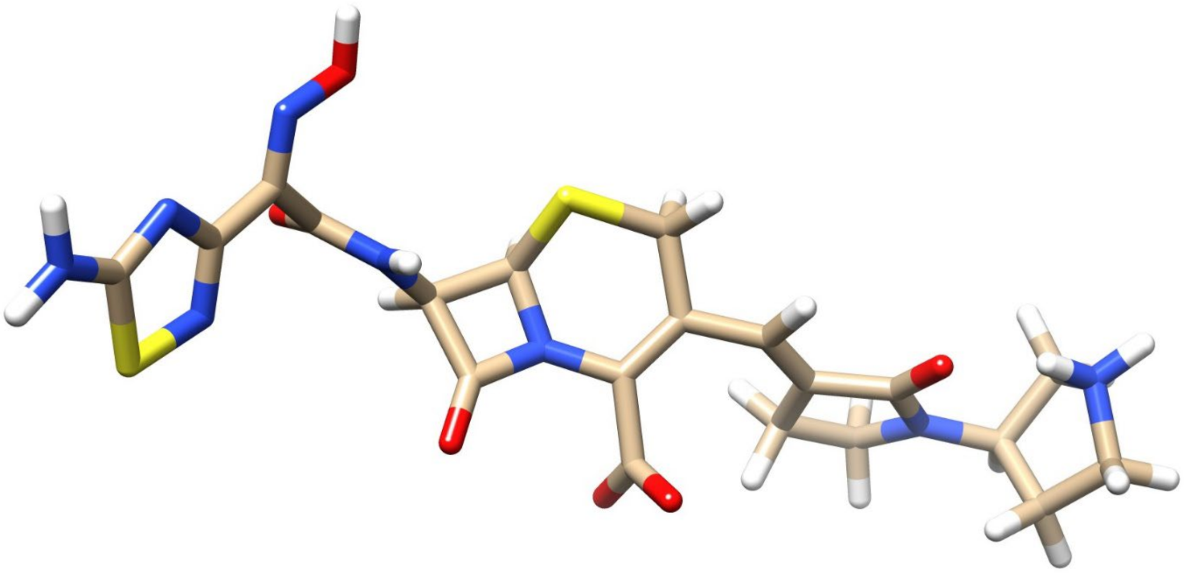

10

Energy = −2461.60902963 Hartree

Atomic coordinates (Å):

	X	Y	Z
1 S	−0.695772	2.112162	−0.085710
2 C	1.142811	1.985856	−0.148467
3 C	1.733405	0.582022	−0.126823
4 C	1.056465	−0.529993	−0.504927
5 N	−0.244404	−0.395261	−1.010399
6 C	−0.920854	0.849344	−1.385432
7 C	−2.262378	0.047386	−1.403925
8 C	−1.407095	−1.133507	−0.876628
9 O	−1.643054	−2.243227	−0.466390
10 C	1.563494	−1.984095	−0.427810
11 O	1.911420	−2.405052	0.697618
12 O	1.559380	−2.596450	−1.522011
2a H	1.491761	2.537403	0.726922
2b H	1.490659	2.533412	−1.028657
6 H	−0.592058	1.241164	−2.347776
7 H	−2.627679	−0.107916	−2.418159
1” N	−3.364292	0.458251	−0.580944
2” C	−4.566279	0.820250	−1.096145
3” C	−5.622665	1.183271	−0.059724
4” C	−6.526945	0.117182	0.404953
5” N	−7.550018	0.378243	1.271964
6” C	−8.191779	−0.740100	1.528437
7” S	−7.482225	−2.106273	0.657926
8” N	−6.317141	−1.104199	−0.035903

1” H	−3.256650	0.446845	0.424330
9” O	−4.824539	0.872876	−2.289134
10” N	−5.793188	2.376330	0.373252
11” O	−4.864352	3.265243	−0.154442
11” H	−5.097629	4.110627	0.250687
12” N	−9.236996	−0.844911	2.362866
12”a H	−9.635725	−0.003530	2.751650
12”b H	−9.761634	−1.699824	2.451152
1’ C	3.114425	0.576833	0.365385
2’ C	4.208645	0.032431	−0.190228
3’ C	4.396678	−0.738248	−1.471033
4’ C	5.920360	−0.703859	−1.715127
5’ N	6.478557	−0.250387	−0.430732
6’ C	5.542564	0.213449	0.432836
7’ C	7.919802	−0.250180	−0.178968
8’ C	8.477113	1.126301	0.230229
9’ N	8.464631	1.070538	1.730521
10’ C	8.955151	−0.310263	2.079207
11’ C	8.337320	−1.203111	0.989116
1’ H	3.289540	1.166214	1.264779
3’a H	3.835557	−0.306440	−2.301378
3’b H	4.055983	−1.769099	−1.356756
4’a H	6.196678	0.000609	−2.505384
4’b H	6.324523	−1.684201	−1.972266
12’ O	5.785064	0.717675	1.544871
7’ H	8.387820	−0.556756	−1.111381
8’a H	7.889029	1.972208	−0.116452
8’b H	9.511351	1.237135	−0.091925
9’a H	9.009183	1.813094	2.168828
9’b H	7.462201	1.143163	2.002768
10’a H	10.042901	−0.289979	2.034983
10’b H	8.637894	−0.555352	3.090077
11’a H	9.058374	−1.944059	0.647753
11’b H	7.463052	−1.733518	1.362716

SCF GIAO magnetic shielding (ppm):

1 S	Isotropic = 499.9335 Anisotropy = 196.7383
2 C	Isotropic = 147.9739 Anisotropy = 29.5727
3 C	Isotropic = 65.1475 Anisotropy = 130.6013
4 C	Isotropic = 37.3775 Anisotropy = 142.9564
5 N	Isotropic = 68.5739 Anisotropy = 122.5585
6 C	Isotropic = 115.7674 Anisotropy = 30.9026
7 C	Isotropic = 122.2734 Anisotropy = 45.4305
8 C	Isotropic = 12.7348 Anisotropy = 77.1690
9 O	Isotropic = −50.6120 Anisotropy = 508.1090
10 C	Isotropic = 8.7064 Anisotropy = 91.1047
11 O	Isotropic = −15.3300 Anisotropy = 351.6340

12 O	Isotropic =	−7.7861	Anisotropy =	354.6000
2a H	Isotropic =	28.7311	Anisotropy =	9.2921
2b H	Isotropic =	28.3950	Anisotropy =	8.8594
6 H	Isotropic =	26.7300	Anisotropy =	7.3202
7 H	Isotropic =	26.0229	Anisotropy =	4.5973
1" N	Isotropic =	124.1106	Anisotropy =	111.3303
2" C	Isotropic =	13.5352	Anisotropy =	113.8356
3" C	Isotropic =	25.1286	Anisotropy =	116.5544
4" C	Isotropic =	12.9842	Anisotropy =	84.0186
5" N	Isotropic =	−2.1898	Anisotropy =	259.8074
6" C	Isotropic =	−8.1871	Anisotropy =	124.6745
7" S	Isotropic =	72.8160	Anisotropy =	248.5685
8" N	Isotropic =	−37.9402	Anisotropy =	253.9895
1" H	Isotropic =	25.9499	Anisotropy =	7.9373
9" O	Isotropic =	−61.9722	Anisotropy =	591.5570
10" N	Isotropic =	−156.9527	Anisotropy =	369.8294
11" O	Isotropic =	94.4631	Anisotropy =	146.0419
11" H	Isotropic =	24.5130	Anisotropy =	8.8002
12" N	Isotropic =	167.7759	Anisotropy =	88.3105
12"a H	Isotropic =	26.2160	Anisotropy =	12.1263
12"b H	Isotropic =	26.5424	Anisotropy =	10.0679
1' C	Isotropic =	43.4853	Anisotropy =	146.7447
2' C	Isotropic =	41.7417	Anisotropy =	123.0546
3' C	Isotropic =	154.5724	Anisotropy =	18.3726
4' C	Isotropic =	130.8291	Anisotropy =	54.2959
5' N	Isotropic =	89.6355	Anisotropy =	106.7060
6' C	Isotropic =	4.9730	Anisotropy =	102.3300
7' C	Isotropic =	124.1232	Anisotropy =	30.0663
8' C	Isotropic =	126.5613	Anisotropy =	39.3124
9' N	Isotropic =	182.6131	Anisotropy =	38.9278
10' C	Isotropic =	131.4715	Anisotropy =	55.1056
11' C	Isotropic =	149.2111	Anisotropy =	39.6334
1' H	Isotropic =	24.9928	Anisotropy =	8.5508
3'a H	Isotropic =	29.4295	Anisotropy =	6.4133
3'b H	Isotropic =	28.9055	Anisotropy =	10.3372
4'a H	Isotropic =	28.3385	Anisotropy =	7.1684
4'b H	Isotropic =	28.4020	Anisotropy =	6.1862
12' O	Isotropic =	32.3618	Anisotropy =	467.6531
7' H	Isotropic =	27.9898	Anisotropy =	5.9392
8'a H	Isotropic =	28.3523	Anisotropy =	6.8318
8'b H	Isotropic =	28.5362	Anisotropy =	10.0211
9'a H	Isotropic =	27.2473	Anisotropy =	17.1282
9'b H	Isotropic =	21.9308	Anisotropy =	20.6474
10'a H	Isotropic =	28.6752	Anisotropy =	11.1003
10'b H	Isotropic =	28.0377	Anisotropy =	9.2685
11'a H	Isotropic =	29.1605	Anisotropy =	9.5529
11'b H	Isotropic =	29.7015	Anisotropy =	6.5476

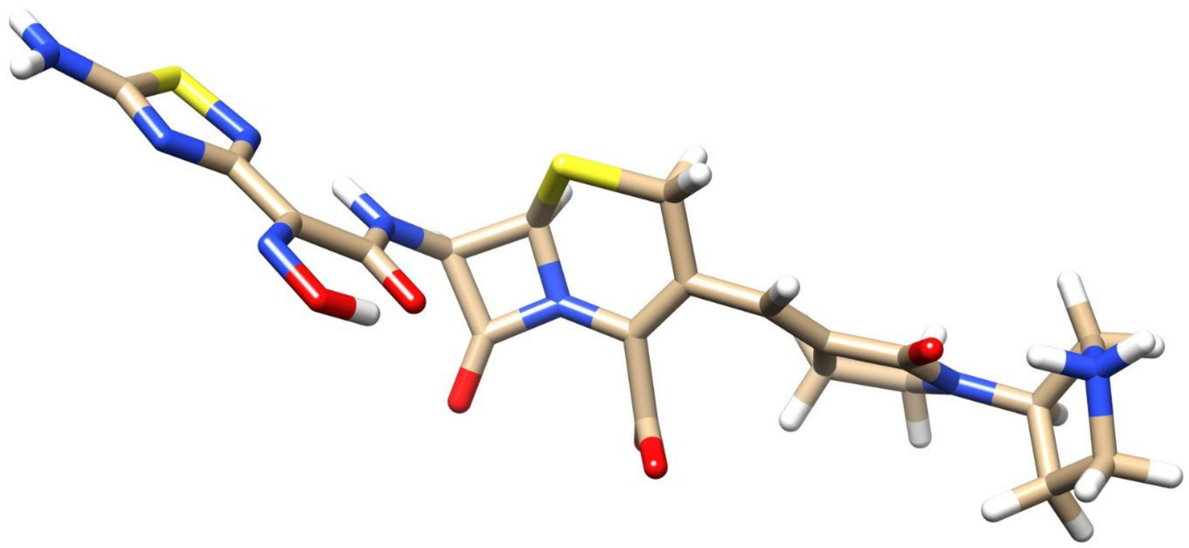

11

Energy = −2461.60912935 Hartree

Atomic coordinates (Å):

	X	Y	Z
1 S	1.068729	−0.879527	−1.521291
2 C	−0.771235	−0.828793	−1.651494
3 C	−1.508727	−0.062342	−0.561109
4 C	−0.953088	0.929320	0.179206
5 N	0.358740	1.327428	−0.094066
6 C	1.176893	0.918856	−1.235714
7 C	2.412646	1.535694	−0.500340
8 C	1.411062	1.830835	0.653170
9 O	1.482116	2.348919	1.739061
10 C	−1.624128	1.677547	1.348427
11 O	−1.924886	0.998606	2.355391
12 O	−1.785443	2.906455	1.160744
2a H	−1.082532	−1.875357	−1.631492
2b H	−1.032873	−0.437798	−2.638681
6 H	0.899421	1.442782	−2.151057
7 H	2.730025	2.454226	−0.994188
1” N	3.594778	0.760253	−0.229389
2” C	3.704670	−0.161954	0.742050
3” C	5.040752	−0.834741	0.946632
4” C	6.249999	−0.523620	0.137094
5” N	7.428149	−1.167714	0.385658
6” C	8.350416	−0.732109	−0.443321
7” S	7.715000	0.498948	−1.539722
8” N	6.193989	0.377277	−0.821469

1” H	4.445793	0.957287	−0.762377
9” O	2.750091	−0.476237	1.476620
10” N	5.227962	−1.743417	1.855915
11” O	4.209350	−2.111922	2.660984
11” H	3.406848	−1.569420	2.400498
12” N	9.614568	−1.176786	−0.473141
12”a H	9.917571	−1.833226	0.230544
12”b H	10.313368	−0.762125	−1.067797
1’ C	−2.884366	−0.537921	−0.380709
2’ C	−4.027971	0.165400	−0.396198
3’ C	−4.290361	1.632810	−0.622363
4’ C	−5.808774	1.712089	−0.891574
5’ N	−6.315715	0.388711	−0.494032
6’ C	−5.336951	−0.516674	−0.251083
7’ C	−7.749992	0.108009	−0.423801
8’ C	−8.186869	−1.107204	−1.263256
9’ N	−8.163490	−2.235455	−0.273606
10’ C	−8.760317	−1.670073	0.988927
11’ C	−8.238010	−0.223275	1.025004
1’ H	−2.998397	−1.618380	−0.296562
3’a H	−3.705021	2.029835	−1.453727
3’b H	−4.026669	2.219776	0.258818
4’a H	−6.033359	1.889228	−1.947561
4’b H	−6.296439	2.490282	−0.302161
12’ O	−5.526836	−1.714584	0.030395
7’ H	−8.249119	0.996150	−0.803984
8’a H	−7.534118	−1.333978	−2.102423
8’b H	−9.211569	−0.988333	−1.612100
9’a H	−8.641914	−3.073853	−0.602861
9’b H	−7.154794	−2.431199	−0.102695
10’a H	−9.843150	−1.721123	0.888267
10’b H	−8.446219	−2.279056	1.833396
11’a H	−9.027200	0.459873	1.334619
11’b H	−7.411210	−0.119444	1.725801

SCF GIAO magnetic shielding (ppm):

1 S	Isotropic = 496.5713 Anisotropy = 202.6621
2 C	Isotropic = 147.6416 Anisotropy = 28.5446
3 C	Isotropic = 67.5459 Anisotropy = 126.8493
4 C	Isotropic = 37.5082 Anisotropy = 145.4139
5 N	Isotropic = 70.6228 Anisotropy = 127.0908
6 C	Isotropic = 115.6523 Anisotropy = 31.0604
7 C	Isotropic = 119.0778 Anisotropy = 38.0130
8 C	Isotropic = 16.2143 Anisotropy = 76.4645
9 O	Isotropic = −48.5042 Anisotropy = 500.9940
10 C	Isotropic = 8.0906 Anisotropy = 91.6279
11 O	Isotropic = −15.6350 Anisotropy = 348.4701

12 O	Isotropic = −9.4396	Anisotropy = 357.7424
2a H	Isotropic = 28.8868	Anisotropy = 9.2620
2b H	Isotropic = 28.4342	Anisotropy = 9.1301
6 H	Isotropic = 26.8160	Anisotropy = 7.9636
7 H	Isotropic = 27.1090	Anisotropy = 5.1053
1” N	Isotropic = 119.8407	Anisotropy = 64.1656
2” C	Isotropic = 12.0856	Anisotropy = 108.1918
3” C	Isotropic = 37.1277	Anisotropy = 135.4950
4” C	Isotropic = 11.1587	Anisotropy = 82.6154
5” N	Isotropic = −2.8937	Anisotropy = 256.1687
6” C	Isotropic = −6.2906	Anisotropy = 125.2210
7” S	Isotropic = 84.1665	Anisotropy = 275.1063
8” N	Isotropic = −10.5779	Anisotropy = 223.7055
1” H	Isotropic = 21.2738	Anisotropy = 21.4091
9” O	Isotropic = −12.2205	Anisotropy = 469.3505
10” N	Isotropic = −230.6102	Anisotropy = 475.7429
11” O	Isotropic = 51.8509	Anisotropy = 336.0973
11” H	Isotropic = 15.3386	Anisotropy = 21.3388
12” N	Isotropic = 167.4448	Anisotropy = 87.7002
12”a H	Isotropic = 26.1385	Anisotropy = 12.2507
12”b H	Isotropic = 26.4783	Anisotropy = 10.1918
1’ C	Isotropic = 42.5647	Anisotropy = 148.5603
2’ C	Isotropic = 42.0886	Anisotropy = 122.3365
3’ C	Isotropic = 154.2985	Anisotropy = 18.9091
4’ C	Isotropic = 131.1883	Anisotropy = 54.8967
5’ N	Isotropic = 90.3562	Anisotropy = 106.2987
6’ C	Isotropic = 5.3298	Anisotropy = 103.1587
7’ C	Isotropic = 124.7224	Anisotropy = 27.9866
8’ C	Isotropic = 126.6343	Anisotropy = 38.7083
9’ N	Isotropic = 181.8484	Anisotropy = 38.5766
10’ C	Isotropic = 131.5792	Anisotropy = 55.6199
11’ C	Isotropic = 149.1176	Anisotropy = 39.9785
1’ H	Isotropic = 24.9213	Anisotropy = 8.6001
3’a H	Isotropic = 29.3844	Anisotropy = 6.2213
3’b H	Isotropic = 28.6787	Anisotropy = 10.8920
4’a H	Isotropic = 28.3059	Anisotropy = 7.1954
4’b H	Isotropic = 28.3913	Anisotropy = 6.0917
12’ O	Isotropic = 33.6377	Anisotropy = 463.5252
7’ H	Isotropic = 27.9726	Anisotropy = 6.1087
8’a H	Isotropic = 28.3823	Anisotropy = 6.8519
8’b H	Isotropic = 28.5305	Anisotropy = 10.0343
9’a H	Isotropic = 27.1037	Anisotropy = 17.0804
9’b H	Isotropic = 21.8257	Anisotropy = 20.9283
10’a H	Isotropic = 28.6474	Anisotropy = 11.0824
10’b H	Isotropic = 28.0366	Anisotropy = 9.2038
11’a H	Isotropic = 29.1690	Anisotropy = 9.6616
11’b H	Isotropic = 29.7037	Anisotropy = 6.3516

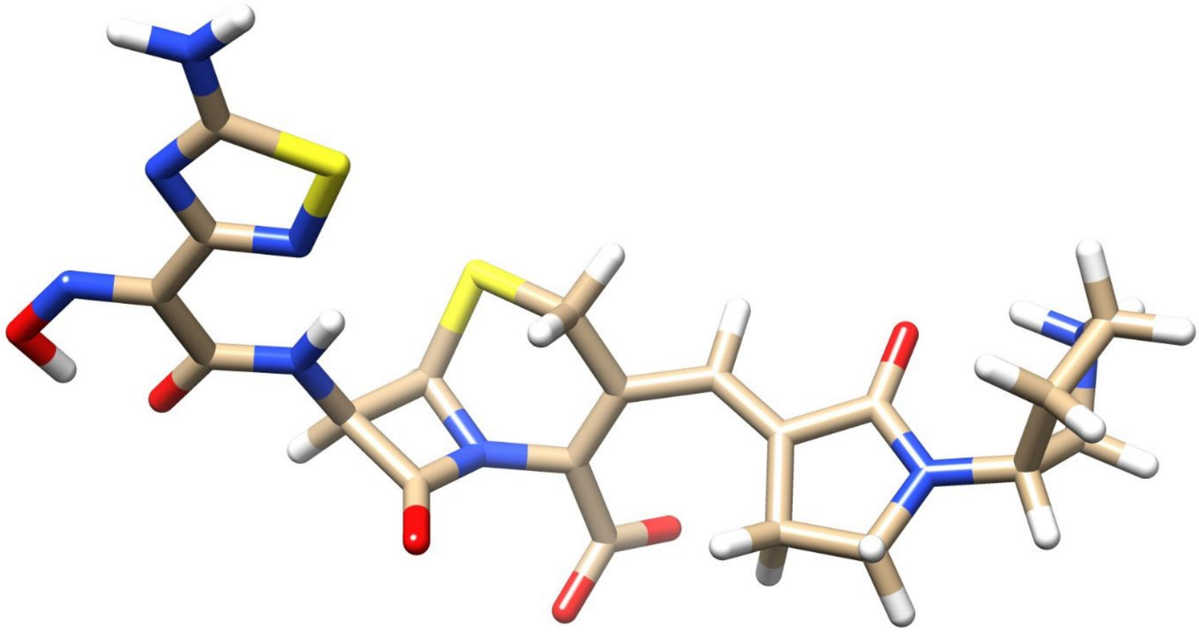

12

Energy = −2461.60745619 Hartree

Atomic coordinates (Å):

	X	Y	Z
1 S	1.119436	−0.351950	2.089788
2 C	−0.196771	0.267564	0.933305
3 C	−1.282155	−0.750355	0.633546
4 C	−0.908848	−1.979362	0.202671
5 N	0.504145	−2.171886	0.134652
6 C	1.451812	−1.947928	1.240341
7 C	2.603101	−2.064266	0.179404
8 C	1.449717	−2.104530	−0.861908
9 O	1.384896	−2.064049	−2.068007
10 C	−1.768306	−3.215980	−0.085990
11 O	−2.790839	−3.377868	0.619825
12 O	−1.338591	−3.960487	−1.000911
2a H	0.279376	0.605730	0.011226
2b H	−0.606087	1.145470	1.431593
6 H	1.449438	−2.734120	1.994440
7 H	3.119321	−3.020471	0.249838
1” N	3.575360	−1.020341	0.058966
2” C	4.895865	−1.266498	0.018122
3” C	5.845667	−0.102334	−0.119207
4” C	5.399115	1.314307	−0.208569
5” N	6.316366	2.326223	−0.199992
6” C	5.695613	3.481397	−0.289830
7” S	3.943504	3.276396	−0.382841
8” N	4.117558	1.600905	−0.299626

1” H	3.290281	−0.040920	−0.022656
9” O	5.364503	−2.420558	0.094204
10” N	7.134514	−0.258115	−0.167307
11” O	7.667421	−1.495856	−0.094209
11” H	6.907188	−2.145321	−0.005176
12” N	6.307422	4.673522	−0.295198
12”a H	5.801056	5.534729	−0.419100
12”b H	7.315577	4.707891	−0.288424
1’ C	−2.643724	−0.252685	0.833100
2’ C	−3.697444	−0.356134	0.009310
3’ C	−3.861568	−1.053083	−1.310286
4’ C	−5.110266	−0.396133	−1.928906
5’ N	−5.769463	0.253814	−0.782817
6’ C	−4.967389	0.355189	0.306932
7’ C	−7.049730	0.944981	−0.945996
8’ C	−8.028051	0.704535	0.216691
9’ N	−7.786364	1.877486	1.120645
10’ C	−7.649602	3.065608	0.204344
11’ C	−6.911997	2.501881	−1.023417
1’ H	−2.785199	0.379050	1.708248
3’a H	−4.036338	−2.118109	−1.135228
3’b H	−2.981521	−0.966080	−1.949695
4’a H	−5.786635	−1.119567	−2.385951
4’b H	−4.848103	0.355545	−2.680112
12’ O	−5.255515	0.952341	1.358667
7’ H	−7.486923	0.550949	−1.860512
8’a H	−7.867000	−0.223991	0.758237
8’b H	−9.058168	0.757593	−0.132445
9’a H	−8.510333	1.998274	1.828588
9’b H	−6.865109	1.693830	1.570065
10’a H	−8.654625	3.407707	−0.035826
10’b H	−7.112742	3.855324	0.724935
11’a H	−7.352709	2.885757	−1.941937
11’b H	−5.859781	2.783101	−1.015021

SCF GIAO magnetic shielding (ppm):

1 S	Isotropic = 426.5599 Anisotropy = 361.6480
2 C	Isotropic = 145.8384 Anisotropy = 26.6598
3 C	Isotropic = 37.3779 Anisotropy = 161.5859
4 C	Isotropic = 32.4064 Anisotropy = 111.0520
5 N	Isotropic = 72.5511 Anisotropy = 100.1843
6 C	Isotropic = 112.2080 Anisotropy = 35.2746
7 C	Isotropic = 120.5608 Anisotropy = 47.9016
8 C	Isotropic = 8.2962 Anisotropy = 85.0756
9 O	Isotropic = −30.3004 Anisotropy = 462.4364
10 C	Isotropic = 9.3928 Anisotropy = 86.5067
11 O	Isotropic = −11.4436 Anisotropy = 350.1824

12 O	Isotropic =	−8.9040	Anisotropy =	366.1185
2a H	Isotropic =	28.4790	Anisotropy =	7.6100
2b H	Isotropic =	29.0104	Anisotropy =	9.4105
6 H	Isotropic =	26.8377	Anisotropy =	8.4807
7 H	Isotropic =	26.0492	Anisotropy =	6.2460
1” N	Isotropic =	113.8457	Anisotropy =	81.4795
2” C	Isotropic =	13.4501	Anisotropy =	107.2457
3” C	Isotropic =	36.9878	Anisotropy =	137.3230
4” C	Isotropic =	11.5278	Anisotropy =	83.1485
5” N	Isotropic =	−3.2199	Anisotropy =	254.3256
6” C	Isotropic =	−6.1390	Anisotropy =	125.9253
7” S	Isotropic =	83.8512	Anisotropy =	281.0305
8” N	Isotropic =	−11.3798	Anisotropy =	223.2297
1” H	Isotropic =	21.1201	Anisotropy =	16.3075
9” O	Isotropic =	15.9383	Anisotropy =	453.6214
10” N	Isotropic =	−231.2776	Anisotropy =	471.6292
11” O	Isotropic =	51.2379	Anisotropy =	333.4403
11” H	Isotropic =	15.1904	Anisotropy =	20.3716
12” N	Isotropic =	167.4509	Anisotropy =	88.2642
12”a H	Isotropic =	26.4474	Anisotropy =	9.9534
12”b H	Isotropic =	26.0956	Anisotropy =	12.6119
1’ C	Isotropic =	48.3571	Anisotropy =	141.5897
2’ C	Isotropic =	35.3943	Anisotropy =	132.9074
3’ C	Isotropic =	153.7415	Anisotropy =	22.5234
4’ C	Isotropic =	130.6108	Anisotropy =	52.7347
5’ N	Isotropic =	87.8786	Anisotropy =	98.3288
6’ C	Isotropic =	4.5642	Anisotropy =	102.5663
7’ C	Isotropic =	123.2854	Anisotropy =	30.1563
8’ C	Isotropic =	127.5705	Anisotropy =	39.3374
9’ N	Isotropic =	182.5673	Anisotropy =	38.4388
10’ C	Isotropic =	131.9332	Anisotropy =	55.6868
11’ C	Isotropic =	149.1490	Anisotropy =	39.3867
1’ H	Isotropic =	24.7694	Anisotropy =	9.2047
3’a H	Isotropic =	28.9315	Anisotropy =	9.3472
3’b H	Isotropic =	29.4948	Anisotropy =	6.5501
4’a H	Isotropic =	28.3628	Anisotropy =	6.3917
4’b H	Isotropic =	28.2323	Anisotropy =	6.6192
12’ O	Isotropic =	28.9576	Anisotropy =	473.0692
7’ H	Isotropic =	27.8547	Anisotropy =	6.0973
8’a H	Isotropic =	28.2304	Anisotropy =	7.2457
8’b H	Isotropic =	28.4979	Anisotropy =	10.0156
9’a H	Isotropic =	27.1045	Anisotropy =	17.0897
9’b H	Isotropic =	21.9114	Anisotropy =	20.5968
10’a H	Isotropic =	28.6199	Anisotropy =	11.0739
10’b H	Isotropic =	28.0275	Anisotropy =	9.2209
11’a H	Isotropic =	29.1978	Anisotropy =	9.6485
11’b H	Isotropic =	29.8039	Anisotropy =	6.6207

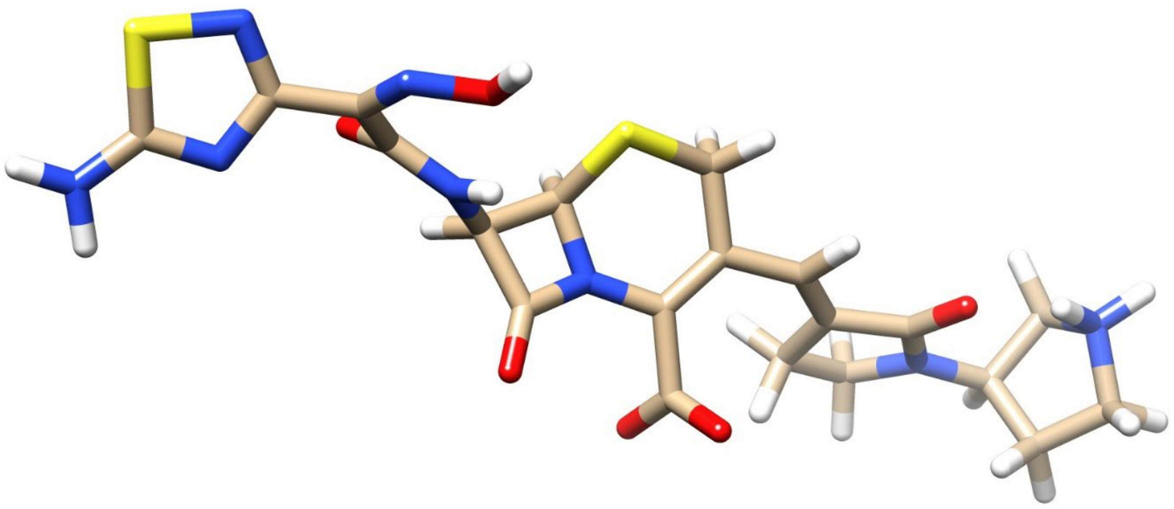

13

Energy = −2461.60643012 Hartree

Atomic coordinates (Å):

	X	Y	Z
1 S	0.655976	−1.765700	−0.539836
2 C	−1.169010	−1.647384	−0.772460
3 C	−1.854816	−0.428531	−0.168753
4 C	−1.219890	0.736007	0.112291
5 N	0.132271	0.873416	−0.230395
6 C	0.927206	−0.036166	−1.059016
7 C	2.206239	0.697374	−0.534353
8 C	1.221452	1.468940	0.380804
9 O	1.335934	2.248898	1.294075
10 C	−1.833587	1.968039	0.807229
11 O	−2.347587	1.785834	1.933056
12 O	−1.738651	3.036381	0.157714
2a H	−1.569896	−2.551944	−0.310222
2b H	−1.380899	−1.716018	−1.842934
6 H	0.726594	0.070465	−2.124822
7 H	2.649990	1.324035	−1.306597
1” N	3.250373	−0.030652	0.127128
2” C	4.468633	−0.212780	−0.443097
3” C	5.517899	−0.946648	0.377684
4” C	6.929386	−0.534986	0.139475
5” N	7.251861	0.788199	0.242150
6” C	8.542927	0.934589	0.031333
7” S	9.315691	−0.615434	−0.302216
8” N	7.841856	−1.433448	−0.138094

1” H	3.051654	−0.513786	0.993451
9” O	4.769690	0.196708	−1.556873
10” N	5.360454	−1.911798	1.204160
11” O	4.039261	−2.291111	1.395048
11” H	4.093111	−2.995990	2.053793
12” N	9.188082	2.112829	0.038902
12”a H	8.688958	2.934121	0.346751
12”b H	10.193708	2.160921	0.010451
1’ C	−3.277627	−0.669334	0.088016
2’ C	−4.337942	0.067195	−0.280092
3’ C	−4.436098	1.352475	−1.059601
4’ C	−5.915437	1.419087	−1.494320
5’ N	−6.581592	0.414515	−0.648841
6’ C	−5.717235	−0.393203	0.012600
7’ C	−8.039405	0.295411	−0.605198
8’ C	−8.558880	−1.115362	−0.939058
9’ N	−8.700249	−1.757255	0.410151
10’ C	−9.303319	−0.695617	1.292507
11’ C	−8.638047	0.604573	0.806531
1’ H	−3.509585	−1.616156	0.574282
3’a H	−3.753075	1.377645	−1.910165
3’b H	−4.191445	2.207962	−0.426542
4’a H	−6.048237	1.158087	−2.548619
4’b H	−6.357296	2.402056	−1.324625
12’ O	−6.045354	−1.359916	0.725227
7’ H	−8.420342	0.994992	−1.345365
8’a H	−7.894336	−1.705034	−1.565429
8’b H	−9.548375	−1.064594	−1.391015
9’a H	−9.240362	−2.622105	0.393501
9’b H	−7.726199	−1.939640	0.732836
10’a H	−10.378422	−0.702834	1.121298
10’b H	−9.099805	−0.940400	2.332454
11’a H	−9.369118	1.409300	0.749599
11’b H	−7.845735	0.919996	1.483514

SCF GIAO magnetic shielding (ppm):

1 S	Isotropic = 494.4746 Anisotropy = 205.2087
2 C	Isotropic = 147.4800 Anisotropy = 29.9797
3 C	Isotropic = 64.4147 Anisotropy = 131.9055
4 C	Isotropic = 37.2066 Anisotropy = 143.4844
5 N	Isotropic = 68.1218 Anisotropy = 119.6940
6 C	Isotropic = 115.6935 Anisotropy = 30.8002
7 C	Isotropic = 121.2072 Anisotropy = 44.5708
8 C	Isotropic = 12.3651 Anisotropy = 78.6602
9 O	Isotropic = −50.7481 Anisotropy = 508.1459
10 C	Isotropic = 8.7344 Anisotropy = 90.7341
11 O	Isotropic = −15.6539 Anisotropy = 352.2119

12 O	Isotropic =	−6.0230	Anisotropy =	354.6236
2a H	Isotropic =	28.6892	Anisotropy =	9.1339
2b H	Isotropic =	28.3767	Anisotropy =	8.8938
6 H	Isotropic =	26.6903	Anisotropy =	7.4129
7 H	Isotropic =	26.0084	Anisotropy =	5.1083
1” N	Isotropic =	120.6758	Anisotropy =	110.6914
2” C	Isotropic =	17.9581	Anisotropy =	101.7009
3” C	Isotropic =	27.6967	Anisotropy =	110.0946
4” C	Isotropic =	11.9927	Anisotropy =	88.7988
5” N	Isotropic =	−12.3969	Anisotropy =	258.2915
6” C	Isotropic =	−8.9347	Anisotropy =	127.5079
7” S	Isotropic =	82.4599	Anisotropy =	252.8764
8” N	Isotropic =	−31.2206	Anisotropy =	231.8020
1” H	Isotropic =	24.2418	Anisotropy =	8.2953
9” O	Isotropic =	−63.9974	Anisotropy =	584.8418
10” N	Isotropic =	−156.2961	Anisotropy =	364.2572
11” O	Isotropic =	88.4829	Anisotropy =	154.8666
11” H	Isotropic =	23.9911	Anisotropy =	7.3517
12” N	Isotropic =	168.6362	Anisotropy =	88.0599
12”a H	Isotropic =	26.2414	Anisotropy =	11.5757
12”b H	Isotropic =	26.5679	Anisotropy =	10.3677
1’ C	Isotropic =	44.9061	Anisotropy =	144.5183
2’ C	Isotropic =	41.3344	Anisotropy =	123.8213
3’ C	Isotropic =	153.9466	Anisotropy =	18.7797
4’ C	Isotropic =	130.4951	Anisotropy =	54.3117
5’ N	Isotropic =	89.2505	Anisotropy =	108.5366
6’ C	Isotropic =	4.6963	Anisotropy =	100.3694
7’ C	Isotropic =	124.6772	Anisotropy =	27.8768
8’ C	Isotropic =	126.2622	Anisotropy =	37.1702
9’ N	Isotropic =	182.4033	Anisotropy =	39.0752
10’ C	Isotropic =	131.6850	Anisotropy =	55.0561
11’ C	Isotropic =	149.4751	Anisotropy =	39.0395
1’ H	Isotropic =	25.0288	Anisotropy =	8.4756
3’a H	Isotropic =	29.3690	Anisotropy =	5.9697
3’b H	Isotropic =	28.8117	Anisotropy =	10.1590
4’a H	Isotropic =	28.2521	Anisotropy =	7.0943
4’b H	Isotropic =	28.2615	Anisotropy =	5.9143
12’ O	Isotropic =	32.7564	Anisotropy =	459.6851
7’ H	Isotropic =	27.8642	Anisotropy =	6.2343
8’a H	Isotropic =	28.2723	Anisotropy =	6.7677
8’b H	Isotropic =	28.4879	Anisotropy =	10.0517
9’a H	Isotropic =	27.1488	Anisotropy =	16.9545
9’b H	Isotropic =	21.6142	Anisotropy =	21.2633
10’a H	Isotropic =	28.7026	Anisotropy =	11.0614
10’b H	Isotropic =	27.9870	Anisotropy =	9.2683
11’a H	Isotropic =	29.0893	Anisotropy =	9.6597
11’b H	Isotropic =	29.6949	Anisotropy =	6.4367

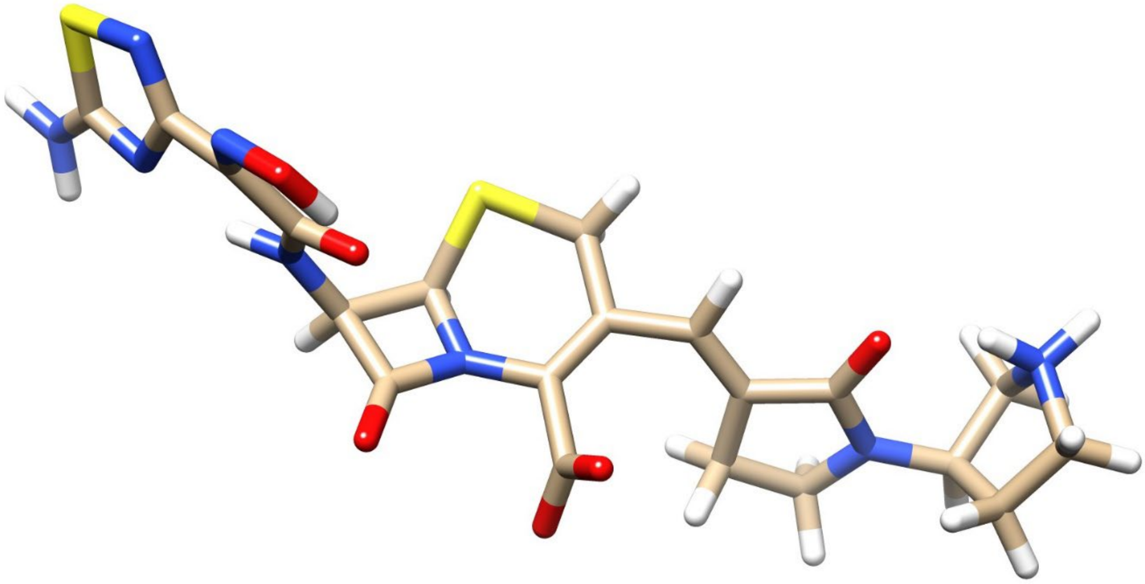

14

Energy = −2461.60785549 Hartree

Atomic coordinates (Å):

	X	Y	Z
1 S	1.156987	−1.209637	−1.069298
2 C	−0.680228	−1.203198	−1.235475
3 C	−1.436098	−0.181761	−0.395835
4 C	−0.894888	0.969089	0.074301
5 N	0.425963	1.284757	−0.261238
6 C	1.263062	0.602828	−1.248037
7 C	2.488256	1.381681	−0.664784
8 C	1.468383	1.948629	0.364747
9 O	1.524029	2.710821	1.296606
10 C	−1.595246	1.992544	0.990893
11 O	−1.955405	1.585459	2.117990
12 O	−1.718634	3.139643	0.500505
2a H	−0.990448	−2.209625	−0.946693
2b H	−0.927544	−1.085612	−2.294157
6 H	1.005866	0.878214	−2.271539
7 H	2.814072	2.154078	−1.361564
1” N	3.665230	0.694430	−0.201838
2” C	3.773060	0.039900	0.968886
3” C	5.111575	−0.564393	1.313095
4” C	6.227823	−0.667219	0.331022
5” N	6.335986	0.262993	−0.670515
6” C	7.393775	−0.000434	−1.413250
7” S	8.232940	−1.427926	−0.819861
8” N	7.104236	−1.638154	0.420695

1” H	4.529956	0.801892	−0.736534
9” O	2.828881	−0.078326	1.768704
10” N	5.378567	−1.056271	2.482408
11” O	4.420727	−1.072902	3.438459
11” H	3.581364	−0.711581	3.032291
12” N	7.768458	0.716186	−2.479873
12”a H	7.265502	1.559762	−2.710486
12”b H	8.615681	0.514718	−2.985060
1’ C	−2.819217	−0.595994	−0.134968
2’ C	−3.955696	0.074341	−0.381052
3’ C	−4.197588	1.426378	−1.001135
4’ C	−5.685062	1.391709	−1.411930
5’ N	−6.229634	0.230454	−0.688688
6’ C	−5.276084	−0.552093	−0.127013
7’ C	−7.665656	−0.050284	−0.666066
8’ C	−8.027366	−1.475414	−1.126440
9’ N	−8.101047	−2.243722	0.160682
10’ C	−8.816544	−1.333794	1.124078
11’ C	−8.291307	0.066749	0.762962
1’ H	−2.941763	−1.614637	0.232211
3’a H	−3.538950	1.616778	−1.849949
3’b H	−4.022443	2.223454	−0.275849
4’a H	−5.812797	1.246242	−2.488828
4’b H	−6.220482	2.296932	−1.121520
12’ O	−5.490658	−1.619570	0.476510
7’ H	−8.122310	0.665510	−1.345595
8’a H	−7.301707	−1.933628	−1.793481
8’b H	−9.015500	−1.492729	−1.583896
9’a H	−8.547234	−3.155556	0.064093
9’b H	−7.112538	−2.351488	0.472615
10’a H	−9.883937	−1.439269	0.937698
10’b H	−8.593370	−1.646183	2.141500
11’a H	−9.102004	0.793476	0.774574
11’b H	−7.533793	0.398965	1.471110

SCF GIAO magnetic shielding (ppm):

1 S	Isotropic = 494.1018 Anisotropy = 207.4144
2 C	Isotropic = 148.0049 Anisotropy = 28.5819
3 C	Isotropic = 66.9107 Anisotropy = 128.3703
4 C	Isotropic = 37.4017 Anisotropy = 145.3884
5 N	Isotropic = 70.7948 Anisotropy = 126.7718
6 C	Isotropic = 115.6783 Anisotropy = 31.8541
7 C	Isotropic = 119.3602 Anisotropy = 36.5251
8 C	Isotropic = 16.1167 Anisotropy = 76.7339
9 O	Isotropic = −49.3797 Anisotropy = 501.6699
10 C	Isotropic = 8.5689 Anisotropy = 91.2437
11 O	Isotropic = −16.7647 Anisotropy = 347.6145

12 O	Isotropic = −9.5556 Anisotropy = 351.8580
2a H	Isotropic = 28.8443 Anisotropy = 9.2351
2b H	Isotropic = 28.4446 Anisotropy = 9.1657
6 H	Isotropic = 26.8154 Anisotropy = 8.0842
7 H	Isotropic = 27.0865 Anisotropy = 5.0854
1” N	Isotropic = 122.2631 Anisotropy = 67.2854
2” C	Isotropic = 12.8807 Anisotropy = 108.4842
3” C	Isotropic = 34.8008 Anisotropy = 134.9052
4” C	Isotropic = 14.9178 Anisotropy = 89.1497
5” N	Isotropic = −1.7585 Anisotropy = 241.7093
6” C	Isotropic = −8.2744 Anisotropy = 129.6801
7” S	Isotropic = 79.2542 Anisotropy = 256.1444
8” N	Isotropic = −22.5353 Anisotropy = 225.3030
1” H	Isotropic = 21.9302 Anisotropy = 20.9643
9” O	Isotropic = −12.3060 Anisotropy = 483.9818
10” N	Isotropic = −214.9954 Anisotropy = 455.0063
11” O	Isotropic = 59.6875 Anisotropy = 320.9323
11” H	Isotropic = 16.0083 Anisotropy = 19.8983
12” N	Isotropic = 166.5761 Anisotropy = 84.2389
12”a H	Isotropic = 25.9624 Anisotropy = 10.9302
12”b H	Isotropic = 26.3918 Anisotropy = 10.3030
1’ C	Isotropic = 44.5051 Anisotropy = 146.3224
2’ C	Isotropic = 41.7515 Anisotropy = 123.2716
3’ C	Isotropic = 154.4149 Anisotropy = 19.4510
4’ C	Isotropic = 130.5635 Anisotropy = 54.0080
5’ N	Isotropic = 89.5312 Anisotropy = 108.3794
6’ C	Isotropic = 4.8129 Anisotropy = 102.1857
7’ C	Isotropic = 123.8697 Anisotropy = 29.9872
8’ C	Isotropic = 127.0016 Anisotropy = 38.8458
9’ N	Isotropic = 181.8654 Anisotropy = 38.9396
10’ C	Isotropic = 131.5414 Anisotropy = 55.6725
11’ C	Isotropic = 149.2413 Anisotropy = 39.5671
1’ H	Isotropic = 25.0511 Anisotropy = 8.2729
3’a H	Isotropic = 29.4426 Anisotropy = 5.8008
3’b H	Isotropic = 28.8293 Anisotropy = 10.1980
4’a H	Isotropic = 28.2829 Anisotropy = 7.0702
4’b H	Isotropic = 28.3083 Anisotropy = 5.9477
12’ O	Isotropic = 32.7639 Anisotropy = 463.2476
7’ H	Isotropic = 27.9235 Anisotropy = 5.9991
8’a H	Isotropic = 28.2891 Anisotropy = 6.9151
8’b H	Isotropic = 28.5101 Anisotropy = 9.9523
9’a H	Isotropic = 27.1102 Anisotropy = 17.0206
9’b H	Isotropic = 21.7413 Anisotropy = 21.0880
10’a H	Isotropic = 28.6290 Anisotropy = 11.1935
10’b H	Isotropic = 27.9786 Anisotropy = 9.2774
11’a H	Isotropic = 29.1052 Anisotropy = 9.6750
11’b H	Isotropic = 29.6657 Anisotropy = 6.5251

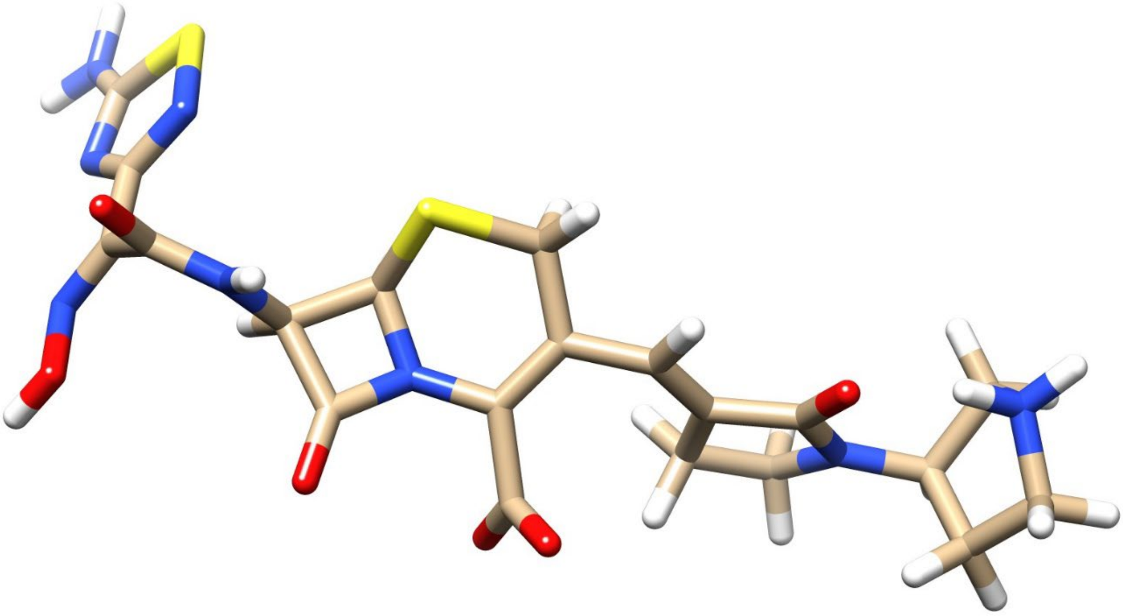

15

Energy = −2461.60635819 Hartree

Atomic coordinates (Å):

	X	Y	Z
1 S	1.072325	0.583888	−1.920641
2 C	−0.664451	0.990656	−1.456314
3 C	−1.429941	−0.065089	−0.669393
4 C	−0.839147	−1.036951	0.067959
5 N	0.558950	−1.051049	0.179672
6 C	1.488624	0.000569	−0.240036
7 C	2.644316	−1.044133	−0.097561
8 C	1.504251	−2.060807	0.189150
9 O	1.440041	−3.257379	0.323145
10 C	−1.550328	−2.182495	0.816621
11 O	−2.308106	−2.916218	0.144534
12 O	−1.275941	−2.256337	2.037974
2a H	−1.172285	1.170944	−2.406040
2b H	−0.657105	1.940144	−0.914256
6 H	1.523750	0.842952	0.450337
7 H	3.246999	−0.850112	0.788324
1” N	3.495965	−1.327954	−1.216144
2” C	4.846868	−1.218368	−1.261211
3” C	5.565887	−0.783704	0.011415
4” C	5.849115	0.650614	0.186104
5” N	6.628955	1.104330	1.211259
6” C	6.707180	2.414839	1.143154
7” S	5.785224	3.047296	−0.226759
8” N	5.314615	1.490902	−0.673510
1” H	3.066919	−1.631862	−2.083098

9” O	5.502639	−1.466438	−2.263252
10” N	6.006078	−1.615150	0.880772
11” O	5.647614	−2.923269	0.581480
11” H	6.025424	−3.439280	1.305628
12” N	7.375478	3.184830	2.015984
12”a H	7.963612	2.737020	2.703092
12”b H	7.517181	4.167707	1.848379
1’ C	−2.880455	0.090882	−0.814119
2’ C	−3.821732	0.160506	0.140313
3’ C	−3.725227	0.101430	1.642139
4’ C	−5.078199	0.660779	2.131712
5’ N	−5.925490	0.655719	0.927775
6’ C	−5.239461	0.412688	−0.215366
7’ C	−7.357187	0.950624	0.999755
8’ C	−7.806763	2.081395	0.055480
9’ N	−8.260046	1.343962	−1.170030
10’ C	−9.035206	0.159840	−0.654775
11’ C	−8.255106	−0.267209	0.601445
1’ H	−3.231621	0.255593	−1.832075
3’a H	−2.878573	0.671567	2.027919
3’b H	−3.598790	−0.928970	1.980845
4’a H	−4.989670	1.682067	2.513836
4’b H	−5.529209	0.040813	2.908167
12’ O	−5.729486	0.423041	−1.359771
7’ H	−7.550902	1.251314	2.026730
8’a H	−7.026440	2.792623	−0.203183
8’b H	−8.667224	2.606987	0.466335
9’a H	−8.793973	1.923115	−1.817572
9’b H	−7.386530	0.999531	−1.622741
10’a H	−10.040836	0.507233	−0.422849
10’b H	−9.083557	−0.599816	−1.431573
11’a H	−8.940603	−0.524046	1.407312
11’b H	−7.631186	−1.137212	0.402731

SCF GIAO magnetic shielding (ppm):

1 S	Isotropic = 495.1185 Anisotropy = 208.2736
2 C	Isotropic = 147.8585 Anisotropy = 29.7116
3 C	Isotropic = 64.3505 Anisotropy = 132.2873
4 C	Isotropic = 37.3939 Anisotropy = 142.7839
5 N	Isotropic = 69.8284 Anisotropy = 120.7823
6 C	Isotropic = 115.8803 Anisotropy = 30.1942
7 C	Isotropic = 117.3161 Anisotropy = 30.8239
8 C	Isotropic = 13.0591 Anisotropy = 76.8638
9 O	Isotropic = −49.7023 Anisotropy = 506.8852
10 C	Isotropic = 8.8055 Anisotropy = 90.4273
11 O	Isotropic = −16.6799 Anisotropy = 351.5566
12 O	Isotropic = −6.9278 Anisotropy = 352.5635

2a H	Isotropic =	28.7488	Anisotropy =	9.3906
2b H	Isotropic =	28.3729	Anisotropy =	8.5605
6 H	Isotropic =	26.9154	Anisotropy =	6.8881
7 H	Isotropic =	27.0998	Anisotropy =	6.4321
1” N	Isotropic =	123.2831	Anisotropy =	121.1232
2” C	Isotropic =	11.8096	Anisotropy =	106.2426
3” C	Isotropic =	27.2221	Anisotropy =	116.1908
4” C	Isotropic =	13.5921	Anisotropy =	85.8530
5” N	Isotropic =	−1.4612	Anisotropy =	260.6471
6” C	Isotropic =	−8.5253	Anisotropy =	125.8224
7” S	Isotropic =	70.6130	Anisotropy =	248.4251
8” N	Isotropic =	−38.4693	Anisotropy =	260.9778
1” H	Isotropic =	25.8791	Anisotropy =	5.4267
9” O	Isotropic =	−68.9293	Anisotropy =	536.4313
10” N	Isotropic =	−159.6961	Anisotropy =	375.2574
11” O	Isotropic =	92.1980	Anisotropy =	145.6185
11” H	Isotropic =	24.3872	Anisotropy =	9.4590
12” N	Isotropic =	168.2055	Anisotropy =	88.4546
12”a H	Isotropic =	26.1994	Anisotropy =	12.2606
12”b H	Isotropic =	26.5488	Anisotropy =	10.1005
1’ C	Isotropic =	44.4021	Anisotropy =	145.4132
2’ C	Isotropic =	41.4931	Anisotropy =	123.4239
3’ C	Isotropic =	154.5807	Anisotropy =	18.8412
4’ C	Isotropic =	130.8387	Anisotropy =	54.4756
5’ N	Isotropic =	89.4614	Anisotropy =	106.8326
6’ C	Isotropic =	4.4014	Anisotropy =	100.5102
7’ C	Isotropic =	123.9566	Anisotropy =	30.0246
8’ C	Isotropic =	127.0462	Anisotropy =	36.2599
9’ N	Isotropic =	182.6700	Anisotropy =	39.0643
10’ C	Isotropic =	130.9320	Anisotropy =	56.0705
11’ C	Isotropic =	149.3789	Anisotropy =	39.5753
1’ H	Isotropic =	24.9842	Anisotropy =	8.5521
3’a H	Isotropic =	29.4562	Anisotropy =	6.3279
3’b H	Isotropic =	28.9561	Anisotropy =	10.0970
4’a H	Isotropic =	28.3538	Anisotropy =	7.0966
4’b H	Isotropic =	28.3778	Anisotropy =	5.8039
12’ O	Isotropic =	34.6917	Anisotropy =	466.1579
7’ H	Isotropic =	27.8198	Anisotropy =	6.0465
8’a H	Isotropic =	28.3772	Anisotropy =	7.1245
8’b H	Isotropic =	28.5645	Anisotropy =	9.8742
9’a H	Isotropic =	27.2494	Anisotropy =	16.8811
9’b H	Isotropic =	21.6656	Anisotropy =	21.2253
10’a H	Isotropic =	28.7310	Anisotropy =	11.0512
10’b H	Isotropic =	28.0053	Anisotropy =	9.2466
11’a H	Isotropic =	29.0925	Anisotropy =	9.7268
11’b H	Isotropic =	29.6449	Anisotropy =	6.3357
